# Mapping the global prevalence, incidence, and mortality of *Plasmodium falciparum* and *Plasmodium vivax* malaria, 2000–22: a spatial and temporal modelling study

**DOI:** 10.1016/S0140-6736(25)00038-8

**Published:** 2025-03-22

**Authors:** Daniel J Weiss, Paulina A Dzianach, Adam Saddler, Jailos Lubinda, Annie Browne, Michael McPhail, Susan F Rumisha, Francesca Sanna, Yalemzewod Gelaw, Juniper B Kiss, Sarah Hafsia, Rubini Jayaseelen, Hunter S Baggen, Punam Amratia, Amelia Bertozzi-Villa, Olivia Nesbit, Joanna Whisnant, Katherine E Battle, Michele Nguyen, Kefyalew Addis Alene, Ewan Cameron, Melissa A Penny, Samir Bhatt, David L Smith, Tasmin L Symons, Jonathan F Mosser, Christopher J L Murray, Simon I Hay, Peter W Gething

**Affiliations:** a Curtin University, Bentley, WA, Australia; b The Kids Research Institute Australia, Nedlands, WA, Australia; c Ifakara Health Institute, Dar es Salaam, Tanzania; d Institute for Disease Modeling, Bill & Melinda Gates Foundation, Seattle, WA, USA; e The Institute for Health Metrics and Evaluation, University of Washington, Seattle, WA, USA; f Nanyang Technological University, Singapore; g University of Western Australia, Crawley, WA, Australia; h University of Copenhagen, Copenhagen, Denmark; i Imperial College London, London, UK

## Abstract

**Background:**

Malaria remains a leading cause of illness and death globally, with countries in sub-Saharan Africa bearing a disproportionate burden. Global high-resolution maps of malaria prevalence, incidence, and mortality are crucial for tracking spatially heterogeneous progress against the disease and to inform strategic malaria control efforts. We present the latest such maps, the first since 2019, which cover the years 2000–22. The maps are accompanied by administrative-level summaries and include estimated COVID-19 pandemic-related impacts on malaria burden.

**Methods:**

We initially modelled prevalence of *Plasmodium falciparum* malaria infection in children aged 2–10 years in high-burden African countries using a geostatistical modelling framework. The model was trained on a large database of spatiotemporal observations of community infection prevalence; environmental and anthropogenic covariates; and modelled intervention coverages for insecticide-treated bednets, indoor residual spraying, and effective treatment with an antimalarial drug. We developed an additional model to incorporate disruptions to malaria case management caused by the COVID-19 pandemic. The resulting high-resolution maps of infection prevalence from 2000 to 2022 were subsequently translated to estimates of case incidence and malaria mortality. For other malaria-endemic countries and for *Plasmodium vivax* estimates, we used routine surveillance data to model annual case incidence at administrative levels. We then converted these estimates to infection prevalence and malaria mortality, and spatially disaggregated administrative-level results to produce high-resolution maps. Lastly, we combined the modelled outputs to produce global maps and summarised tables that are suitable for assessing changing malaria burden from subnational to global scales.

**Findings:**

We found an ongoing plateau in rates of malaria infection prevalence and case incidence within sub-Saharan Africa, with consistent year-on-year improvements not evident since 2015. Due to the concentration of malaria burden in sub-Saharan Africa and the region's rapid population growth relative to other endemic regions, we estimate that 2022 had 234·8 (95% uncertainty interval 179·2–299·0) million clinical cases of *P falciparum* malaria, the most since 2004. Despite these findings, deaths from malaria continued to decline in sub-Saharan Africa and consequently globally after 2015, except for the COVID-19-impacted years of 2020–22. Similarly, progress in reducing *P falciparum* and *P vivax* morbidity outside Africa continued despite stalled progress globally. However, a major malaria outbreak in Pakistan following intense flooding in 2022 resulted in a reversal in this improving trend and contributed heavily to the global total of 12·4 (10·7–14·8) million clinical cases of *P vivax* malaria. Within Africa, we found that the plateau in infection prevalence occurred earlier in more densely populated areas, whereas more sparsely populated regions have continued a trajectory of modest improvement.

**Interpretation:**

The unprecedented investment in malaria control since the early 2000s has averted an enormous amount of malaria burden. However, case incidence rates in Africa have flattened, and with a rapidly growing population at risk, the number of *P falciparum* cases in Africa, and thus globally, is now comparable to levels before the surge of investment. Outside Africa progress against malaria morbidity continued after 2015, but a resurgence of *P vivax* cases in 2022 underscores the fragility of progress against malaria in the face of climatic shocks. COVID-19-related disruptions led to increased malaria cases and deaths, but the impact was less severe than feared, in part because endemic countries continued to prioritise malaria control during the pandemic. Nevertheless, improved tools and strategies remain urgently needed to regain momentum against this disease.

**Funding:**

Bill & Melinda Gates Foundation and Australian National Health and Medical Research Council.

**Research in context**

**Evidence before this study**

Malaria burden estimates are published annually in the World Malaria Report and periodically in the Global Burden of Diseases, Injuries, and Risk Factors Study. These publications show declining global trends in burden from 2005 to 2015 and a stalling in progress since then. Global trends are largely driven by sub-Saharan Africa, where over 90% of malaria cases and deaths occur.

**Added value of this study**

National-level malaria burden estimates are illustrative of broad trends, but they lack spatial granularity and cannot be used to characterise subnational patterns of progress. This study yields new high-resolution global malaria burden maps, updating previous maps that were published in 2019. The new analysis includes methodology for estimating the impact of the COVID-19 pandemic on malaria burden, thereby providing the first spatial estimates of malaria burden during the pandemic up to 2022. Further analysis reveals heterogeneous trends in burden relative to population density, which adds nuance to the generalised narrative of stalling progress. The high-resolution maps produced for this study underpin subnational enumeration of malaria burden, are useful for tracking spatially heterogeneous progress against the disease, and can inform strategic malaria control efforts.

**Implications of all the available evidence**

Due to rising populations in sub-Saharan Africa, the plateau in malaria incidence rates there has led to the highest levels of *Plasmodium falciparum* cases globally since 2004. Despite rising cases in Africa, the slight decrease in deaths suggests that the effectiveness of and access to life-saving treatment are improving. COVID-19 impacts on malaria were modest and short-lived, with most countries returning to pre-pandemic levels of malaria by 2022. These findings present a mixed contemporary picture for malaria worldwide. Although global case totals continue to rise, encouraging progress is evident outside Africa as well as in some settings within Africa. These findings can support continued efforts to evaluate progress and optimally guide responses at global, national, and subnational levels.

## Introduction

In 2019, the Malaria Atlas Project published global estimates and high-resolution maps of malaria burden for the years 2000–17,^1^, ^2^ updating earlier results for infection prevalence and case incidence, and representing the first such global results for malaria mortality. This work illustrated the tremendous progress that had been made in reducing malaria burden since the early 2000s when treatment failure was widespread and the use of interventions to reduce transmission was limited. The years following 2017 have been eventful for the malaria community, with concerns around stagnating progress, growing threats of drug and insecticide resistance, the emergence and spread of malaria parasites with histidine rich protein 2 (HRP2) gene deletions affecting the accuracy of rapid diagnostic tests for malaria, and constrained funding alongside optimism around emerging tools, strengthened partnerships, and increasing sophistication in the response to malaria. Coincident with these concerns and advances, the COVID-19 pandemic threatened to reverse hard-won gains by disrupting malaria control efforts, overburdening health systems, and drawing the attention of public health policy makers away from malaria.^3^ Early in the pandemic, scenario modelling emphasised the potential threat, and the dramatic increases in malaria morbidity and mortality, that could result from disruptions to health system access and potentially damaging disruptions to planned intervention campaigns.^3^, ^4^ However, the effect of the pandemic on malaria burden has not been systematically studied to verify if these efforts to ensure access to antimalarial commodities translated into effective control.

In addition to impacting malaria burden, the COVID-19 pandemic led to delays in conducting nationally representative surveys typically collected by countries in collaboration with the Demographic and Health Surveys (DHS) Program. These surveys provide datasets critical for enumerating and contextualising sociodemographic and health factors and are used widely by decision makers. These surveys also provide inputs for our models estimating *Plasmodium* parasite rate and malaria intervention coverages. Routine surveillance data for malaria were also impacted by the pandemic, with major changes to non-malaria fever rates, patterns of care-seeking behaviour, health system availability, and surveillance system function. These factors combined to make observed trends in reported malaria cases since 2020 more difficult to interpret.

The dominant narrative for global malaria burden since 2018 has been one of stalling progress,^5^, ^6^ evidenced by the flat trends in infection prevalence and case incidence rates following the substantial improvements achieved between 2005 and 2015.^7^, ^8^ This earlier period of progress aligned with the global scale-up of investment in malaria control, characterised by the widespread distribution and use of insecticide-treated bednets, and the adoption of artemisinin-based combination therapies as the first-line treatment in sub-Saharan Africa.^9^ Concerningly, the apparent stall in progress occurred despite sustained levels of funding for antimalarial commodities and the scaling up of newer interventions such as seasonal malaria chemoprevention. Accordingly, research continues to explore new public health strategies, therapies, vaccines, and other novel interventions in hope of continued progress towards reducing morbidity and mortality from malaria.

Factors likely to be contributing to the slow-down in progress against malaria include plateauing global funding, behavioural and phenotypic adaptation of *Anopheles* spp to vector control tools, the effects of climate change on transmission and health systems, and human immune dynamics, among others. The relative contributions of these factors are not well understood and are likely to be complex and geographically varying.^6^ Given this context, it is particularly important to understand not just overall global trends in malaria burden, but also how these trajectories differ across and within regions, by country, and at subnational scales. In this study we present the results of a comprehensive global geospatial modelling exercise that produced updated estimates of infection prevalence, case incidence, and malaria mortality. With these products, we evaluate and contextualise trends in *Plasmodium falciparum* and *Plasmodium vivax* malaria since the year 2000. The results of this research were incorporated within the 2023 World Malaria Report^8^ for high-burden African countries. This research also leverages data sources and methods from the Global Burden of Diseases, Injuries, and Risk Factors Study (GBD), and these results will inform the forthcoming 2023 GBD malaria estimates. However, the mortality estimates presented here are calibrated to GBD 2021 as they were the most recent available results at the time of modelling.

## Methods

### Overview

Our methodological approach for estimating malaria burden remains largely consistent with previous studies.^1^, ^2^, ^9^, ^10^ Additional details for all methods are provided in the appendix (pp 10–45), and flow charts for the modelling framework are shown in the appendix (pp 46–47). In brief, we applied two modelling approaches, one for high-burden countries in sub-Saharan Africa and another for all other malaria-endemic countries (appendix pp 16–19). We subsequently refer to these approaches, respectively, as the cartographic and surveillance models.

### Cartographic model

The cartographic model was applied in high-burden countries of sub-Saharan Africa, which have historically had data quality concerns associated with routine surveillance data. While the routine data from some countries in sub-Saharan Africa have improved considerably in recent years, elsewhere case numbers remain heavily biased by low care seeking, and inconsistent diagnostic and reporting practices. Another factor complicating the widespread use of routine case reports in areas where burden and immunity are high is the distinction between the strict definition of a malaria case used for burden estimation (ie, a requisite level of parasite infection density coincident with clinical malaria symptoms) versus simply a positive rapid diagnostic test. In contrast, we used routine surveillance data in other malaria-endemic countries because parasite rate surveys are much rarer, health-care systems and reporting are generally stronger, and subclinical infections are less common.

The cartographic model relied on (1) geolocated observations of community infection prevalence (ie, *P falciparum* parasite rate; *Pf*PR) as obtained from cross-sectional household surveys or from other eligible studies; (2) modelled geospatial intervention coverages for insecticide-treated bednets,^11^, ^12^ effective treatment with an antimalarial drug,^13^ and indoor residual spraying;^14^ and (3) a set of geospatial covariates related to malaria transmission^15^, ^16^ that included temporally dynamic climatic datasets with a monthly temporal resolution such as temperature and rainfall. A spatiotemporal Bayesian geostatistical model was then used to generate estimates of age-standardised *P falciparum* parasite rate for 2–10-year-olds (*Pf*PR_2–10_) for each 5 × 5 km pixel for each year between 2000 and 2022.^9^ Age standardisation was implemented to ensure comparability within the results given inconsistent age ranges within the response data and the plateau in the age–prevalence relationship found between age 2 years and 10 years. Uncertainty in these estimates was represented by the generation and summarisation of 100 realisations from a Bayesian posterior predictive distribution. These estimates of infection prevalence were then converted to case incidence by age using a relationship defined within an established mathematical model ensemble of three *P falciparum* microsimulation modelling frameworks (EMOD, OpenMalaria, and malariasimulation).^17^, ^18^, ^19^, ^20^ This was done for each pixel and by year, with uncertainty propagated from the original 100 *Pf*PR_2–10_ realisations.

A third model estimated malaria mortality for each pixel based on a previously published approach^10^ and was informed by geolocated observations of malaria mortality as a fractional cause of all deaths. For this approach, malaria cause-of-death fraction observations from GBD were merged with national-level estimates of all-cause mortality^21^ to estimate a geospatially varying malaria case fatality rate. The GBD cause of death estimates include verbal autopsy data, which constitute most of the estimates from sub-Saharan Africa, and higher-quality data from the Child Health and Mortality Prevention Surveillance (CHAMPS) network. This rate was then applied to the malaria clinical incidence rates from the preceding step, while accounting for variations in access to effective malaria treatment and other relevant covariates. Operationally, this consisted of first using our modelled estimates of effective treatment with an antimalarial drug to differentiate treated and untreated case totals, and then applying an untreated case fatality rate (uCFR) to estimate deaths.^2^, ^10^ An important consideration related to the mortality estimation methodology is that it adhered to the “one death, one cause” approach of GBD, and therefore aimed to capture deaths for which malaria was the underlying cause. These estimates did not directly incorporate deaths due to other underlying causes, such as those due to comorbidities frequently present in those who died with a malaria infection.^22^

The results of this cartographic modelling process were then summarised to produce high-resolution maps of infection prevalence, case incidence, and deaths for *P falciparum*, as well as population-weighted, national-level and subnational-level estimates for all years, and all metrics are reported with associated estimates of uncertainty (95% uncertainty intervals [UI]). Validation statistics for the Africa *Pf*PR model are presented in the appendix (p 22).

### Surveillance model

The surveillance modelling methodology is described in depth elsewhere for *P falciparum*^2^ and *P vivax.*^1^ In brief, response data consisted of routine surveillance reports of clinical malaria cases collected by governmental agencies in endemic countries and made publicly available through WHO, via online data portals, or within published reports. Before modelling, the surveillance data were adjusted to account for factors including treatment-seeking rates,^23^, ^24^ treatment in the private sector (which might not be captured in public sector data), reporting completeness, and presumptive diagnosis.^25^ Timeseries models were used to impute estimated incidence in years without data. The timeseries model was a generalised additive model, which was fitted with spatial random effects to leverage information from countries within the same region. Predictor variables used in the timeseries model consisted of administrative-level metrics calculated as part of the GBD study (appendix p 32). The timeseries models produced case incidence rate estimates for all surveillance countries, and these values were used to infer infection prevalence by applying the inverse of the prevalence to incidence conversion.^20^ Validation statistics for the surveillance model are presented in the appendix (p 39).

The conversion from case incidence rates to malaria mortality rates followed a similar approach to the cartographic model and entailed first deriving a uCFR^10^ from GBD cause of death estimates. We then multiplied our incidence estimate by one minus the rate of effective treatment to produce an estimate of untreated cases. Finally, we multiplied untreated incidence by the uCFR per country-year to derive deaths. In contrast, *P vivax* deaths, which are very rare in comparison to those from *P falciparum*, were modelled separately using cause of death points that occurred in countries that reported malaria deaths despite only having *P vivax* transmission. The combined cartographic and surveillance malaria mortality estimates were scaled to align with the all-cause mortality envelope derived as part of the GBD. Due to the higher-frequency demand for prevalence and incidence estimates relative to full cycles of the GBD, the mortality estimates were synced with earlier case incidence estimates than those presented here and thus might not fully reflect very recent changes observed in incidence, particularly for countries that have released new national parasite rate surveys or recently experienced large malaria outbreaks.

We produced high-resolution maps of infection prevalence, case incidence, and malaria mortality for surveillance countries using a spatial disaggregation approach. This involved parameterising a downscaling model by associating environmental and anthropogenic covariates with spatially heterogeneous subnational patterns of burden.^2^, ^26^ As with the cartographic approach, predictor variables for the surveillance model consisted of datasets that characterise malaria habitat, in particular environmental datasets such as temperature, precipitation, and vegetation cover. Although rare in comparison to cartographic countries, the limited parasite rate survey data available from surveillance countries were also incorporated in the surveillance model. This was achieved by creating modelled infection prevalence surfaces, which were used as covariates in the model. Lastly, the high-resolution cartographic and surveillance maps were combined to create global mosaics, for all study years, to provide mean and uncertainty estimates for each 5 × 5 km pixel in every malaria-endemic country.

### Data

All input datasets for the cartographic, surveillance, and intervention models were updated using a combination of systematic literature reviews, ingesting data from online sources, acquiring national surveys when they became available through DHS and other organisations, and via personal communication with collaborative partners including WHO. The resulting databases of malaria metrics^27^, ^28^, ^29^ included 82 415 prevalence points (appendix pp 13–14) and 91 337 administrative-level records for routine surveillance data for the years 2000–22, and gridded environmental covariates at a monthly resolution since 2000.^15^ The cause of death data underpinning the mortality model are from the GBD and consist of 4750 unique location-years for malaria endemic countries from 2000 to 2022, 280 of which are from sub-Saharan Africa.^21^ All covariate data and response data for which we have permission to share are freely accessible via the *malariaAtlas* R package.^27^ All data and analysis used in this work are GATHER compliant, with details available at the Global Health Data Exchange.

### Model adaptations for COVID-19

A new aspect of our modelling framework since 2019 is the development of an approach to account for disruptions to treatment-seeking behaviour caused by the COVID-19 pandemic.^30^ The model was parameterised using information extracted from four rounds of the WHO Pulse surveys.^31^, ^32^ In the Pulse surveys, national experts are asked to quantify the level of health-care disruption their countries experienced in the preceding 3–6 months. Although imperfect, a thorough literature review^30^ confirms that these data are the most consistent and readily available measures of health-care disruption for malaria-endemic countries in sub-Saharan Africa for 2020–22. For cartographic countries (ie, moderate-to-high-transmission countries in sub-Saharan Africa), disruptions were integrated into our analysis using an established methodological framework for assessing the impacts of interventions on malaria burden^9^ and for predicting the effects of reducing or cancelling distribution campaigns for antimalarial commodities because of the pandemic.^3^ This approach consisted of modifying our estimated rates of treatment seeking for fever, which we used as a proxy indicator for treatment seeking for malaria in sub-Saharan Africa.^24^ The adjusted treatment-seeking rates were then combined with estimates of proportional antimalarial drug use and effectiveness^33^ to derive estimates of effective treatment with an antimalarial drug. Adjusted malaria case incidence rates were estimated using our geostatistical modelling framework after substituting the COVID-19-adjusted effective treatment layers for the years 2020–22. Malaria mortality estimates, which we quantified by intersecting uCFR with estimated case incidence, after adjusting for effective treatment, are influenced by changes in both case incidence and the proportion of untreated cases that could progress to severe malaria and death. Because most insecticide-treated bednet and indoor residual spraying campaigns went ahead as planned during the pandemic, we did not consider COVID-19 impacts to malaria from these interventions. For lower-burden countries modelled using the surveillance approach, we did not apply a post-hoc adjustment to reflect pandemic-related malaria burden. This decision was based on the assessment that existing surveillance data from these countries, after standard adjustments for reporting completeness and updated treatment seeking, more accurately reflected actual trends during the pandemic period than the broad health-care disruption categories present in the Pulse survey.

### Analysis of changing malaria burden relative to population density

To further contextualise results and increase their utility to policy makers, we summarised malaria burden estimates by administrative level and related these estimates to measures of population density. Metrics of population density were derived from WorldPop^34^ and were used to characterise the malaria burden along an urban–rural spectrum.^35^ The analysis was conducted for the period from 2000 to 2020 to provide four equal intervals from which to assess changes in *P falciparum* prevalence in Africa. These intervals approximately correspond to the period before scale-up of interventions and artemisinin-based combination therapy (2000–05), two periods of rapid scale-up (2005–10 and 2010–15), and the onset of stalling progress (2015–20). Urbanicity was selected as an analytical frame because it correlates with key metrics of development within a country such as the level of poverty, educational attainment, and access to health-care services.^36^, ^37^, ^38^ We used population density as a proxy for urbanicity because it is a standardised metric and therefore avoids reliance on subjective and geographically varying definitions of urban versus rural. We related the measures to *Pf*PR rates through time at subnational levels to explore trends in burden across this important axis of development.

### Description of results

Our results consist of administrative and 5 × 5 km resolution estimates of *P falciparum* and *P vivax* malaria prevalence, *P falciparum* and *P vivax* case incidence, and *P falciparum* mortality in all malaria-endemic countries. We illustrate these results for 2022; high-resolution maps and data are accessible for all years 2000–22 on the Malaria Atlas Project website. Prevalence estimates are presented as the proportion of the population with malaria in a given year. For incidence and mortality, we provide both count (number of new cases or deaths per year) and rate (number of new cases or deaths per 1000 [incidence] or 100 000 [mortality] people per year). Due to the rarity of deaths caused by *P vivax*, and the lack of consensus on its mortality rate, death estimates for *P vivax* were only derived for country-years in which only *P vivax* cases were reported. In these instances, *P vivax* death estimates were typically less than one and thus are not presented here. In addition to the maps, administrative-level summaries of these products are available regionally, nationally, and subnationally. All mapped and tabular estimates are accompanied by measures of uncertainty. Geospatial covariates and modelled intervention coverages used for burden estimation can also be accessed through our website or the *malariaAtlas* R package.^27^

### Role of the funding source

The funder of the study had no role in study design, data collection, data analysis, data interpretation, or writing of the report.

## Results

**Figure 1:**
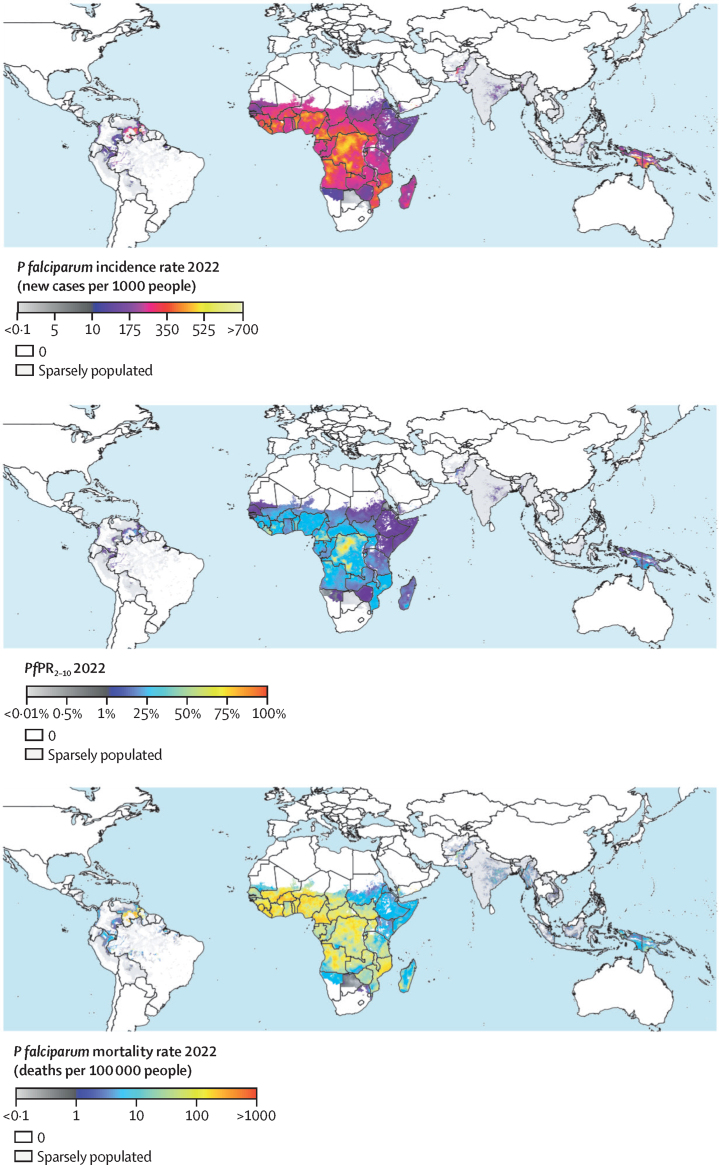
*Plasmodium falciparum* burden maps for 2022, showing all-age clinical incidence rate (top), infection prevalence in 2–10-year-olds (middle), and all-age mortality rate (bottom) *Pf*PR_2–10_= *P falciparum* parasite rate for 2–10-year-olds.

**Figure 2:**
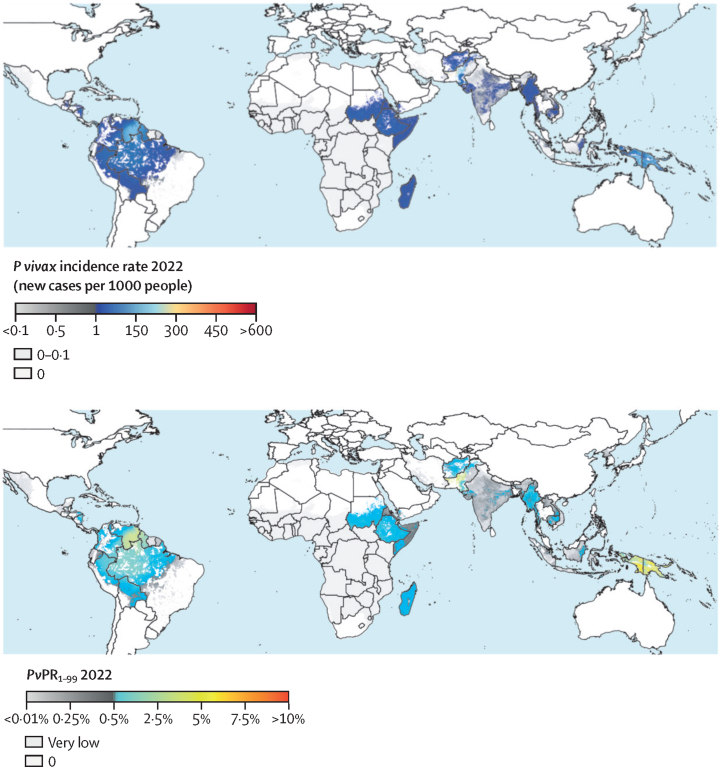
*Plasmodium vivax* burden maps for 2022, showing all-age clinical incidence rate (top) and infection prevalence in 1–99-year-olds (bottom) *Pv*PR_1–99_=*P vivax* parasite rate for 1–99-year-olds.

There were 85 countries with ongoing malaria transmission in 2022, down from 105 countries in 2000. In 2022, we estimate that there were 244·4 (95% UI 188·1–308·5) million *P falciparum* cases globally, 96·0% (234·8 million) of which occurred in sub-Saharan Africa (appendix p 7). We estimate that *P falciparum* led to 697·2 (246·2–1456·8) thousand deaths, with sub-Saharan Africa disproportionately bearing 94·8% (661·1 thousand) of the global total. The burden of *P vivax* was considerably lower, with 12·43 (10·70–14·83) million cases globally in 2022. The spatial pattern of *P vivax* burden differed greatly from that of *P falciparum*, with 84·3% (10·48 million) of cases occurring outside sub-Saharan Africa (appendix p 7). However, except for several countries in east Africa, the burden of *P vivax* in Africa remains poorly measured^39^, ^40^ due to the preponderance of individuals in other regions of Africa who are negative for the Duffy red blood cell receptor and thus resistant to *P vivax*. As a result, *P vivax* might be under-represented in our results for African countries. Maps of *P falciparum* and *P vivax* burden in 2022 are shown in figure 1 and figure 2, and maps of our 2022 coverages for effective treatment of antimalarial drugs, indoor residual spraying, and insecticide-treated bednets are provided in the appendix (pp 4–6).

**Figure 3:**
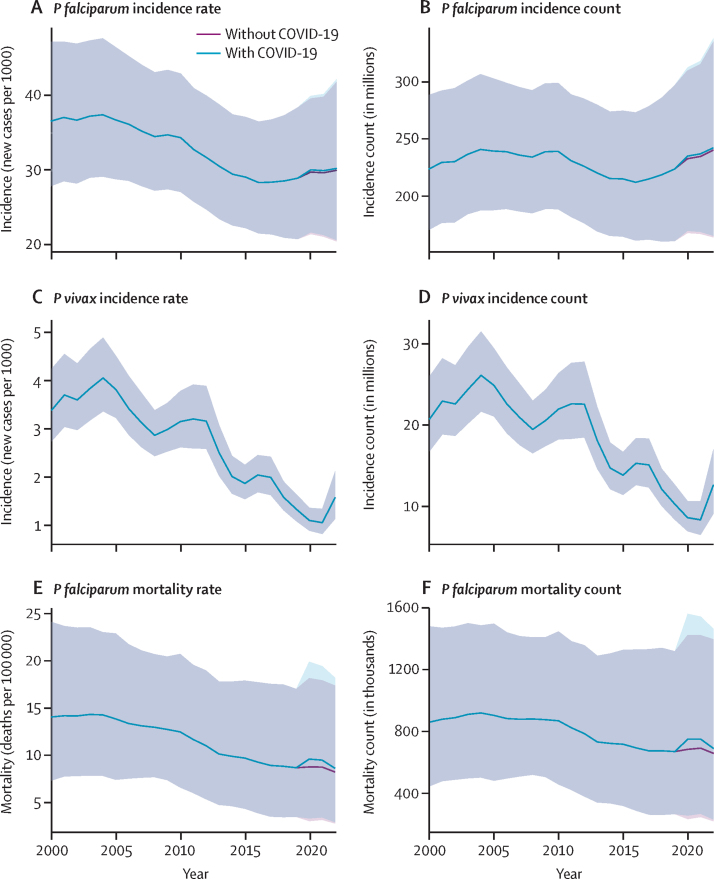
Estimates of global malaria burden from 2000 to 2022 with 95% uncertainty intervals (A) *P falciparum* clinical incidence rate (new cases per 1000 people per year). (B) *P falciparum* clinical incidence count (new cases per year, millions). (C) *P vivax* clinical incidence rate (new cases per 1000 people per year). (D) *P vivax* clinical incidence count (new cases per year, millions). (E) *P falciparum* mortality rate (deaths per 100 000 people per year). (F) *P falciparum* mortality count (deaths per year, thousands). The light blue line shows estimates that include the effect of disruptions to effective treatment coverage which arose due to the COVID-19 pandemic, while the purple line represents a counterfactual scenario of malaria burden in the absence of disruptions to effective treatment coverage. No COVID-19 counterfactual line is shown for *P vivax* because the COVID-19 adjustments were only done in sub-Saharan Africa where the burden of *P vivax* is poorly understood. Shaded areas show 95% uncertainty intervals.

Our results provide a comprehensive update and extend previously published malaria burden estimates^1^, ^2^ by 5 additional years (2018–22). Table 1 and figure 3 present all-age malaria case incidence and mortality estimates across the study period. The *P falciparum* time-series show a decline in the rates of prevalence, incidence, and deaths from approximately 2005 to 2015, followed by a period of minimal change in incidence and gradual decrease in deaths until 2020, when the COVID-19 pandemic led to a modest increase in deaths (figure 3). These findings support WHO's narrative of stalled progress^6^, ^7^ in reducing the proportion of people who contract malaria each year. Because the population of malaria-endemic countries in sub-Saharan Africa grew from 0·94 billion in 2000 to 1·68 billion in 2022, however, the corresponding global case counts have grown considerably from their study-period lows in 2016–17. As a result, we estimate 2022 to have had the greatest number of *P falciparum* cases since 2004. This trend will likely continue until incidence rates decline at a rate that surpasses population growth. Furthermore, the global incidence rate shows a gradual increase after 2015 because of the increasing proportion of the global population residing within Africa where malaria incidence rates remain stable. As such, the global proportion of *P falciparum* cases occurring in Africa increased from 91·9% (207·7 of 226·0 million) in 2000 to 96·0% (234·8 of 244·4 million) in 2022 (appendix p 7). Outside of sub-Saharan Africa, the situation is more optimistic for *P falciparum*, as countries generally maintained progress and continued to reduce rates of both incidence and death after 2015.

**Table 1:** Mean malaria all-age incidence and mortality with 95% uncertainty intervals

	***P falciparum* incidence rate (new cases per 1000 people per year)**	***P falciparum* incidence count (new cases per year, millions)**	***P vivax* incidence rate (new cases per 1000 people per year)**	***P vivax* incidence count (new cases per year, millions)**	**Mortality rate (deaths per 100 000 people per year)**	**Mortality count (deaths per year, thousands)**
**Global**						
2000	37·0 (32·1–42·7)	226·0 (196·1–261·0)	3·4 (3·1–3·7)	20·8 (19·1–22·7)	14·2 (7·5–24·3)	865·1 (457·3–1485·1)
2005	37·4 (32·7–44·0)	243·6 (213·1–286·4)	3·8 (3·5–4·1)	24·8 (23·0–26·9)	13·9 (7·6–23·1)	908·6 (494·3–1507·5)
2015	29·6 (24·6–35·7)	218·6 (182·0–264·2)	1·8 (1·7–2·0)	13·7 (12·5–14·7)	9·8 (4·6–18·0)	723·1 (338·4–1332·7)
2019	29·2 (23·5–35·7)	225·5 (181·3–275·7)	1·3 (1·2–1·4)	10·3 (9·6–11·0)	8·8 (3·6–17·1)	677·0 (280·1–1317·9)
2022	30·5 (23·5–38·5)	244·4 (188·1–308·5)	1·6 (1·3–1·9)	12·4 (10·7–14·8)	8·7 (3·1–18·2)	697·2 (246·2–1456·8)
**Sub-Saharan Africa**						
2000	306·5 (262·4–359·1)	207·7 (177·8–243·3)	3·8 (2·2–5·6)	2·6 (1·5–3·8)	112·8 (59·9–192·5)	764·6 (406·0–1304·5)
2005	293·0 (253·6–348·4)	227·3 (196·7–270·2)	6·2 (4·6–8·0)	4·8 (3·6–6·2)	105·3 (57·1–173·7)	816·3 (443·1–1346·9)
2015	205·1 (168·9–250·4)	207·7 (171·0–253·6)	3·3 (2·5–4·2)	3·3 (2·6–4·2)	63·8 (29·8–117·7)	646·5 (302·2–1191·6)
2019	195·3 (155·7–240·0)	218·9 (174·6–269·1)	1·2 (0·9–1·4)	1·3 (1·0–1·6)	57·0 (23·9–110·2)	639·1 (267·6–1235·5)
2022	192·9 (147·2–245·7)	234·8 (179·2–299·0)	1·6 (1·3–2·0)	2·0 (1·5–2·4)	54·3 (20·2–109·7)	661·1 (245·5–1334·9)
**Outside sub-Saharan Africa**						
2000	3·4 (3·2–3·6)	18·3 (17·1–19·5)	3·3 (3·1–3·6)	18·2 (17·1–19·6)	1·9 (0·9–3·3)	100·5 (51·3–180·6)
2005	2·8 (2·7–3·0)	16·3 (15·4–17·3)	3·5 (3·2–3·7)	20·0 (18·6–21·1)	1·6 (0·9–2·8)	92·3 (51·2–160·6)
2015	1·7 (1·6–1·9)	10·9 (10·1–11·9)	1·6 (1·5–1·7)	10·4 (9·7–11·1)	1·2 (0·6–2·2)	76·6 (36·2–141·1)
2019	1·0 (0·9–1·1)	6·6 (6·0–7·2)	1·4 (1·3–1·5)	9·0 (8·4–9·8)	0·6 (0·2–1·3)	37·9 (13·3–82·4)
2022	1·4 (1·3–1·6)	9·7 (8·5–10·9)	1·5 (1·3–1·9)	10·5 (8·7–12·7)	0·5 (0·0–1·8)	36·2 (0·7–121·9)

Data in parentheses are 95% uncertainty intervals. Selected years include the start and end of the study period, the scaling up of interventions in 2005, the start of the stalled progress period in 2015, and pre-pandemic in 2019.

Deaths from *P falciparum* have not risen as sharply as cases (figure 3, table 1), except for a slight increase associated with reduced treatment seeking during the COVID-19 pandemic. This outcome is driven by the increasing levels of effective treatment found in high-burden countries in sub-Saharan Africa, as well as ongoing improvements in management of severe malaria cases. These factors caused the number of untreated cases to remain broadly flat and led to a corresponding plateau of malaria deaths, thereby counterbalancing the impact of increasing in *P falciparum* cases. It is also important to note that the results from the pandemic period have greater uncertainty than preceding years due to the coincident disruptions in national parasite rate surveys (ie, with little prevalence and intervention data available for fitting our models, the confidence intervals around our mean estimates widened).

Outside of Africa, the spatiotemporal patterns of *P vivax* burden were similar to those of *P falciparum*. There was a declining global trend for *P vivax* case incidence and infection prevalence for the years 2015–21. In 2022, however, major flooding in Pakistan led to a large outbreak of malaria, with 66·8% (1·22 of 1·82 million) of confirmed cases caused by *P vivax.*^8^ The impact of the flooding on *P vivax* case incidence was profound, with Pakistan's estimated cases increasing from 1·91 (95% UI 1·38–2·47) million in 2021 to 5·81 (3·89–8·15) million in 2022. This 305% increase in Pakistan, which already had the highest national total of *P vivax* cases in 2021, led to a 49·0% increase in the global case incidence of *P vivax* from 8·34 (7·57–9·12) million cases in 2021 to 12·43 (10·70–14·83) million cases in 2022. As a result, in 2022, Pakistan alone accounted for 46·8% of estimated global *P vivax* case incidence, and the increase in *P vivax* led to global infection rates and cases incidence being even higher in 2022 than in 2018.

We generated a counterfactual scenario to the cartographic model that estimated the impact of COVID-19 on malaria in high-burden African countries. This scenario assumed that the effective treatment of malaria was fully maintained during the COVID-19 pandemic (figure 3). We estimate that pandemic-related disruptions to treatment seeking led to modest increases in *P falciparum* incidence of 1·0%, 1·0%, and 0·8%, in the years 2020, 2021, and 2022, respectively (table 2). However, because treatment seeking also plays a role in preventing uncomplicated *P falciparum* cases from potentially progressing to severe disease and eventual death, we estimate that mortality across sub-Saharan Africa increased 9·6%, 8·4%, and 4·8% in 2020, 2021, and 2022, respectively (table 2). Over the course of 2020–22, the cumulative impact of these disruptions was an additional 175·4 (60·7–364·9) thousand malaria deaths relative to our counterfactual estimates. A table of country-specific COVID-19 impacts is shown in the appendix (pp 23–24).

**Table 2:** Comparison of estimated *Plasmodium falciparum* burden in cartographic countries for years 2020–22 with and without COVID-19 disruptions to effective treatment coverage

	**Incidence count (new cases per year, millions)**			**Mortality count (deaths per year, thousands)**		
	With COVID-19 disruptions	No disruptions	Percentage change of mean	With COVID-19 disruptions	No disruptions	Percentage change of mean
2020	230·5 (183·9–283·8)	228·2 (181·9–281·3)	1·0%	725·5 (260·1–1496·6)	662·2 (237·5–1366·2)	9·6%
2021	230·9 (184·8–288·1)	228·7 (182·9–285·5)	1·0%	715.4 (277·7–1419·3)	659·9 (256·2–1309·2)	8·4%
2022	234·8 (179·2–299·0)	232·9 (177·7–296·5)	0·8%	661·1 (245·5–1334·9)	630·9 (234·3–1274·0)	4·8%

Data in parentheses are 95% uncertainty intervals.

**Figure 4:**
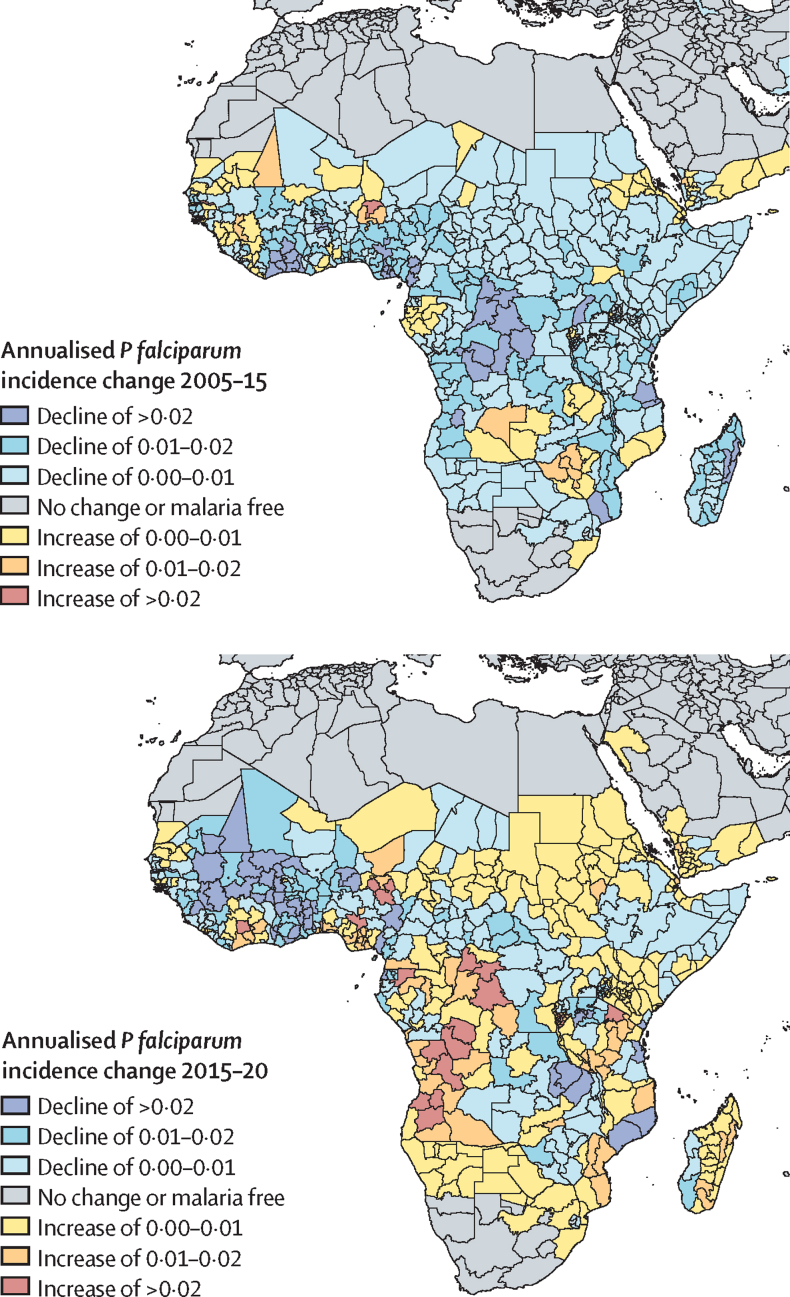
Maps of annualised *Plasmodium falciparum* case incidence changes in Africa between 2005 and 2015 (top) and 2015 and 2020 (bottom) Displayed values were calculated as the change in population-weighted case incidence rate (ie, new cases per 1000 in the end year minus new cases per 1000 in the start year) and annualised by dividing this difference in case incidence rate by the number of years in the period.

Figure 4 shows annualised change in *P falciparum* incidence rates in Africa for the periods 2005–15 and 2015–20. This period was selected to avoid potential confusion with COVID-19 impacts because relatively few post-pandemic surveys existed at the time of the analysis and the data underpinning the COVID-19 adjustments were imprecise. The shifting spatiotemporal patterns of malaria burden provide a nuanced perspective for interpreting the narrative of stalled progress on a continental level. We found that declines in malaria incidence occurred throughout most endemic areas in Africa between 2005 and 2015. In contrast, the 2015–20 period was more variable despite continued progress in many countries in west Africa. In other areas, including the two highest malaria burden countries of Nigeria and the Democratic Republic of the Congo, progress was uneven. Although malaria infection rates declined in some parts of those countries, they rose in others. These complex patterns of change between and within countries further highlight the importance of tailored approaches to malaria control, with country-led subnational stratification of strategies adapted to local contexts.^41^ However, some countries such as Central African Republic had little response data throughout the timeseries, while others, including the Democratic Republic of the Congo, Uganda, Malawi, Sudan, and South Sudan, had little to no data since 2018.

**Figure 5:**
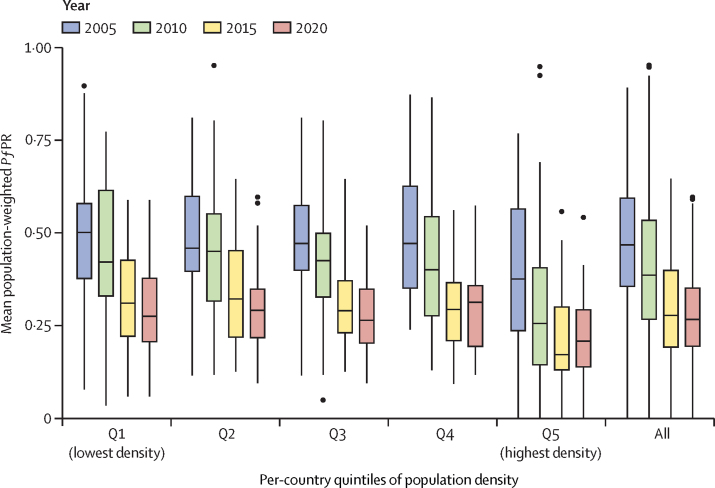
*Pf*PR summarised at the level of the largest subnational administrative unit (eg, state) per population density quintiles for the years 2005–20 among the highest burden countries in sub-Saharan Africa, which represent 70% and 74% of the global malaria burden in 2005 and 2020, respectively Boxplots for each time period represent the distribution of population-weighted mean *Pf*PR across all administrative units belonging to each country's population density stratum (eg, the lowest population density quintiles from each country are combined to make the Q1 estimates). The horizontal line within each box is the median and the whiskers are the data values lying within threshold values (quartile 1 – 1·5 × IQR and quartile 3 + 1·5 × IQR); the black dots represent outliers. *Pf*PR=*Plasmodium falciparum* parasite rate.

In addition to spatial variation in progress against *P falciparum* malaria between and within administrative regions, progress also varied along a population density gradient that we used as a proxy for the region's level of urbanicity. Figure 5 illustrates this relationship for high-burden African countries, with corresponding figures for low-burden African countries and malaria-endemic countries outside Africa presented in the appendix (pp 8–10). Our analysis reveals that lower population density areas in the high-burden countries of sub-Saharan Africa generally continued to reduce their rates of malaria incidence during the 2015–20 period, although the decline was less pronounced than in earlier years. In contrast, more densely populated areas of these countries experienced increases in malaria burden during 2015–20. Furthermore, we observed that in 2005, the difference in malaria burden among the density quintiles was relatively low. In the period of great strides against malaria (2005–15), densely populated areas experienced greater declines in burden than sparsely populated areas. By the 2015–20 period, progress had stalled or reversed in more densely populated administrative units, but sparsely populated areas continued to improve. As a result, by 2020, malaria burden across the density quintiles was more uniform, resembling the levels seen in 2005.

## Discussion

Our global malaria burden estimates for 2000–22 indicate a concerning trend of faltering progress in reducing the global burden of the disease, with the number of clinical cases of *P falciparum* malaria in 2022 estimated as the highest since 2004. However, our results also highlight the importance of understanding local trends. Many areas of Africa showed progress against malaria during the 2015–20 period whereas others experienced setbacks. We also emphasise the importance of distinguishing trends in African urban versus rural areas. Rural areas have generally shown more consistent downward trends in recent years, although they typically continue to have burden levels higher than their urban counterparts. Urban areas, particularly the most densely populated ones, have lower absolute levels of malaria risk, but the fact they are trending in the wrong direction further underscores the importance of recent efforts to tackle urban malaria with renewed approaches.^42^ One possible explanation for these findings is that the full impact of the intervention strategies that led to the reductions in the 2005–15 period might simply have been slower to reach rural areas. Another hypothesis is that endemic countries might have shifted their strategic focus from urban and peri-urban areas to rural locations where malaria burden is generally higher. Emerging threats such as *Anopheles stephensi*, an invasive species of urban-dwelling mosquito spreading across Africa, and insecticidal and antimicrobial resistance must also be considered. Regardless of the drivers, these findings add important context to the slowdown in progress at the continental level and highlight the importance of identifying the factors driving or hindering progress in different locations. The interplay between trends in malaria burden and malaria control efforts should be explored through deeper analyses that adequately account for heterogeneous epidemiological, ecological, political, and cultural contexts. Such analyses could inform ongoing stratification efforts to identify optimal mixes of interventions that are tailored to meet local conditions.

The precise impacts on global malaria burden of control strategies post-2020, including the greater emphasis on subnational stratification and tailoring, remain unclear due to data limitations associated with the COVID-19 pandemic. However, the few recent *Pf*PR surveys that are available from 2020–22, and thus included in our modelling, suggest that malaria incidence rates are returning to pre-pandemic levels. Our new estimates with updated methods to account for impacts of COVID-19 during 2020–22 align with the limited survey data, as we estimated a modest overall impact of COVID-19 on malaria morbidity and mortality. Furthermore, while we estimate that COVID-19 did increase malaria mortality, the worse-case scenarios envisioned at the start of the pandemic^3^ have been avoided by a concerted effort from malaria-endemic countries, where most malaria intervention campaigns went ahead despite additional challenges. However, these results are statistically less certain than those for the preceding years due to the limited number of *Pf*PR surveys collected during the pandemic. This limitation is of greatest concern in sub-Saharan Africa, where there is great reliance on these cross-sectional surveys and where most of the global malaria burden lies. Factors other than the pandemic, including natural disasters^43^ and armed conflict,^44^ could also substantially impact health-care access, but were not considered in this analysis.

There are several noteworthy limitations associated with our methodological approach for generating malaria burden estimates. Most new parasite rate data used as the response variable in the cartographic model were obtained from national surveys because the availability of prevalence data from other sources (eg, peer-reviewed publications) is declining. Although national surveys provide very rich spatial information on parasite rate and intervention coverages, they are typically collected infrequently, and large gaps between survey years are common. Our increasing reliance on national surveys has several additional drawbacks, including the potential to miss malaria outbreaks in our estimates if they are not associated with contemporaneous parasite rate samples, and the vulnerability of our modelling approach to disruptions of planned surveys like those resulting from the COVID-19 pandemic. The surveillance method also has limitations related to the response data from routine case reports. These reports typically only capture data from individuals who seek care at public health-care facilities, while data from those who seek care in the private sector or fail to seek care are absent from the reports. Routine case reports might also be incomplete, including years with no data and other years with incomplete facility reporting. In our modelling approach, we adjusted for these missing malaria cases,^25^ but relying on estimates for treatment seeking rates, reporting completeness, and the proportion of public versus private treatment seeking introduces potential sources of error in our results. Lastly, we did not produce sex-specific results amenable for characterising differences in malaria burden by sex.

The cause of death data that we acquired from the GBD and relied on for estimating malaria deaths also have limitations. In particular, the quantity, quality, and untimeliness of data from sub-Saharan Africa might reduce the accuracy of our corresponding uCFR estimates. Our results suggesting that death trends remained flat since 2017 despite rising case incidence are driven by gradually improving rates of effective treatment for malaria, which prevent untreated case incidence from experiencing a corresponding rise. These untreated cases are converted to deaths using our uCFR estimates, which remain flat through this period. An important caveat, however, is that most of the cause of death data from GBD consist of verbal autopsy data collected before 2010, with the most recent available data for sub-Saharan Africa from 2019. As such, the data used to parameterise the relationship between uCFR and sociodemographic and environmental covariates might not reflect recent changes such as those brought about by the COVID-19 pandemic. Despite these substantial limitations, no reliable alternative sources estimating for malaria deaths are currently available, nor are independent mortality datasets with which to support our finding of a flat trend in deaths.

The modelling framework does not, at present, directly incorporate the impact on malaria burden of seasonal malaria chemoprevention, or currently deployed malaria vaccines such as RTS,S. Their impact on malaria burden is partially accounted for in those countries where *Pf*PR surveys have been conducted contemporaneously with these interventions, but too few such surveys exist to parameterise these interventions within our modelling framework and thus reliably extrapolate their impacts to non-surveyed locations. Furthermore, in countries that conduct seasonal malaria chemoprevention, children younger than 5 years are both targeted for the intervention and are the population typically sampled for malaria in national surveys. As a result, lowered age-specific prevalence in young children among countries that implement seasonal malaria chemoprevention might decrease the accuracy of our prevalence to incidence conversion. Work to incorporate these interventions more explicitly within the malaria burden estimation modelling is underway.

Trends in *P falciparum* outside of Africa and *P vivax* globally do not adhere to the overall narrative of stalling progress and continued to improve through the 2015–21 period. These findings suggest that most malaria-endemic countries are continuing to make progress towards elimination. However, a flooding event in Pakistan in 2022 reversed the global gains made against *P vivax* over the preceding 4 years. This outbreak illustrates the fragility of progress made against malaria in the face of climatic shocks. As such, more research is needed to estimate the potential impacts of catastrophic weather events in malaria-endemic regions that are likely to increase in frequency and severity as they are exacerbated by climate change.^45^ While the timing of such events is inherently unpredictable, identifying changing patterns of risk through time and the areas of greatest vulnerability will be crucial for developing strategies that mitigate future malaria outbreaks.

In conclusion, although the global burden of malaria remains unacceptably high and progress against the disease has been limited since 2015, there remain reasons for optimism within the results we present here. The resolve of malaria-endemic countries to proceed with intervention campaigns despite the pandemic demonstrated the resilience of malaria control programmes. These actions helped prevent a substantial escalation in cases and deaths, suggesting that, when implemented, such efforts largely continue to maintain a status quo in terms of prevalence, incidence, and mortality rates. New strategies have emerged in malaria control, such as subnational intervention tailoring based on risk stratification. When these strategies are based on robust estimates of past and current burden and intervention coverages, they have the potential to deliver more effective control and restart progress towards eliminating the disease. Maintaining progress, however, will require adaptive, data-driven health policies to address emerging challenges ranging from political instability to climatic shocks.

### Contributors

### Data sharing

All pixel-level and administrative-level summaries are available for visualisation and download from https://data.malariaatlas.org/. Most geospatial covariates and modelled intervention coverages are also available through the *malariaAtlas* R package (https://cran.r–project.org/web/packages/malariaAtlas/index.html).

## Declaration of interests

We declare no competing interests.
