# Supplementary material for: Mapping the global prevalence, incidence, and mortality of Plasmodium falciparum and Plasmodium vivax malaria, 2000–22: a spatial and temporal modelling study
Source: Lancet. 2025 Mar 22;405(10483):979–90. doi: 10.1016/S0140-6736(25)00038-8 (PMC11928297; doi:10.1016/S0140-6736(25)00038-8)
Supplement: Supplementary appendix [file mmc1.pdf]

THE LANCET

Supplementary appendix

This appendix formed part of the original submission and has been peer reviewed. We post it as supplied by the authors.

Supplement to: Weiss DJ, Dzianach PA, Saddler A, et al. Mapping the global prevalence, incidence, and mortality of *Plasmodium falciparum* and *Plasmodium vivax* malaria, 2000–22: a spatial and temporal modelling study. *Lancet* 2025; published online March 5. https://doi.org/10.1016/S0140-6736(25)00038-8.

Mapping the global prevalence, incidence, and mortality of *Plasmodium falciparum* and *Plasmodium vivax*, 2000–2022

Daniel J. Weiss[1,2], Paulina A. Dzianach[2], Adam Saddler[2], Jailos Lubinda[2], Annie Browne[2], Michael McPhail[2], Susan F. Rumisha[2,3], Francesca Sanna[2], Yalemzewod Gelaw[1,2], Juniper B. Kiss[2], Sarah Hafsia[2], Rubini Jayaseelen[2], Hunter S. Baggen[2], Punam Amratia[2,3], Amelia Bertozzi-Villa[4], Olivia Nesbit[5], Joanna Whisnant[5], Katherine E. Battle[4], Michele Nguyen[6], Kefyalew Addis Alene[1,2], Ewan Cameron[1,2], Melissa Penny[2,7], Samir Bhatt[8,9], David L. Smith[5], Tasmin L. Symons[1,2], Jonathan F. Mosser[5], Christopher J. L. Murray[5], Simon I. Hay[5], Peter W. Gething[1,2]

[1] Curtin University, Bentley, WA, Australia
[2] The Kids Research Institute Australia, Nedlands, WA, Australia
[3] Ifakara Health Institute, Dar es Salaam, Tanzania
[4] Institute for Disease Modeling, Bill & Melinda Gates Foundation, Seattle, WA, USA
[5] The Institute for Health Metrics and Evaluation, University of Washington, Seattle, WA, USA
[6] Nanyang Technological University, Singapore
[7] University of Western Australia, Crawley, WA, Australia
[8] University of Copenhagen, Copenhagen, Denmark
[9] Imperial College London, London, United Kingdom

Correspondence to: Dr Daniel J. Weiss, Curtin University, Bentley, WA, Australia and
The Kids Research Institute Australia, Nedlands, WA, Australia (dan.weiss@curtin.edu.au)

Table of Contents

1 Extended figures 4

2 Morbidity 10

2.1 Data 10

2.1.1 Raster covariates 10

2.1.2 Population data 11

2.1.3 PR data collection 12

2.1.4 Treatment-seeking data assembly 15

2.1.5 Surveillance data collection 15

2.2 Methods 20

2.2.1 Prevalence to incidence conversion (Cartographic approach) 20

2.2.2 Incidence to prevalence conversion (Surveillance approach) 21

2.2.3 Africa prevalence model 22

2.2.3.1 Africa prevalence model validation 22

2.2.4 Treatment-seeking model 22

2.2.4.1 Model approach 22

2.2.4.2 COVID adjustments 23

2.2.5 API estimation 24

2.2.5.1 API formulae 24

2.2.5.2 Calculating the proportions of *P. falciparum* and *P. vivax* cases from raw data 25

2.2.5.3 Reported testing regimes 25

2.2.5.4 Reported number of confirmed malaria cases in a year (C) 25

2.2.5.5 Reported number of unconfirmed cases in a year (U) 26

2.2.5.6 Reporting completeness (r) 27

2.2.5.7 Slide positivity rate (s) 27

2.2.5.8 Population figures 27

2.2.5.9 Treatment-seeking figures (p, n, a) 27

2.2.5.10 Special considerations for India 28

2.2.5.11 Special considerations for countries in Elimination Phase 29

2.2.5.12 Outlier removal 29

2.2.5.13 Post-hoc masking 30

2.2.6 Surveillance country time-series models 31

2.2.6.1 National time-series 31

2.2.6.1.1 Special case: Malaysia *P. knowlesi* 32

2.2.6.2 Subnational time-series 32

2.2.6.2.1 Special case: Brazil 33

2.2.6.2.2 Special case: India 33

2.2.6.3 Surveillance model validation 39

2.2.7 Surveillance country disaggregation regression 39

2.2.7.1 Machine learning with PR points 39
2.2.7.1.1 Model validation 39
2.2.7.1.2 Models and hyperparameters 39
2.2.7.2 Disaggregation regression 40
2.2.7.2.1 Data 40
2.2.7.2.2 Model definition 40
2.2.7.2.3 Likelihood definition 41
2.2.7.2.4 Priors 41
2.2.7.2.5 Weighting by admin level 41
2.2.7.2.6 Computational model fitting 42
2.2.7.2.7 Temporal Interpolation 42
2.2.7.2.8 Masking and population 42
2.2.7.2.9 Bootstrap uncertainty 42
2.2.7.2.10 Age splitting 43
3 Mortality 43
3.1 Data 43
3.2 Methods 44
3.2.1 Mortality estimation in cartographic and surveillance countries 44
3.2.1.1 Untreated incidence 44
3.2.1.2 Case fatality rate 44
3.2.1.3 Deriving mortality estimates 45
3.2.2 Age splitting mortality results 45
3.2.3 Raking death estimates to match GBD results 45
4 Schematic diagrams 46
5 GATHER compliance 48
5.1 Checklist 48
6 References 49

1 Extended figures

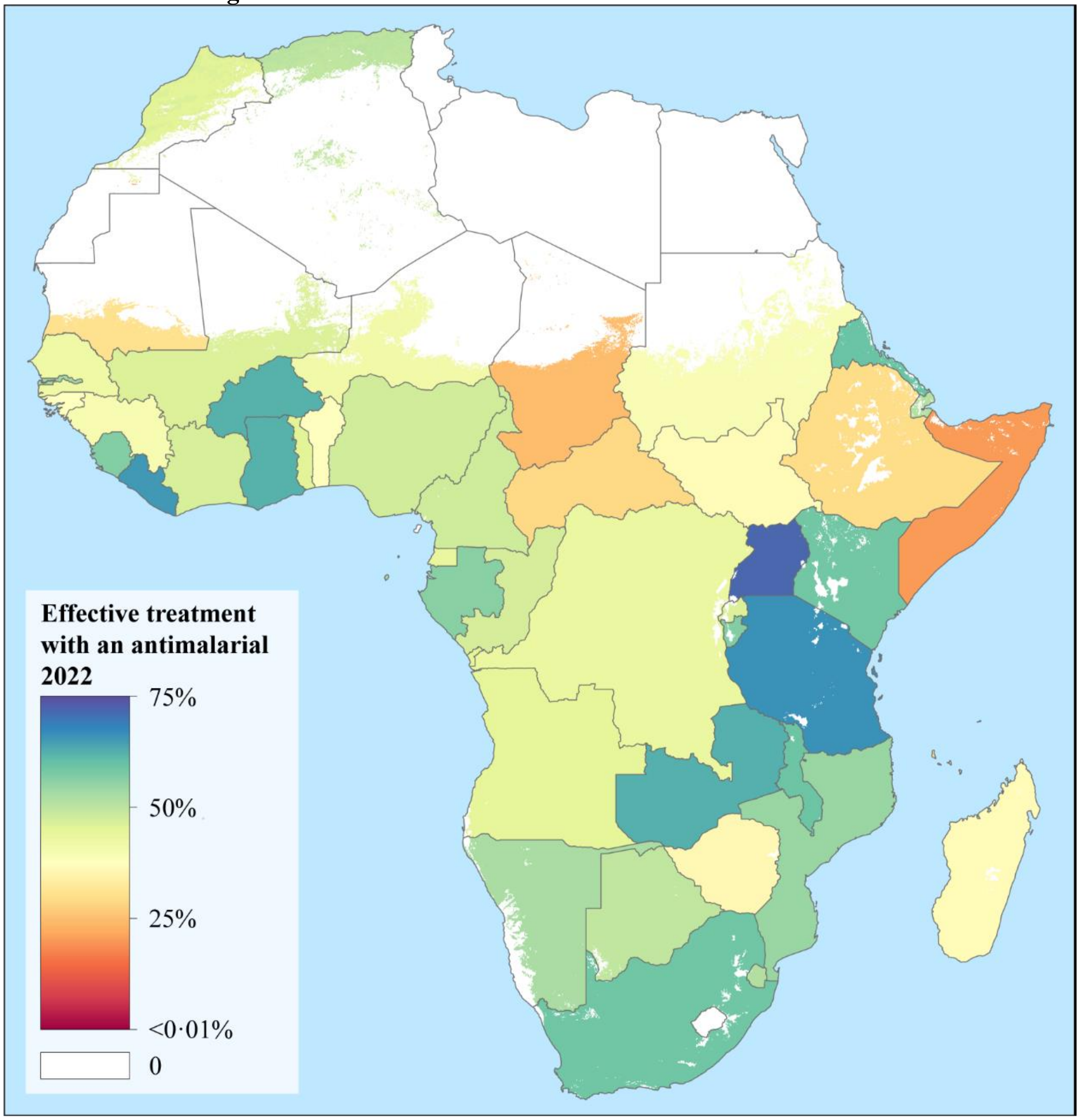

Figure S1. Percentage of effective treatment with an antimalarial drug in 2022.

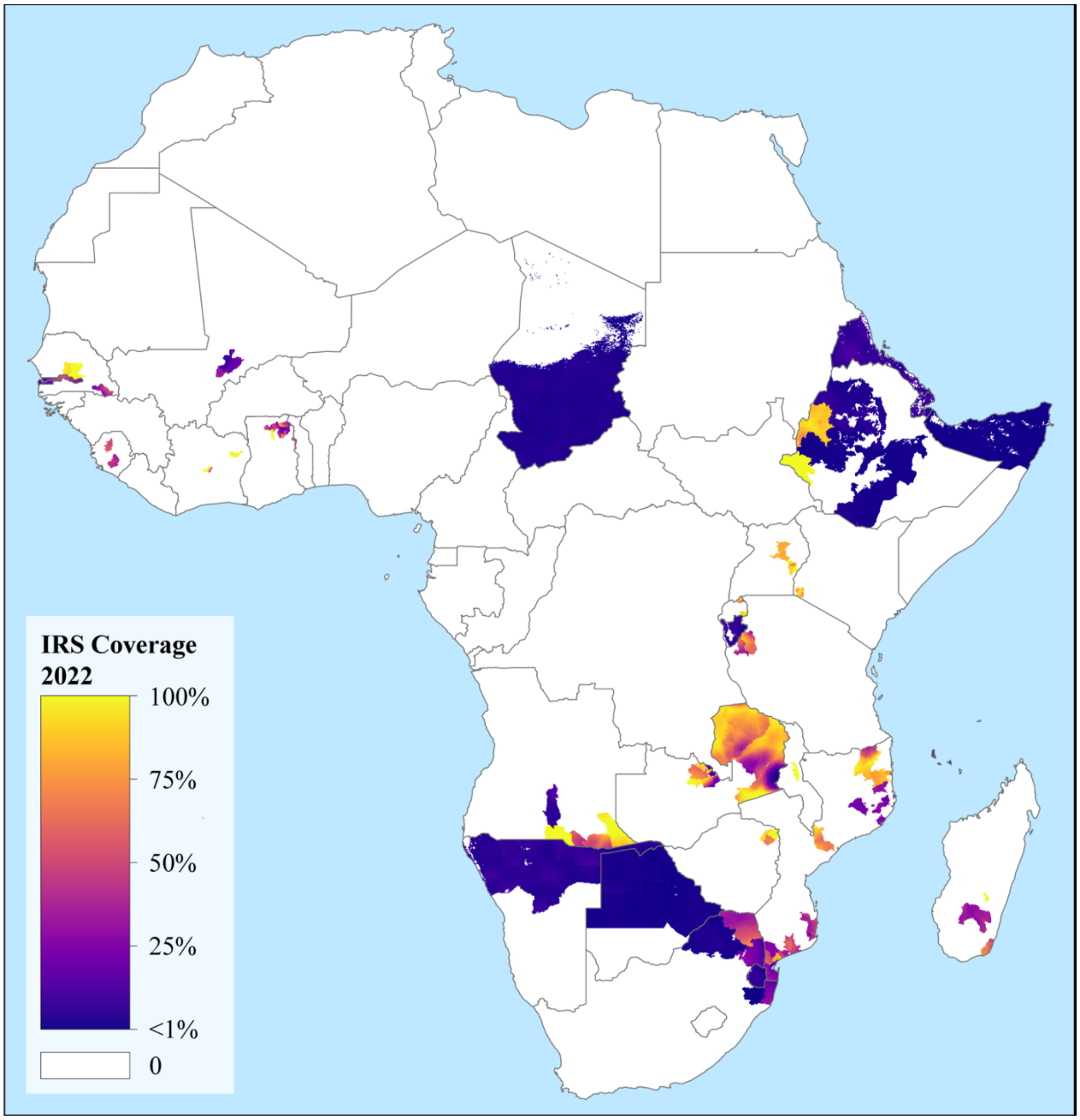

Figure S2. Percentage of households sprayed by indoor residual insecticide in 2022.

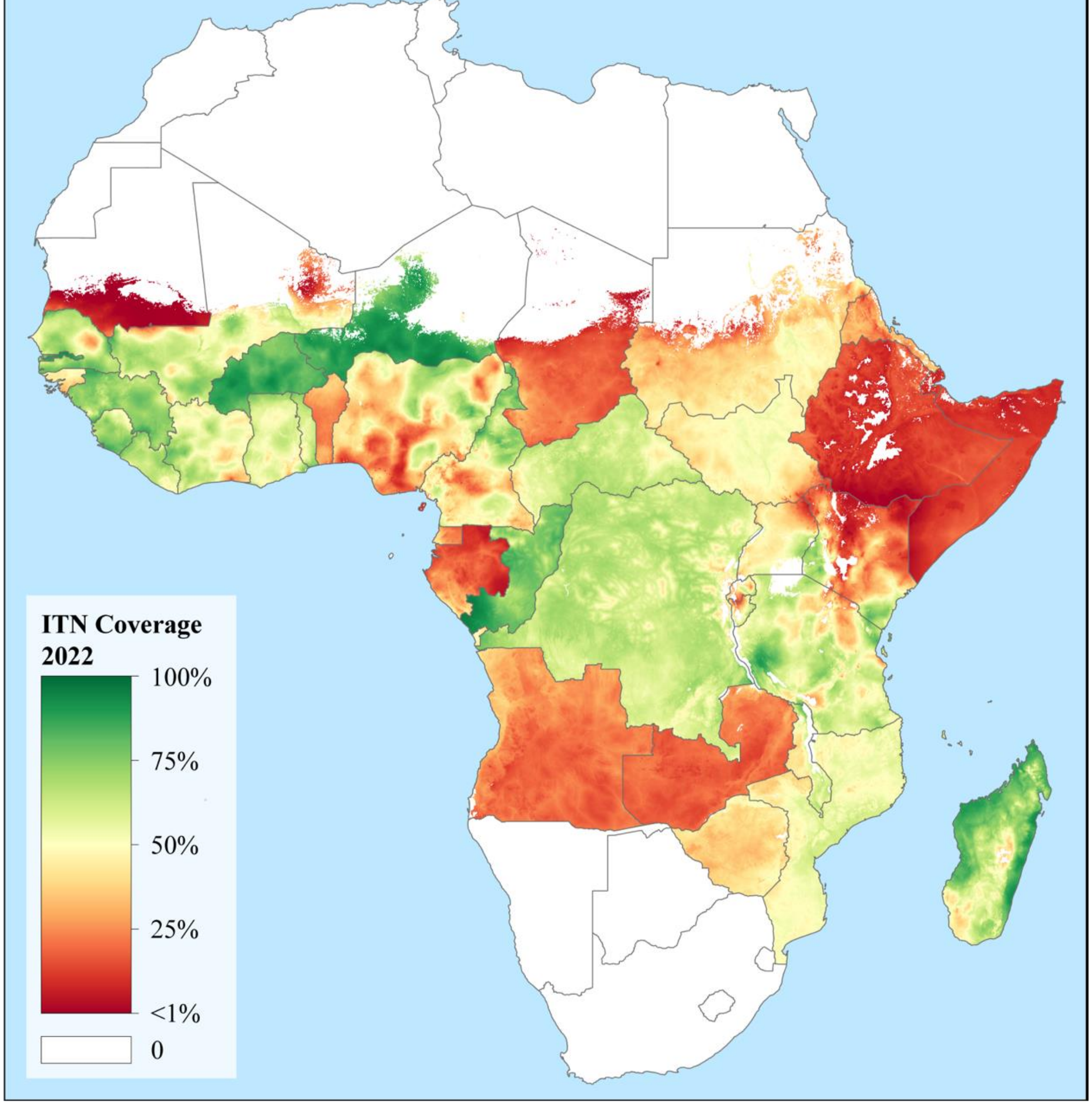

Figure S3. Percentage of insecticide treated bednet use in 2022.

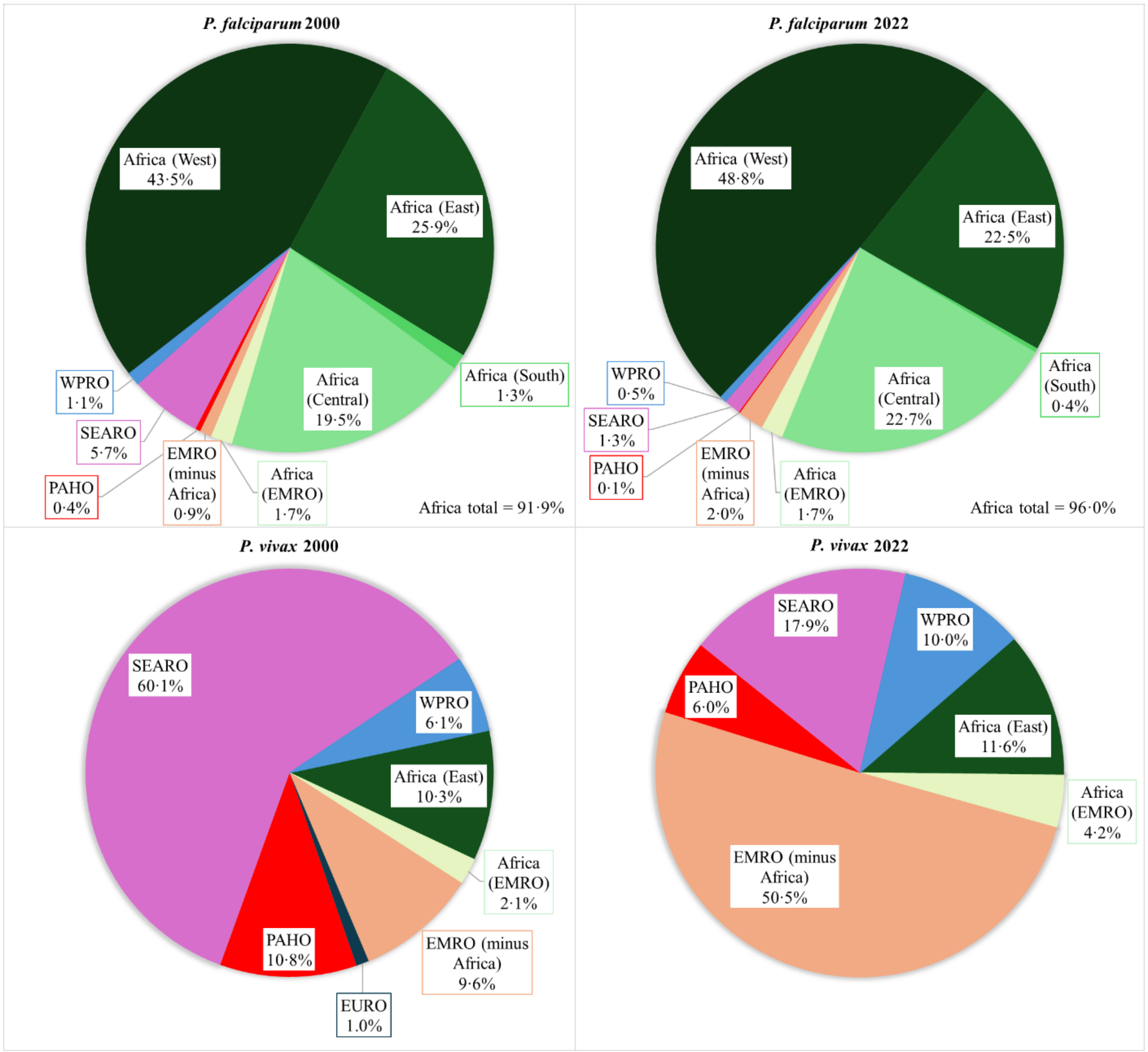

Figure S4. Proportional distribution of *P. falciparum* and *P. vivax* case incidence among WHO regions in 2000 and 2022.

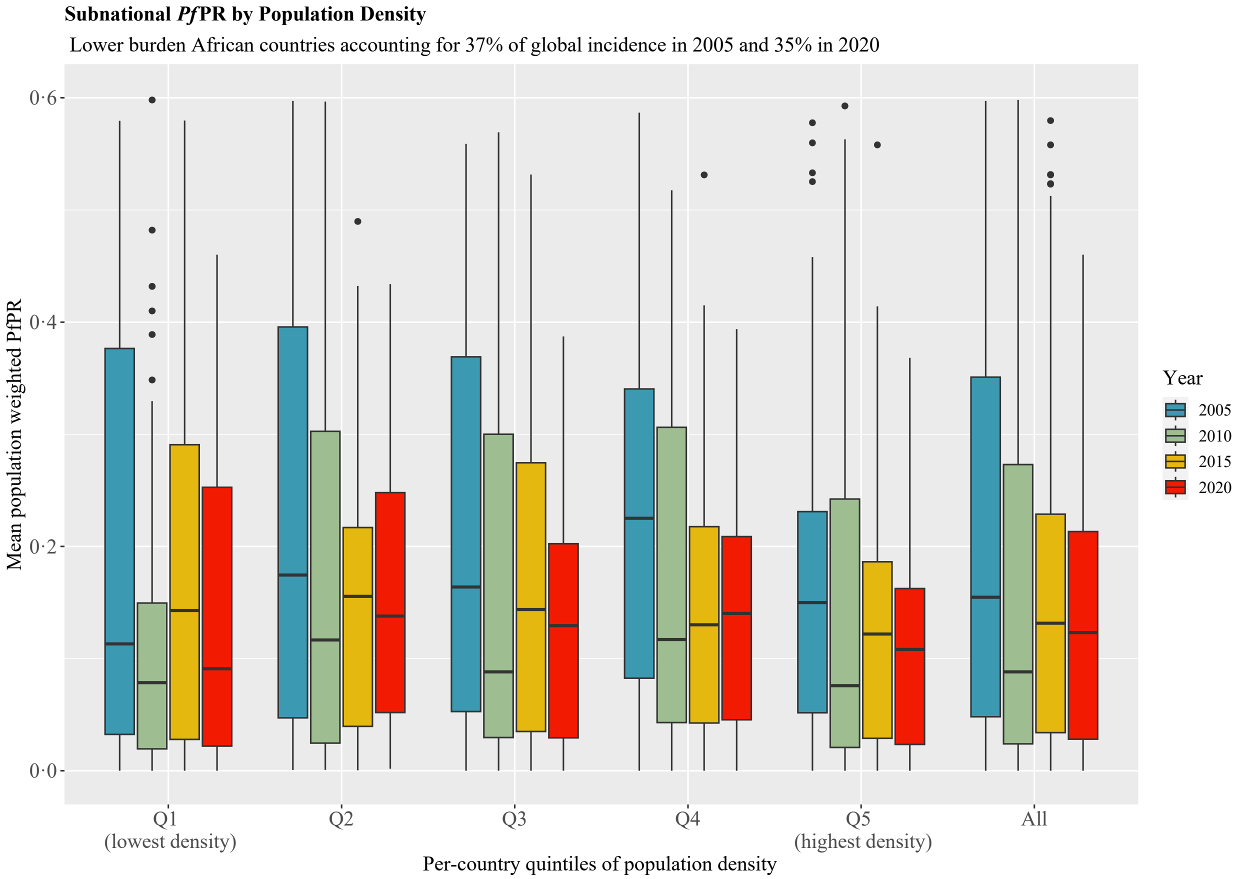

Figure S5. *Pf*PR summarised at the admin-1-level per population density quintiles for years 2005-2020 for lower burden countries in Africa where 23% of global *P. falciparum* occurred in both 2005 and 2020.

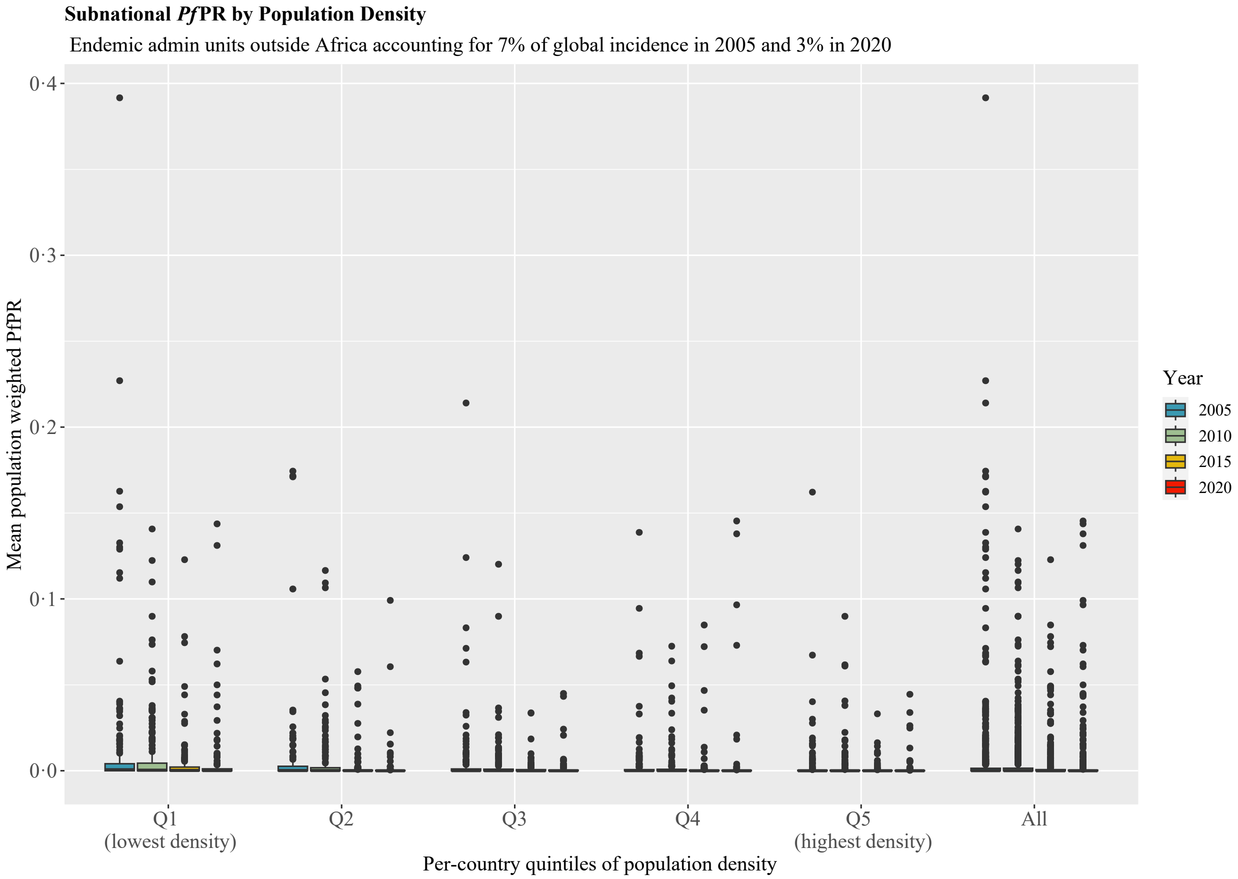

Figure S6. ***Pf*****PR summarised at the admin-1-level per population density quintiles for years 2005-2020 for countries outside of Africa where 7% of global** ***P. falciparum*** **occurred 2005 and 3% occurred in 2020.**

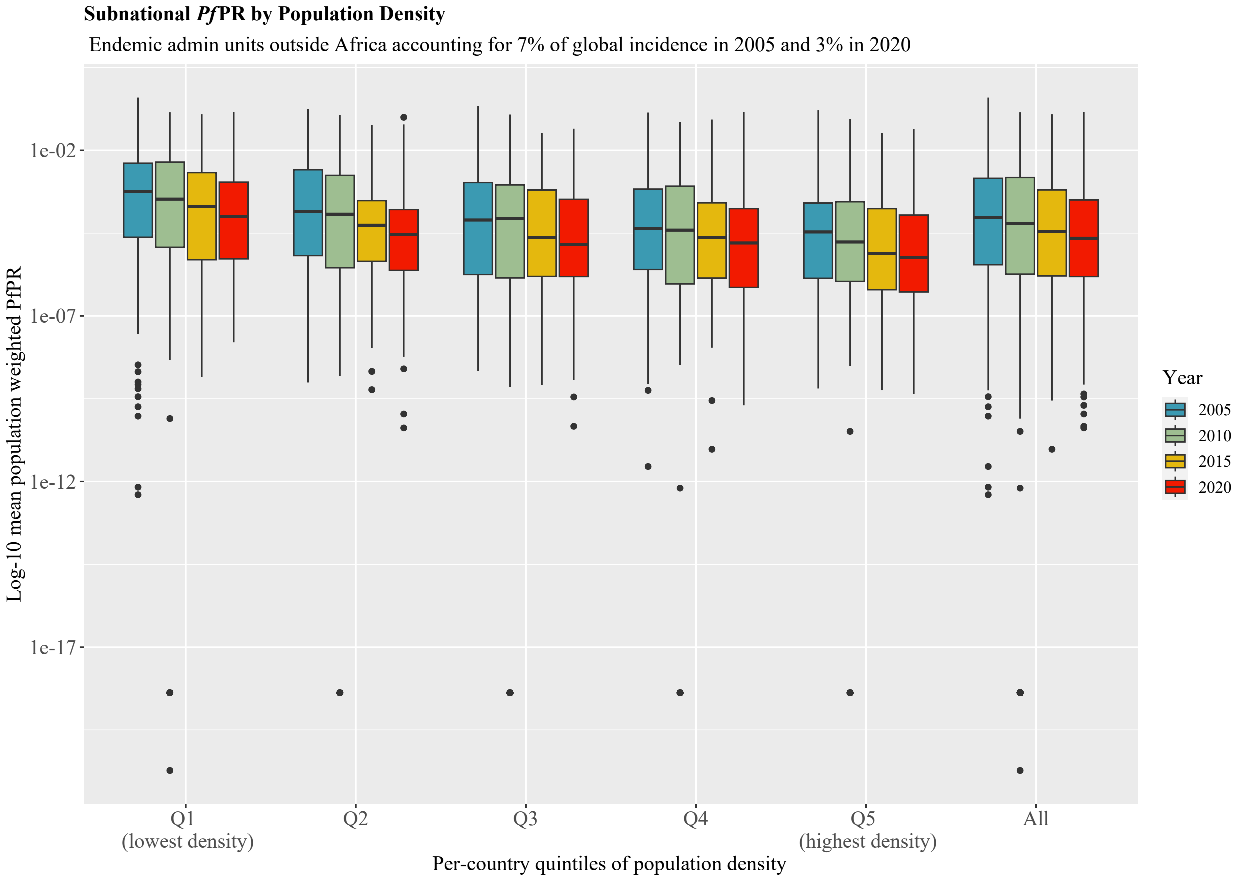

Figure S7. *Pf*PR summarised at the admin-1-level per population density quintiles for years 2005-2020 for countries outside of Africa where 7% of global *P. falciparum* occurred in 2005 and 3% occurred in 2020. The data is identical to what was shown in figure S6, but with the y-axis changed to log-10 to illustrate changes at very low *Pf*PR levels.

2 Morbidity

2.1 Data

2.1.1 Raster covariates

Fourteen of rasterised environmental and anthropological covariates at 2·5 arcminute (approximately 5km × 5km) resolution were used for modelling. The covariates and their associated references and processing notes are given in the table below.

Table S1. Covariates used.

Covariate	Source	Processing
IGBP Combined Forest	MODIS MCD12Q1[1]	Process 1
EVI Mean	MCD43D62, MCD43D63, and MCD43D64[2]	Process 2
EVI SD	MCD43D62, MCD43D63, and MCD43D64[2]	Process 2
LST Daytime Annual Mean	MODIS MOD11A2[3]	Process 2
LST Daytime Annual SD	MODIS MOD11A2[3]	Process 2
LST Night Annual Mean	MODIS MOD11A2[3]	Process 2
LST Night Annual SD	MODIS MOD11A2[3]	Process 2

TCB Annual Mean	MCD43D62, MCD43D63, MCD43D64, MCD43D65, MCD43D66, MCD43D67, and MCD43D68[2]	Process 2
TCB Annual SD	MCD43D62, MCD43D63, MCD43D64, MCD43D65, MCD43D66, MCD43D67, and MCD43D68[2]	Process 2
Precipitation	WorldClim[4]	Overall local mean
Accessibility	Weiss et al.[5]	2·5 arcminute spatial mean
Nighttime lights (2010 stable lights)	VIIRS v2.1[6]	2·5 arcminute spatial mean
Elevation	SRTM 3 arcsecond Digital Elevation Model[7]	2·5 arcminute spatial mean
CGIAR-CSI Global PET Database	Zomer et al.[8]	2·5 arcminute spatial mean

The processes referred to in the table above are:

- Process 1: The MODIS MCD12Q11[1] data were downloaded for the closest available relevant year (2013: the data are not available for subsequent years) and the IGBP landcover band was extracted, reprojected, and merged to a global lat/lon GeoTIFF grid at 15 arcsecond (approximately 500m) resolution. The various IGBP classes representing types of forest (e.g., evergreen needleleaf forest, evergreen broadleaf forest, etc.) were selected and reclassified to a single forest/not-forest grid. This grid was then aggregated by a factor of 10 to 2·5 arcminute (approximately 5km) resolution, where the output cell value represents the percentage of the 100 input cells that were classified as any forest.
- Process 2: The MODIS MCD43D (BRDF reflectance)[2] and MOD11A2 (land surface temperature)[3] products are available at an 8-daily interval. These were downloaded for the entire period of data availability (2000-2022) and were converted to GeoTIFFs for the relevant metric by extracting the relevant bands and performing the necessary calculations to convert to the required indices such as EVI, before reprojecting and merging to global lat/lon GeoTIFFs at 30 arcsecond resolution. All of these grids were then gap-filled using the algorithm published by Weiss et al.[9] These 8-daily 30 arcsecond grids were then aggregated to 8-daily 2·5 arcminute (taking the spatial mean value of the 25 source pixels) and then those were aggregated temporally to annual summaries (using the spatial mean and SD values derived from the 25 source pixels).

2.1.2 Population data

Population estimates were provided by the Institute of Health Metrics and Evaluation (IHME), the University of Washington, Seattle. The estimates were derived as part of the Global Burden of Disease (GBD) project[10] and were provided at national level for all countries and at administration level one for Kenya, Ethiopia, South Africa, Nigeria, Pakistan, Iran, Philippines, Brazil, India, China, Mexico, and Indonesia.
Initial global raster surfaces of population were created using the 1km UN-adjusted country-specific datasets for 2000-2020 from WorldPop, merged and aggregated to 2·5 arcminute resolution using a sum.[11]
For each modelled year, age, sex breakdown provided within GBD, a raster of the population was then created by distributing the GBD population figures for country/administrative units across the pixels bounded by each country/administrative unit, in the same proportions as the corresponding pixels in the WorldPop raster for the corresponding year. That is, for a given GBD administrative unit for which a population figure was available, we calculated the total of the pixels in the initial raster for that year, divided this by the corresponding GBD figure for that administrative unit / year, and then divided the value of all pixels in that administrative unit by the resulting number to ensure that the total of the pixels matched the GBD figure. As GBD values were available for years prior to 2000 but initial population grids were not, for years prior to 2000 we used the 2000 grid as the initial value. Similarly, for years post-2020, the 2020 WorldPop grid was used as the initial value. In order to create a complete surface, population data from the UN World Population Prospects (https://population.un.org/wpp/Download/Standard/Population/) was used for areas not provided by GBD (French Guiana, Western Saharah and Mayotte). This process produced rasters matching total administrative population values from GBD (or the UN) with the pixel-level population values determined by the proportions of the initial rasters.
MAP has previously published a global limits layer indicating areas where transmission of malaria is highly unlikely.[12] This layer was based on environmental factors, travel guidelines, and statements by the countries regarding their malaria-endemic status in 2010. An amended version of this global limits layer was created excluding the malaria-endemic status of the country. This exclusion was necessary because the research project included data extending back to 2000 during which time the status of many countries has changed. This new global

limit layer was applied over the GBD-adjusted population rasters to set population values in pixels outside the limits of transmission to be zero, resulting in population-at-risk grids. A rasterised version of our MAP Admin geometry files was used to get zonal sums of population from the GBD population rasters, to provide administrative-level population totals.

2.1.3 PR data collection

Plasmodium falciparum and *Plasmodium vivax* parasite rate (*Pf*PR and *Pv*PR) data were from geopositioned community-based survey measurements of parasite rate (PR) available from surveys including those conducted by the Demographic and Health Survey (DHS) program, and those identified through periodic literature searches from published data sources and direct communication with malaria specialists for unpublished measurements of *Pf*PR. The resulting *Pf*PR dataset for years 2000-2022 consisted of n=66148 data points, while the *Pv*PR dataset consisted of n=16267 points. The spatiotemporal coverage of the PR dataset is illustrated in Figure S8 and S9. Further details of the collation of this data can be found in previous publications.[13, 14]

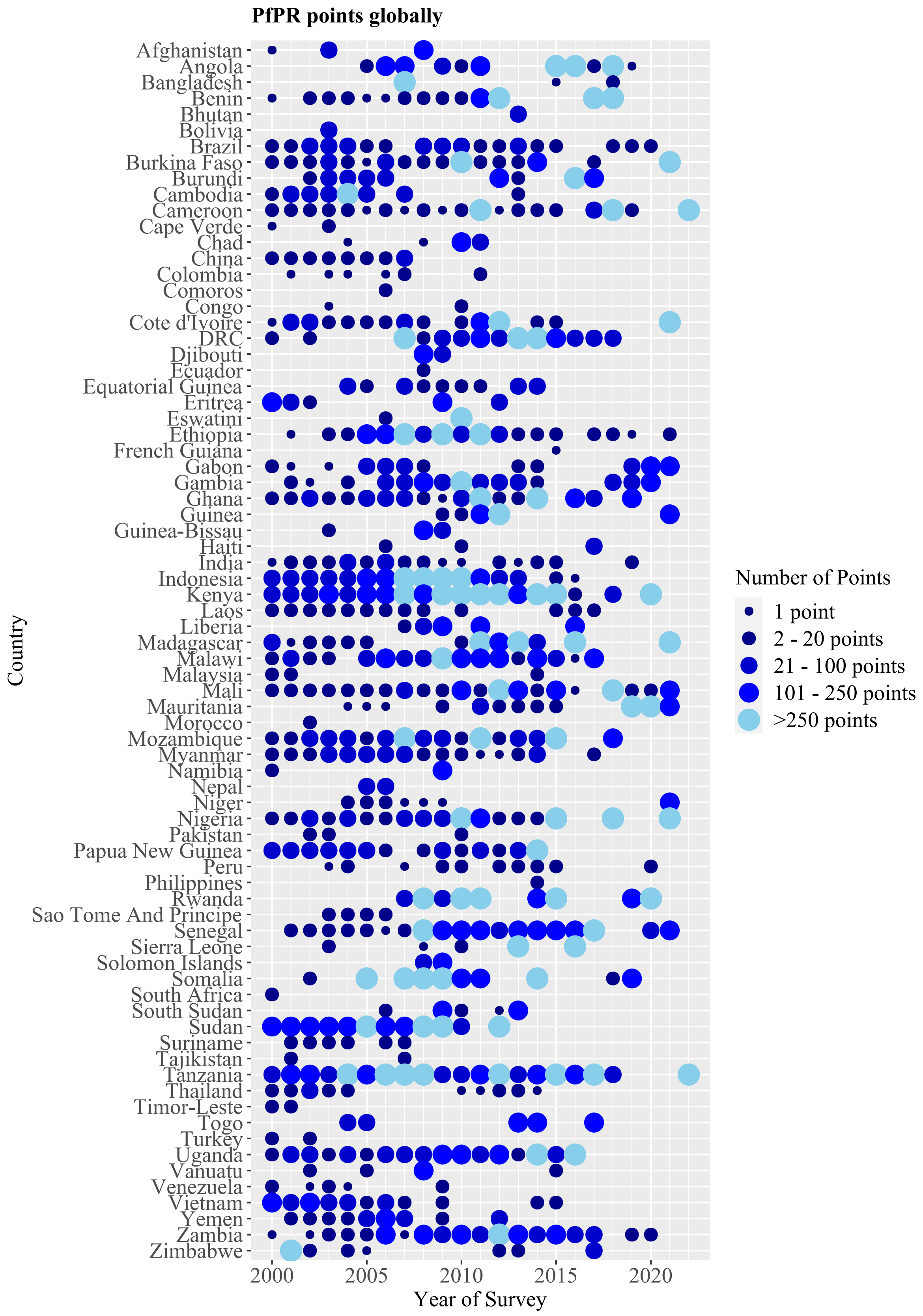

Figure S8. *Pf*PR global data summary.

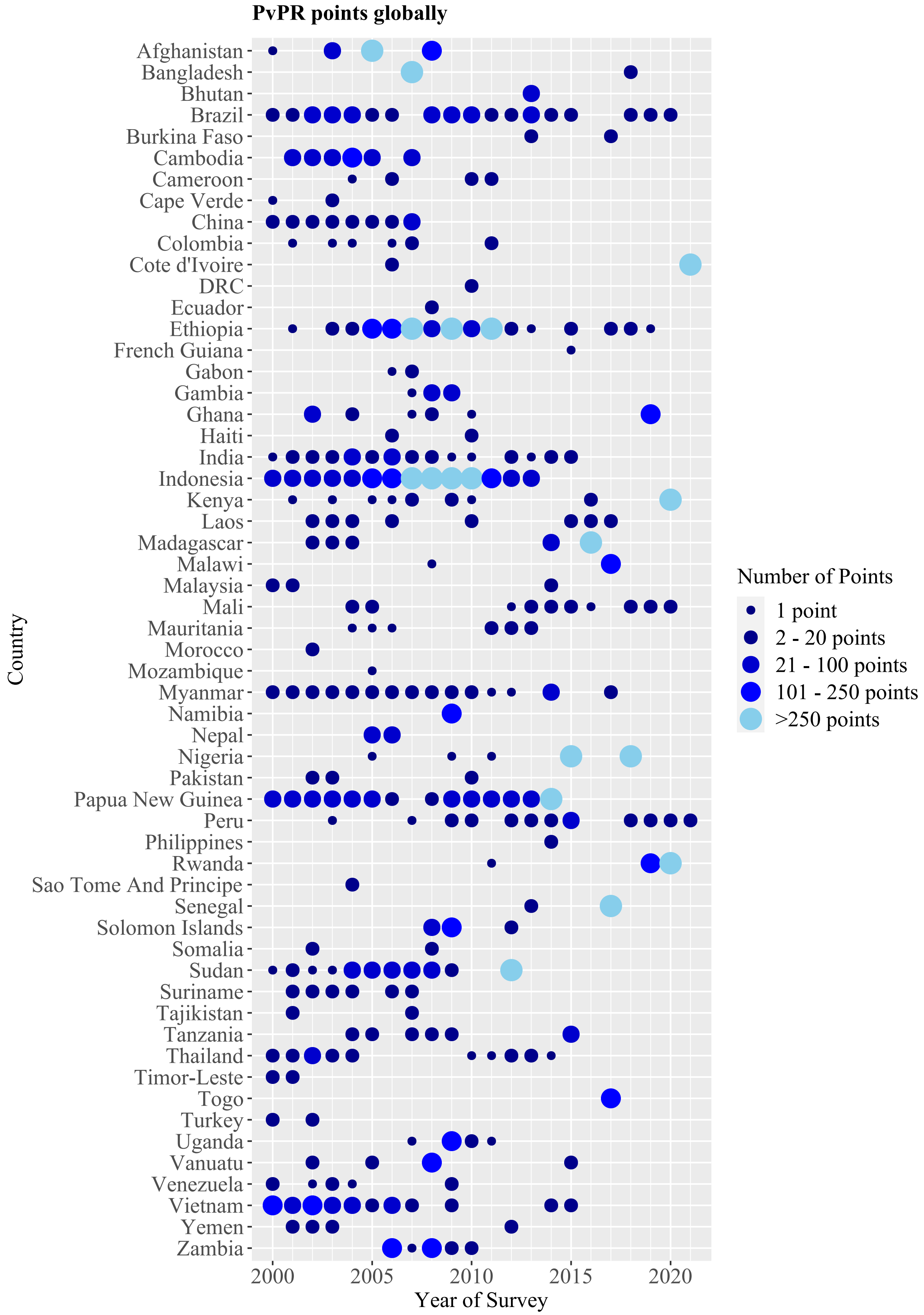

Figure S9. *Pv*PR global data summary.

2.1.4 Treatment-seeking data assembly

Data on treatment-seeking behavior in malaria endemic countries were gathered from DHS, Malaria Indicator Surveys (MIS), and Multiple Indicator Cluster Surveys (MICS) that were conducted from 1990. Treatment-seeking rates were determined from the number of children reported to have fever in the past two weeks for which treatment was sought. Response codes were manually classified into treatment at public points of care, such as government hospitals, clinics, and community health workers; and any treatment, which included all public treatment as well as any other locations where it is expected that people can obtain legitimate medication: private and non-governmental organisation (NGO) health facilities, privately owned pharmacies, and other retail facilities. Friends, family, and traditional and homeopathic healers were not considered as treatment. Data downloading, extraction, and processing were automated using a combination of tools, including Feature Manipulation Engine (FME), by Safe Software, OpenRefine, and custom data processing pipelines developed by MAP with Python and PostgreSQL. These tools were utilised to process data obtained from the DHS and MICS online platforms. All data were obtained from the DHS and MICS online platforms (https://dhsprogram.com/; https://mics.unicef.org/).[15, 16] The total number of children, fever cases and cases that sought treatment (public and any) were summarised using the sampling weights provided in the survey datasets to national level and at administrative level one for Kenya, Ethiopia, South Africa, Nigeria, Pakistan, Iran, Philippines, Brazil, India, China, Mexico, and Indonesia. Data were extracted from a total of 304 surveys from 91 countries, with 179 surveys in the Sub-Saharan African region, 42 from Latin America and Caribbean, 28 from Southeast Asia, East Asia, and Oceania, 20 from South Asia, 20 from North Africa and Middle East, and 15 from Central Asia.

2.1.5 Surveillance data collection

The suitability, availability and quality of PR and routine case reporting data, as well as detailed intervention coverage information, differs markedly between countries inside vs outside of Africa so separate modelling strategies were developed for countries inside Africa versus those outside (Figure S10, Table S2). The exceptions were Algeria, Egypt, Morocco, Comoros, Mauritius, Cape Verde, Sao Tome and Principe, Botswana, Namibia, Eritrea, Djibouti, South Africa, Zimbabwe, Gambia, Senegal, and Ethiopia. The modelling strategy used for most of Africa does not work well for island nations. The other countries have data availability that made modelling them with non-African countries more appropriate.

Malaria endemic countries outside of Africa tend to have less PR data than those within the continent, in part because prevalence is generally lower and thus PR becomes an inefficient way to measure malaria risk. In contrast, routine surveillance systems outside so Africa are generally more robust, so reports of malaria cases from health systems there are more reliable and provide some insight into the total malaria burden in the community. The protocol for collecting this surveillance data is detailed below.

Rules for data selection were developed to address conflicts arising between data sources for any given administrative unit in a country for a given year. Where there was consistency in the data reported between sources, it would be possible to apply a simple rule e.g. favoring the most recently published source. However, it is justifiable to assign a greater preference to some sorts of data over others regardless of the publication date. For example, two different sources might report the following conflicting data for a given administrative unit and year:

- Microscopy figures for explicitly stated indigenous species-specific cases.
- A figure for annual parasite incidence (API), with no indication on how this figure was calculated and whether or not it included only indigenous cases.

In this example, the microscopy-based figures offer estimates that are more robust for that administrative unit for that year, even if the overall API figures were published more recently.

In order to determine which figures should feed into the model in cases of conflict, data was assessed using up to three steps of processing with the following rules:

1. Allocate all data for a given admin unit-year combination to a “band” according to its perceived usefulness and reliability.
   - If the highest-ranking band for which there are data contains data from only one source, that data is used.
   - If the highest-ranking band for which there are data contains conflicting data from multiple sources, these data are processed as per step 2 – all data from lower-ranking bands are discarded.

2. Each set of figures from the same band is allocated points, according to the points table applicable to that band. This avoids giving points for irrelevant data – the points are for figures that are relevant for computing API with the data from that band.

- If a set of figures from one source has higher points than all other sources, then that set of figures is preferred.
- If two or more sources have equally high points, then those are processed as per step 3 and the other lower-scoring sources for that band are discarded.

3. For sets of figures from equally-scoring sources, the most recently published source is preferred. If the sources have the same publication date, one is chosen at random.

- We allocate all data for a given admin unit-year combination to a band according to its perceived usefulness and reliability. Table S2 details the variety of data reported ranked in descending order of perceived usefulness and reliability.
- A row in a higher band will always be preferred over a row in a lower band.
- When there are competing sources within a single band these are tie-broken according to points system below.

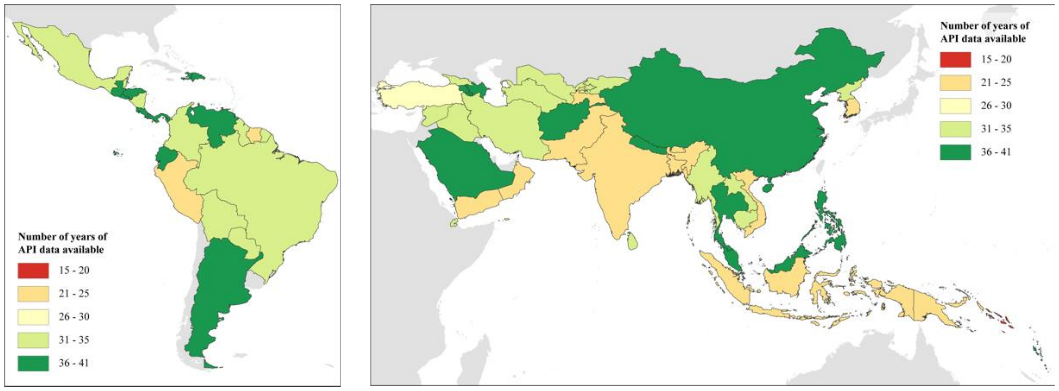

Figure S10. National-level surveillance data for years 2000-2022.

Table S2. Modelling approach used in malaria endemic countries and the years for which modelled burden estimates were produced. Note that *P. vivax* burden was not estimated in most Africa countries, consistent with the approach used by WHO for the World Malaria Report. NA, not applicable.

**Name**	**ISO3**	**Modelling Method**	***Pf* endemic years**	***Pv* endemic years**
Afghanistan	AFG	Surveillance	2000-2022	2000-2022
Angola	AGO	Cartographic	2000-2022	NA
Argentina	ARG	Surveillance	2000-2007	2000-2010
Armenia	ARM	Surveillance	2000-2004	2000-2005
Azerbaijan	AZE	Surveillance	2000-2008	2000-2012
Burundi	BDI	Cartographic	2000-2022	NA
Benin	BEN	Cartographic	2000-2022	NA
Burkina Faso	BFA	Cartographic	2000-2022	NA
Bangladesh	BGD	Surveillance	2000-2022	2000-2022
Belize	BLZ	Surveillance	2000-2018	2000-2018
Bolivia	BOL	Surveillance	2000-2022	2000-2022
Brazil	BRA	Surveillance	2000-2022	2000-2022
Bhutan	BTN	Surveillance	2000-2022	2000-2022
Botswana	BWA	Surveillance	2000-2022	NA

Central African Republic	CAF	Cartographic	2000-2022	NA
China	CHN	Surveillance	2000-2015	2000-2016
Cote d'Ivoire	CIV	Cartographic	2000-2022	NA
Cameroon	CMR	Cartographic	2000-2022	NA
Democratic Republic of the Congo	COD	Cartographic	2000-2022	NA
Congo	COG	Cartographic	2000-2022	NA
Colombia	COL	Surveillance	2000-2022	2000-2022
Comoros	COM	Surveillance	2000-2022	NA
Cape Verde	CPV	Surveillance	2000-2022	NA
Costa Rica	CRI	Surveillance	2000-2022	2000-2022
Djibouti	DJI	Surveillance	2000-2022	2000-2022
Dominican Republic	DOM	Surveillance	2000-2022	2000-2003
Algeria	DZA	Surveillance	2000-2013	NA
Ecuador	ECU	Surveillance	2000-2022	2000-2022
Eritrea	ERI	Surveillance	2000-2022	2000-2022
Ethiopia	ETH	Surveillance	2000-2022	2000-2022
Gabon	GAB	Cartographic	2000-2022	NA
Georgia	GEO	Surveillance	2000-2009	2000-2009
Ghana	GHA	Cartographic	2000-2022	NA
Guinea	GIN	Cartographic	2000-2022	NA
The Gambia	GMB	Surveillance	2000-2022	NA
Guinea-Bissau	GNB	Cartographic	2000-2022	NA
Equatorial Guinea	GNQ	Cartographic	2000-2022	NA
Guatemala	GTM	Surveillance	2000-2016	2000-2022
French Guiana	GUF	Surveillance	2000-2021	2000-2022
Guyana	GUY	Surveillance	2000-2022	2000-2022
Honduras	HND	Surveillance	2000-2022	2000-2022
Haiti	HTI	Surveillance	2000-2022	2000-2008
Indonesia	IDN	Surveillance	2000-2022	2000-2022
India	IND	Surveillance	2000-2022	2000-2022
Iran	IRN	Surveillance	2000-2020	2000-2022
Iraq	IRQ	Surveillance	2000-2008	2000-2008
Kenya	KEN	Cartographic	2000-2022	NA
Kyrgyzstan	KGZ	Surveillance	2000-2006	2000-2010
Cambodia	KHM	Surveillance	2000-2022	2000-2022
South Korea	KOR	Surveillance	2000-2012	2000-2022
Laos	LAO	Surveillance	2000-2022	2000-2022
Liberia	LBR	Cartographic	2000-2022	NA
Sri Lanka	LKA	Surveillance	2000-2012	2000-2012
Morocco	MAR	Surveillance	2000-2010	NA
Madagascar	MDG	Cartographic	2000-2022	2000-2022

Mexico	MEX	Surveillance	2000-2007	2000-2022
Mali	MLI	Cartographic	2000-2022	NA
Myanmar	MMR	Surveillance	2000-2022	2000-2022
Mozambique	MOZ	Cartographic	2000-2022	NA
Mauritania	MRT	Surveillance	2000-2022	NA
Malawi	MWI	Cartographic	2000-2022	NA
Malaysia	MYS	Surveillance	2000-2017	2000-2017
Namibia	NAM	Surveillance	2000-2022	NA
Niger	NER	Cartographic	2000-2022	NA
Nigeria	NGA	Cartographic	2000-2022	NA
Nicaragua	NIC	Surveillance	2000-2022	2000-2022
Nepal	NPL	Surveillance	2000-2022	2000-2022
Oman	OMN	Surveillance	2000-2022	2000-2022
Pakistan	PAK	Surveillance	2000-2022	2000-2022
Panama	PAN	Surveillance	2000-2022	2000-2022
Peru	PER	Surveillance	2000-2022	2000-2022
Philippines	PHL	Surveillance	2000-2022	2000-2022
Papua New Guinea	PNG	Surveillance	2000-2022	2000-2022
North Korea	PRK	Surveillance	NA	2000-2022
Paraguay	PRY	Surveillance	2000-2009	2000-2011
Rwanda	RWA	Cartographic	2000-2022	NA
Saudi Arabia	SAU	Surveillance	2000-2022	2000-2018
Sudan	SDN	Cartographic	2000-2022	2000-2022
Senegal	SEN	Surveillance	2000-2022	NA
Solomon Islands	SLB	Surveillance	2000-2022	2000-2022
Sierra Leone	SLE	Cartographic	2000-2022	NA
El Salvador	SLV	Surveillance	2000-2017	2000-2017
Somalia	SOM	Cartographic	2000-2022	2000-2022
South Sudan	SSD	Cartographic	2000-2022	NA
Sao Tome and Principe	STP	Surveillance	2000-2022	NA
Suriname	SUR	Surveillance	2000-2022	2000-2022
Eswatini	SWZ	Surveillance	2000-2022	NA
Syria	SYR	Surveillance	2000-2004	2000-2000
Chad	TCD	Cartographic	2000-2022	NA
Togo	TGO	Cartographic	2000-2022	NA
Thailand	THA	Surveillance	2000-2022	2000-2022
Tajikistan	TJK	Surveillance	2000-2013	2000-2014
Turkmenistan	TKM	Surveillance	NA	2000-2006
Timor-Leste	TLS	Surveillance	2000-2020	2000-2018
Turkey	TUR	Surveillance	2000-2007	2000-2009
Tanzania	TZA	Cartographic	2000-2022	NA

Uganda	UGA	Cartographic	2000-2022	NA
Uzbekistan	UZB	Surveillance	2000-2007	2000-2010
Venezuela	VEN	Surveillance	2000-2022	2000-2022
Vietnam	VNM	Surveillance	2000-2022	2000-2022
Vanuatu	VUT	Surveillance	2000-2020	2000-2022
Yemen	YEM	Surveillance	2000-2022	2000-2022
South Africa	ZAF	Surveillance	2000-2022	NA
Zambia	ZMB	Cartographic	2000-2022	NA
Zimbabwe	ZWE	Surveillance	2000-2022	NA

Table S3. Data bands.

Band	Band name	Band description
1	Has indigenous microscopy or rapid diagnostic test (RDT) confirmed cases by species	Indigenous microscopy or RDT confirmed cases by species is always the most desirable data.
2	Has non-explicitly indigenous microscopy or RDT by species but no imported cases	The fact the microscopy and RDT results are not explicitly stated to be indigenous is not an issue where there are no imported cases, so these are second only to explicitly indigenous results.
3	Has non-explicitly indigenous microscopy or RDT by species and species-specific imported cases	Microscopy or RDTs with species breakdowns but not explicitly stated to be indigenous are slightly less ideal if there are imported cases, even if these are by species, since it forces the assumption that these need to be taken off when this may vary by source reporting practice.
4	Has non-explicitly indigenous microscopy or RDT by species and has non-species-specific imported cases	Microscopy or RDT results with species breakdowns but not explicitly stated to be indigenous are less ideal if there are imported cases, especially where these are not by species, since a) it forces the assumption the imported cases should be subtracted from the total cases identified by the test results - i.e. we have to assume the test results include both indigenous and imported cases because it is not otherwise stated in the source b) these have to be taken off by species according to the ratio.
5	Has indigenous microscopy with no species breakdown	Microscopy or RDT results are better than confirmed cases without justification as to how they were confirmed. The best of these are explicitly indigenous, since there are no worries about imported cases
6	Has non-explicitly indigenous microscopy or RDT with no species breakdown but no imported cases	Microscopy or RDT results which are not broken down by species or explicitly stated to be indigenous where there are no imported cases to take off anyway are the next best results
7	Has non-explicitly indigenous microscopy or RDT with no species breakdown and imported cases	Microscopy or RDT results which are not broken down by species and are not explicitly stated to be indigenous where there are imported cases to remove, whether these imported cases are species specific.
8	Has confirmed cases without diagnostic details by species and no imported cases	Confirmed cases by species are preferred less than data with microscopy or RDT, because their confirmation method is unspecified. Those without imported cases to take off are preferred.
9	Has unjustified confirmed cases by species and imported cases by species	Where there are imported cases to take off confirmed cases by species, it is better to know the species breakdown of the imported cases than to have to compute an estimate.
10	Has unjustified confirmed cases by species and imported cases not broken down by species	Here there are confirmed cases by species, but the imported cases must be taken off based on the proportion of *Pf:Pv*:Other as given in the report if provided, or by a national estimate otherwise.
11	Has unjustified confirmed cases not broken down by species and no imported cases	Confirmed cases without justification or species breakdown must be reallocated by species; there are no imported cases to take off, though, which is preferred.

12	Has unjustified confirmed cases not broken down by species and imported cases	Confirmed cases without justification or species breakdown must be reallocated by species, and the imported cases taken off (and reallocated by species where these are not reported by species).
13	Has total cases explicitly stated to be local population	Where the only cases are unconfirmed, those explicitly stated to come from the local population take precedence.
14	Has explicit unconfirmed cases not stated to be local, or total cases not stated to be local	Where the only cases are unconfirmed, and this is not explicitly stated to come from the local population.
15	Has reported API	The lowest band is where only API is reported and must be transformed back into cases via the administrative unit population taken from GBD (and hence UN) figures.

2.2 Methods

2.2.1 Prevalence to incidence conversion (Cartographic approach)

In several instances throughout the analyses, we need to convert data from prevalence (*P. falciparum* parasite rate in ages 2–10, in the interval [0, 1]) to incidence (per person per year, in the interval [0, ∞). To do this we use a model that was published previously.[17] This involves applying an emulator approach to three *P. falciparum* microsimulation models – calibrated to a standardized data set – to obtain an ensemble model for the *Pf*PR-incidence relationship. Three models constitute the ensemble: EMOD, OpenMalaria, and malariasimulation.[18-20] The duration of infection within the prevalence to incidence model is approximately 200 days, a value derived from the fixed distributions used in each constituent model. For OpenMalaria this is drawn from a log Normal distribution of the mean (i.e., exp of the log mean) of 169 days.[21] For EMOD, the duration of infection is drawn from random sampling of a similar average duration.[22] And for the malariasimulation model, the duration of infection is drawn from an exponential distribution with fixed mean of 200 days.[20]

While the approach itself addresses model uncertainty, random effect terms in the model account for uncertainty due to the limited and noisy data by allowing for local, data-driven variations in the relationship between prevalence and incidence. After fitting, this model defines a function

$$\text{Prev2Inc}: f(P) = 2.616P - 3.596P^2 + 1.594P^3.$$

We call this function the prevalence-incidence relationship. By applying this function, we do not propagate the uncertainty in the relationship itself; however, by doing so at the realisation level, we propagate uncertainty from the *Pf*PR model. The rationale for this decision is to make the conversion computationally tractable over many pixels and realisations.

We also use the inverse of this model to calculate national-level *Pf*PR from national incidence estimates from time-series modelling (see section "Outside of Africa: time-series models"). However, due to the non-linear nature of the relationship, converting national-level incidence to prevalence does not give the same answer as converting pixel incidence to prevalence and taking the population-weighted mean. The values calculated from national-level incidence are therefore only used as covariates in CODEm (see section 3.2.2) models and not as results. Instead, final aggregate prevalence values are calculated as the population weighted mean of pixel prevalence.

While the prevalence-incidence relationship is a function over all values of prevalence ([0, 1]), the simple inverse of the equation is neither a function nor defined over all possible values of incidence. Therefore, for the reverse relationship, we cap incidence at the maximum value given by the prevalence-incidence relationship,

$$P_{\max} = \arg\max_{P} \text{Prev2Inc}(P).$$

This corresponds to a maximum prevalence $P_{max} = 0{\cdot}616$ and a maximum incidence rate of $I_{max} = 0{\cdot}620$ per person per year for all age incidence.

This inverse relationship also has no simple analytical form, so is solved numerically in each instance.

The incidence-prevalence function is therefore

$$\text{Inc2Prev}: f(I) = \begin{cases} \text{Prev2Inc}^{-1}, & \text{if } 0 < I < I_{\max} \\ P_{\max}, & \text{if } I > I_{\max} \end{cases}$$

As with the prevalence-incidence function, we apply the incidence-prevalence function at the realisation level to propagate uncertainty from the time-series models. The cap on the incidence-prevalence function is inconsequential when used to create national prevalence estimates from routine surveillance data because surveillance countries never approach this limit when the full population (i.e., urban and rural) is considered. In contrast, when spatially disaggregating the prevalence estimates, capping enforces spatial smoothing of hotspots to reduce false precision and misinterpretation of results derived by downscaling spatially aggregate routine surveillance data.

2.2.2 Incidence to prevalence conversion (Surveillance approach)

In several instances throughout the analyses, we need to convert data from incidence (per person per year, in the interval [0, ∞]) to prevalence (*P. vivax* parasite rate in ages 1–99 [$Pv\text{PR}_{1\text{-}99}$], in the interval [0, 1]). To do this we fitted a Bayesian mixed-effects model as an update to Battle et al.[17] In addition to the matched prevalence-incidence point surveys,[23] we matched polygon API data with *Pv*PR point surveys. For each *Pv*PR survey we matched it with the API value of the lowest administrative level data available in the API database. Given that subnational API data is incomplete through time, we selected the temporally nearest data each time. No limit was placed on the number of years between the timing of the PR survey collection and the matched subnational routine data to maximise the number of pairs available for modelling the conversion. The data was very left skewed with respect to API. To constrain the model so that API = 0 goes to prevalence = 0, we transformed the API as follows: log(API + min / 2) - min(log(API + min / 2)).
The incidence-prevalence model is given by

$$\text{logit}(P) = \beta_0 + \beta_1 I_t + \beta_2 s + \beta_3 s I_t + u_0 + u_I I_t + \epsilon$$

where P is prevalence, I_t is the transformed incidence and ϵ is the binomial error term. The model was fitted using (integrated nested Laplace approximation) INLA within the "INLA" package in R.
In the model definition, β_0 denotes the intercept. Due to the logit transform, we can't remove the intercept to force the model through $P = I_t = 0$ so instead we put a strong prior on β_0. We set $\beta_0 \sim N(-10, 0.0001)$ which implies that when $I_t = 0$, $P = \text{logit}^{-1}(-10) = 4{\cdot}5\times10^{-5}$.
β_1 is the fixed effect for transformed incidence.
β_2 is the fixed effect for the categorical variable for "data source", s with $s = 1$ when the data comes from the polygon API data and $s = 0$ otherwise. This variable is important for accounting for some of the outlier values from the *Pv*PR matched with polygons data. The final conversions from incidence to prevalence, i.e. the predictions from the fitted model, are made with $s = 0$.
β_3 is the fixed effect for the interaction between data source and incidence. Data from the polygon API dataset get this as an additional slope for incidence while data from the matched data, and final predictions, get no additional slope.
β_1, β_2, and β_3 are given the INLA default vague priors.
u_0 is the random intercept for the relapse zones. It is given by

$$u_0 \sim N(0, {\sigma_0}^2)$$

where σ_0 is the standard deviation of the random effect. σ_0 is given a penalised complexity prior (low values of σ_0 are preferred) such that the probability that $\sigma_0 > 0{\cdot}001$ is $0{\cdot}001$. This is a strong prior as we do not want this intercept to vary much at all based on the same logic as the strong prior for the intercept.
u_I is the random slope for incidence given the relapse zone and comes from a distribution

$$u_I \sim N(0, {\sigma_I}^2)$$

σ_I is again given a penalised complexity prior such that the probability that $\sigma_I > 1$ is $0{\cdot}01$. This is a relatively soft prior which allowed a reasonable balance between allowing the regions to vary and ensuring that the estimates are robust against outliers. Sensitivity testing was conducted to check that the model was not too sensitive to this choice

of the prior. Based on the fitted model, we have separate incidence to prevalence conversion formulae for each of the relapse regions.

2.2.3 Africa prevalence model

The large assembly of geolocated PR surveys maintained by MAP was used in a Bayesian spatiotemporal geostatistical model to predict *Pf*PR for every pixel-year in sub-Saharan Africa, representing an update to earlier work.[13] The model took into account (i) PR survey participant age ranges and diagnostic type; (ii) coverage of ITNs, IRS and treatment with an effective antimalarial drug and how these metrics changed through time at each data and prediction location; (iii) environmental conditions at each data and prediction location (including density of vegetation, temperature, humidity, rainfall, elevation, proximity to populated areas). The outcome was a predicted space-time "cube" of *Pf*PR, standardised to the 2–10 age range, for each year 2000–2022.
The *Pf*PR cube was then converted into an equivalent cube of the predicted incidence rate of clinical malaria using the prevalence-incidence relationship. This cube was then stratified into three broad age bins (0–5; 5–14; >15) using age-splitting models fitted previously.[17]

2.2.3.1 Africa prevalence model validation

To evaluate model fit for the Africa prevalence model we used marginal predictive statistics and k-fold cross validation with 10 folds of randomly selected hold out groups. We computed the conditional predictive ordinate (CPO) value of each observation, given by

$$p(y_i|y_{-i}) = \int p(y_i|\theta)p(\theta|y_{-i})d\theta,$$

where y_i is a single observation, y_{-i} is all observations excluding observation i, and θ are the parameters. A larger value of $\log(CPO_i)$ indicates that the model assigns high probabilities to the observations (i.e., that the observation is highly likely given the data and model). The mean of the log of the CPO values for our model was -1·4; indicating generally good predictive performance. The 10-fold cross-validation results indicated there is no substantial difference between in-sample and out-of-sample predictive accuracy. The average RMSE over the 10 folds was 0·15 for in-sample predictions and 0·16 for out-of-sample predictions. This consistency between in and out-of-sample prediction accuracy suggests that the model is not overfitting to the data.

2.2.4 Treatment-seeking model

Estimates of treatment seeking were generated for use as an input in the effective treatment with an antimalarial drug model (AM). The AM model combined treatment seeking with modelled proportional drug use and modelled drug efficacy.[24] The rationale for this approach is to split all malaria cases into those that received effective treatment (i.e., sought care and effectively treated by the proscribed drug) from those that do not receive effective treatment due to failure to seek care or because the drug they received was ineffective (e.g., the parasite was resistant to it or the patient failed to adhere to the dosing instructions).

2.2.4.1 Model approach

Generalised additive mixed models (GAMMs) were applied to obtain estimates of treatment-seeking rates for malaria endemic countries for the years 2000-2022. To calculate the proportion of children under five with fever that sought treatment at any type of provider or at public/government points of care, two GAMMs were built using information from indicator variables obtained from the Institute for Health Metrics and Evaluation following covariate selection as described previously.[25] The GAMM for treatment-seeking at any type of facility included the health expenditure per capita (USD) and pregnant women that received prenatal care as covariates. Treatment-seeking at public facilities were predicted using the number of hospital beds per 1000 people, the proportion of women giving birth in a health facility, and urbanicity. To account for temporal and spatial autocorrelation, both models included the survey year as a non-linear effect and the GBD region. Each GAMM is run as a mixed effects model across countries and not separately for each country. To calculate uncertainty of predicted treatment-seeking estimates, the models were run 1000 times. Each run sampled from the range of the 95% confidence intervals (CIs) of the observed treatment-seeking rates and indicator variable values as described above. Further details on the

treatment seeking model methodology have been described previously.[25] A noteworthy source of potential bias within these estimates arises from survey respondent recall of care seeking in the household survey response dataset.

2.2.4.2 COVID adjustments

Morbidity and mortality estimates were adjusted in cartographic countries in years 2020-2022 to account for the impacts of the COVID-19 pandemic on treatment-seeking. The per-country impacts of those adjustments on *P. falciparum* case incidence and mortality rates are shown in Table S4. Adjustment values of 0.00 in Table S4 are indicative of either values of zero in the Pulse surveys or countries in which a national survey was conducted after the onset of the pandemic. For the latter, the new empirical data on treatment seeking for fever superseded the need to model these disruptions. In brief, the methodology estimated a range of possible healthcare seeking disruptions that occurring in each year of the pandemic from the opinions of national experts responding in the surveys. The surveys were associated with multiple quarters of years, and when combined, spanned most quarters of 2020-2022. If no data were present for a country in a survey, the previous survey estimate was recycled. A full description of the methodology is available in Dzianach et al. 2023.[26]

Table S4. County-specific impact of COVID adjustments relative to the "COVID free" counterfactual.

ISO3	Name	Incidence increase 2020 (%)	Incidence increase 2021 (%)	Incidence increase 2022 (%)	Death increase 2020 (%)	Death increase 2021 (%)	Death increase 2022 (%)
AGO	Angola	9·65	40·01	21·03	1·55	6·52	6·92
BEN	Benin	1·91	8·09	11·28	0·63	1·07	0·88
BFA	Burkina Faso	23·33	0·00	0·00	0·00	0·00	0·00
BDI	Burundi	32·74	46·01	46·40	3·99	5·44	5·66
CIV	Côte d'Ivoire	2·67	2·77	2·88	0·60	0·00	0·00
CMR	Cameroon	25·29	20·98	14·30	0·00	0·00	0·00
CAF	Central African Republic	1·27	1·44	1·44	0·00	0·00	0·00
TCD	Chad	4·91	1·13	1·12	1·38	0·00	0·64
COG	Congo	2·72	2·99	3·00	0·01	0·01	0·63
COD	Democratic Republic of the Congo	4·50	7·46	2·56	0·47	0·47	0·47
GNQ	Equatorial Guinea	13·37	18·66	18·71	1·39	1·35	2·42
GAB	Gabon	3·83	0·00	0·00	0·00	0·00	0·00
GHA	Ghana	5·24	5·86	5·88	0·87	0·00	1·22
GIN	Guinea	9·33	0·00	0·00	0·00	0·00	0·00
GNB	Guinea-Bissau	14·89	24·63	18·46	5·70	9·88	9·45
KEN	Kenya	16·39	25·87	19·38	1·62	3·90	4·03
LBR	Liberia	35·45	18·83	6·55	1·17	0·90	0·84
MDG	Madagascar	1·72	0·00	0·00	0·00	0·00	0·00
MWI	Malawi	19·30	15·74	5·44	2·16	1·29	1·30
MLI	Mali	18·48	0·00	0·00	0·00	0·00	0·00
MOZ	Mozambique	30·03	23·36	4·09	3·63	2·27	2·19
NER	Niger	4·35	0·00	0·00	0·00	0·00	0·00
NGA	Nigeria	2·86	3·26	3·44	0·76	0·67	0·00
RWA	Rwanda	2·68	0·00	0·00	0·97	1·02	0·00

SLE	Sierra Leone	33·43	26·34	4·62	2·52	2·02	1·95
SOM	Somalia	0·79	0·86	0·87	0·00	0·00	0·00
SSD	South Sudan	1·89	2·00	1·99	0·00	0·46	0·00
SDN	Sudan	8·63	11·76	24·38	1·81	2·97	2·92
TGO	Togo	2·61	3·06	3·07	0·50	0·57	0·00
UGA	Uganda	62·47	51·53	9·08	4·82	2·94	3·12
TZA	United Republic of Tanzania	6·42	6·63	6·51	0·00	0·00	0·00
ZMB	Zambia	26·21	21·85	29·99	2·81	1·38	1·95

2.2.5 API estimation

2.2.5.1 API formulae

Annual *Plasmodium falciparum* incidence per 1000 population per year (AFI) and annual *Plasmodium vivax* incidence per 1000 population per year (AVI), were calculated at the national level for all countries and at every available sub-national level for which record sets could be obtained. The formula used for calculating non-species-specific annual parasite incidence per 1000 population per year (API) for a given administrative unit and year is trivial:

$$\text{API} = 1000\frac{M}{\text{population}}$$

where M is the number of cases for that administrative unit and year. However, the data gathered only includes cases that have been captured by the healthcare reporting systems of the respective countries. For countries with poorly developed health management information systems (HMIS), this might represent an under-reporting of cases. To obtain an estimate closer to the true number of cases for a given area, the formulae published by Cibulskis et al.[27] were used (with some additional considerations noted for India in a subsequent section). This approach takes the number of cases to be the mean of higher and lower estimates that each use treatment-seeking and slide positivity rates to adjust the stated number of cases:

$$M = \frac{M_{\text{upper}} + M_{\text{lower}}}{2}$$

Where:

$$M_{\text{upper}} = \frac{C + sU}{rp}$$

$$M_{\text{lower}} = \frac{(C + sU)(1 - n)}{rp} = \frac{a(C + sU)}{rp}$$

And:

C - Reported number of confirmed malaria cases in a year.
U - Reported number of unconfirmed malaria cases in a year.
s - Slide positivity rate
r - Reporting completeness
p - The proportion of the population with fever that seeks treatment from health facilities covered by the public reporting system.
n - The proportion of the population with fever that does not seek treatment.
a - The proportion of the population with fever that seeks treatment from any health facility (public and private).
Regarding the variables p, n, and a, the occurrence of fever is taken as a proxy for malaria.[27]
The source data gathered seldom provided the data in a format corresponding directly with the variables in the above formulae. In many cases, interpretation of the data with a predefined set of rules was required to determine appropriate values. These rules are set out in subsequent sections.

2.2.5.2 Calculating the proportions of *P. falciparum* and *P. vivax* cases from raw data

The above equations relate to non-species-specific API calculations. In order to calculate AFI and AVI, these equations need to be used with figures specific to *P. falciparum* and *P. vivax*. Wherever possible, figures from the primary sources were used to calculate the proportion of *P. falciparum* and *P. vivax* cases.
However, in many cases, only non-species-specific figures were available. In some cases, the source provided a figure for the proportion of cases that were *P. falciparum*. Often, they did not, so national level figures for species breakdowns were acquired annually from the WHO's World Health Organization's World Malaria Reports.[18-31] These were used to derive the proportion of *P. falciparum* and *P. vivax* malaria in each country and allow the calculation of confirmed and unconfirmed cases of *P. falciparum* and *P. vivax.*
The figures in these sets of World Malaria Report annexes list the number of *P. falciparum*, *P. vivax*, and other malaria cases, from which a proportion can be calculated. There were two issues:

- The dates covered by the reports overlapped and so the most recent report containing data for a given country in a given year was taken: the 2013 report covered the years 1990-1999, the 2015 report covered the years 2000-2009, the 2022 report covered the years 2010-2021, and a pre-release of the 2023 report provided to MAP by the WHO covered the year 2022.
- No country had a complete set of species breakdown figures for the entire period.

In summary, if no species-specific case figures were available in the source, the *P. falciparum* and *P. vivax* cases were calculated by multiplying the total cases by the best available species-proportion figures as follows:

- An explicitly stated proportion/percentage in the source.
- If there was nothing in the source paper, the WHO national species proportions from the annexes of the World Malaria Reports for the year of the source were used.
- If the WHO did not have a national species proportion for the year of the source, the mean species proportion for all years for that country was used.

2.2.5.3 Reported testing regimes

The principle testing regimes reported in sub-national data sources were microscopy tests and rapid diagnostic tests (RDTs). Only one source (the 2007 national survey from the Democratic Republic of the Congo) reported polymerase chain reaction (PCR) figures as a subset of microscopy tests. Since there was a lack of additional PCR results from other sources, the PCR values were not used. The vast majority of reported tests were microscopy. In a minority of cases, different sources reported conflicting figures for a given admin unit/year combination via microscopy tests versus RDTs. In these cases, the values from the microscopy tests took precedence over the RDT results. However, for surveys collected in countries during or shortly after administration of seasonal malaria chemoprevention (SMC) RDT was used even if both metrics were collected to mitigate the effects of SMC on detectability of parasites via microscopy. However, the RDT values from the survey were adjusted using the RDT to microscopy adjustment to standardise these inputs to the cartographic model. This caveat is relevant for the Nigeria 2021 MIS the Burkina Faso 2021 DHS, Mali 2021 MIS, Niger 2021 MIS, and the Guinea 2021 MIS.

2.2.5.4 Reported number of confirmed malaria cases in a year (*C*)

Confirmed cases were identified from sources where available and fell into the following six categories, listed in descending order of perceived quality:

Table S5. Confirmed cases fell into the following categories, listed in descending order of perceived quality.

Category of data	Derivation of *P. falciparum* confirmed cases
Species-specific cases confirmed via a testing regime (microscopy, RDT, or a combination of both), with figures of the numbers of tests undertaken and species-specific results.	Confirmed cases were taken directly from the data provided.
Species-specific cases confirmed via a testing regime (microscopy, RDT, or a combination of both) but without providing figures of the total number of examinations.	Confirmed cases were taken directly from the data provided.

Non-species-specific cases confirmed via a testing regime (microscopy, RDT, or a combination of both), with figures of the numbers of tests undertaken and species-specific results.	If the proportion of species-specific cases is stated in the source, it was used to calculate the number of confirmed cases. If not, the national proportion of species-specific cases was used for the year of the source. If no national proportion was available for the year, the mean proportion for those years available was used.
Non-species-specific cases confirmed via a testing regime (microscopy, RDT, or a combination of both) but without providing the figures on the number of tests undertaken.	If the proportion of species-specific cases is stated in the source, it was used to calculate the number of confirmed cases. If not, the national proportion of species-specific cases was used for the year of the source. If no national proportion was available for the year, the mean proportion for those years available was used. Species-specific cases, without indicating a testing regime or providing raw figures.
Note that an indication of species implies that a test process must have occurred and so the cases are confirmed.	Confirmed cases were taken directly from the data provided.
Non-species-specific cases explicitly stated as confirmed, without indicating a testing regime or providing raw figures. Note the requirement for the figures to have been explicitly stated as confirmed. This is distinct from the data described in the next section, where malaria case figures were provided without indicating they were confirmed.	If the proportion of species-specific cases is stated in the source, it was used to calculate the number of confirmed cases. If not, the national proportion of species-specific cases was used. If no national proportion was available for the year, the mean proportion for those years available was used.

The number of confirmed cases was taken as follows:

- If there were just microscopy figures, these were used as the number of confirmed cases.
- If there were just RDT figures, these were used as the number of confirmed cases.
- If there was no indication of the method used, the figures stated as confirmed were taken as the number of confirmed cases.
- If there were microscopy and RDT figures, the total number of confirmed cases was taken as the sum of the microscopy and RDT figures.

The result of the above was a species-specific number of confirmed cases but consideration needed to be taken to exclude cases that were imported. This depended on the source:

- If the number of cases by microscopy / RDT / unstated method were explicitly stated as being indigenous, they were taken as the final figure for the number of confirmed cases.
- If the number of cases by microscopy / RDT / unstated method were not explicitly stated as being indigenous and the source paper included species-specific figures of imported cases, these imported cases were subtracted from the appropriate species-specific number of confirmed cases to give a final figure for the number of confirmed cases.
- If the number of cases by microscopy / RDT / unstated method were not explicitly stated as being indigenous and the source paper included non-species-specific figures of imported cases, these imported cases were divided into species-specific figures by applying the same proportion of species indicated by the test result.

2.2.5.5 Reported number of unconfirmed cases in a year (*U*)

Many sources provided figures for malaria cases without explicitly stating these were confirmed cases. These figures might be provided in addition to figures for confirmed cases (such as microscopy tests or simply explicitly stated confirmed figures) and were likely cases treated following a presumptive diagnosis based on symptoms. Case figures not explicitly stated to be confirmed were assumed to be unconfirmed. Where additional confirmed cases were provided by a source, these were subtracted from the total case figures to provide a calculated number of unconfirmed cases. Consideration was given to figures for imported cases:

- If the source included figures for confirmed cases, the imported cases were subtracted from those in accordance to the proportion of species in the confirmed cases.
- If the source figures did not indicate any confirmed cases, the imported cases were subtracted from the unconfirmed cases to provide a calculated figure for unconfirmed cases.

Once a figure for unconfirmed cases had been calculated, it was used to calculate an estimate of the number of unconfirmed cases that were *P. falciparum* or *P. vivax*. This was done using the proportions described earlier in this document.

2.2.5.6 Reporting completeness (*r*)

Reporting completeness represents the number of health facilities reporting relative to the number of reports expected. National estimates for reporting completeness are provided by the WHO and are considered to be representative for the country. These data consist of annual estimates the WHO receives from malaria endemic countries. The country-level reporting completeness estimates are derived by the number of facility level reports received divided by the number of reports expected (i.e., the number of healthcare facilities multiplied by the number of reporting periods per year).

Most countries in the data provided by WHO had reported values for between three and eight years, which were not necessarily from consecutive years. To fill in the gaps between years, a mean value of all available years was used. To deal with the missing data prior to the earliest year for which a figure was available, the earliest reporting completeness available was assigned to one of the following bands:

- > 80%
- 50%-80%
- < 50%

The values for the missing earlier years were then calculated using a linear interpolation back to the base of the reporting completeness band the figure fell into. For example, if the earliest year for which data was available was 2005 and this figure was 65% reporting completeness, the years 2000 to 2004 would be calculated by decreasing the reporting completeness by even steps from 65% to 50% for the years 2004 to 2000 (with each year 1·5% less than the previous one): The justification for this is the assumption that reporting completeness has improved over time. For the < 50% band, a floor of 10% reporting completeness was assumed.

2.2.5.7 Slide positivity rate (*s*)

Slide Positivity Rate is defined as the number of microscopy slides found to be positive for malaria divided by the total number of slides examined. For the purposes of calculating AFI and AVI, the SPR for *P. falciparum* (SPR*Pf*) was required (i.e. the number of slides positive for *P. falciparum* divided by the total number of microscopy examinations undertaken), and analogously, SPR *P. vivax* (SPR*Pv*). Where figures were available in the source for the total number of examinations undertaken and the resultant slide-positives for *P. falciparum* or *P. vivax*, these were used to calculate SPR*Pf* and SPR*Pv*. Where the number of examinations and slide-positivity figures were available but did not specify a species, SPR was calculated and then multiplied by the best available figure for the proportion of *P. falciparum* or *P. vivax*. If the source did not have slide examinations and slide-positivity figures available, national figures were used instead. The national figures were derived from the slide figures in the appendices of the WHO's 2013, 2015, and 2022 World Malaria Reports, as well as from a pre-release of the data from the 2023 World Malaria Report.[28-31] Because the data in these reports overlaps, the data in the most recent report took precedence over the earlier ones. No country reported slide figures for the entire period under research, so a mean value was calculated for each country based on the years for which there were figures. This mean value was then used for the missing years for the country.

2.2.5.8 Population figures

Most of the sub-national case data collected from Ministry of Health reports had associated population figures. However, while these population figures were collected, they were disregarded in favor of figures provided by GBD because of the latter's provenance to the UN. For the purposes of calculating AFI and AVI, the population-at-risk was used as the denominator, but all resulting estimates are based on total population for the associated administrative unit.

2.2.5.9 Treatment-seeking figures (*p, n, a*)

Treatment-seeking rates are estimated as the proportion of children under five with fever that were taken to treatment (see section "Treatment-seeking model"). These values were used as representative of the population as a whole.[25]

These figures provide an upper, lower, and mean figure for each of:

- The proportion of children under five with fever seeking treatment from health facilities covered by the public reporting system.
- The proportion of children under five with fever seeking treatment from any health facility (public and private).

To reflect the uncertainty in the reliability of the data sources collected, the following assignments were made to the AFI and AVI calculations to reflect -the widest possible range of estimates.

For the calculation of M_{upper}:

p was set to the lower estimate of the proportion of children under five with fever seeking treatment from health facilities covered by the public reporting system. The formula for the upper cases estimates adjusts for cases being missed due to persons with fever who did not seek treatment from the public healthcare system; both those who sought treatment from the private sector, and those who sought no treatment. Using the lower estimate for this proportion amplifies the estimated number of fevers omitted from the public sector reporting and, accordingly, the upper cases estimate by the largest amount. The upper cases estimate assumes the same slide positivity rate, *s*, among those who do and do not seek treatment.[20]

For the calculation of M_{lower}:

p was set to the upper estimate of the proportion of children under five with fever seeking treatment from health facilities covered by the public reporting system. *a* was set to the lower estimate of the proportion of children under five with fever seeking treatment from any health facility (public and private).

The formula for the lower cases estimate adjusts for cases missed by persons with fever who did not seek treatment from the public healthcare system. The upper estimate for public treatment-seeking is used, since this provides the most optimistic view of treatment-seeking from the public healthcare system, and therefore of the completeness of the reported figures from the public sector. This reduces the number of fevers to be added to those from the public sector, and therefore the lower cases estimate.

By the same logic, the lower estimate for any treatment-seeking is used since, in combination with the upper public treatment- seeking estimate, this minimises the number of persons with fever estimated to have sought treatment within the private sector. Multiplying the slide positivity rate (*s*) used to adjust the unconfirmed cases by the proportion of persons with fever who sought any treatment applies the assumption that only fever cases that sought treatment had malaria and those that did not seek treatment had no malaria (s = 0).

2.2.5.10 Special considerations for India

Many malaria cases in India are treated in the private sector and hence go unreported by the HMIS.[27] The figures for cases and microscopy examinations in the appendices of the WHO's World Malaria Reports are those reported in the public sector and are therefore not representative of the total case burden in the country. Furthermore, the World Malaria Report figures represent data from both passive case detection (PCD: i.e. people presenting at hospital with symptoms) and active case detection (ACD: i.e. public healthcare going and screening everyone in a village) (Cibulskis RE, WHO, personal communication 2017).

This means that although we can calculate a slide positivity rate (SPR) from the figures of slides positive/slides examined in the public sector (published in the World Malaria Reports), we have no way of knowing what the SPR is for ACD or PCD – it is just a combined figure. The SPR for PCD will be higher than that for ACD – people are detected by PCD when they go to a hospital because they are ill. The ACD rates are based on active screening of fevers in the community meaning the febrile denominator population is likely to be larger than the subset that may seek care.

The World Malaria Reports include figures for the high, low, and mean case estimates for countries for selected years, based on the formulae discussed in section 2.1.5.1. The inputs for these estimates are the cases and number of slides reported in the appendices and unpublished treatment-seeking estimates calculated from Measure DHS (http://www.dhsprogram.com/) survey results (Cibulskis RE, WHO, personal communication 2017).

However, because the World Malaria Report figures for India do not include the totals for the significant number of cases treated in the private care sector and it is impossible to determine an appropriate value for SPR because of the high levels of ACD in the public sector, a different approach is taken for India for the high, low, and mean estimates in the World Malaria Reports (Cibulskis RE, WHO, personal communication 2017). Estimates of the number of private sector cases and cases where no treatment was sought are calculated by scaling up the public cases figures by the ratio of private treatment-seeking rates to public treatment-seeking rates and non-treatment-seeking rates to public treatment-seeking rates respectively. Because all cases in the private sector are passively detected, the private figures have to be modified upwards again by a factor to reflect that the PCD SPR is unknown. This factor is also

applied to the non-treatment-seeking cases. The WHO does this by using unpublished figures from the private sector that are not available publicly (Cibulskis RE, WHO, personal communication 2017). In order to provide credible national case figures for India, we adopted the same approach as the WHO, taking the following steps:

- The low, high, and mean case estimates for India published in the 2022 World Malaria Report were taken as anchor points.[30] There are estimates for every year in the range 2000-2022.
- The public sector case figures from the World Malaria Reports and treatment-seeking estimates (see section "Treatment-seeking model") were used to estimate private sector cases and non-treatment-seeking cases for each of the above years.
- The private and non-treatment-seeking cases were then scaled up by adjusting the SPR applied to them: the SPR was increased until the sum of reported public sector cases, estimated private sector cases, and estimated non-treatment-seeking cases approximated the point estimates published in the 2022 World Malaria Report.[30] We could not use the actual SPR adjustments used by the WHO in the World Malaria Reports because those are unpublished.
- The final national figures used by MAP were therefore the sum of reported public sector cases, estimated private sector cases scaled up by a modified SPR, and estimated non-treatment-seeking cases scaled up by a modified SPR.

2.2.5.11 Special considerations for countries in Elimination Phase

Countries classified by the WHO as being in "Malaria Elimination Phase" (i.e. API < 0·001) had their data treated as follows for the years they were in elimination phase:

- The reporting completeness was taken as 1 (i.e. 100% of cases reported), regardless of the reporting completeness reported by sources.
- The treatment-seeking values were taken to be 1 (i.e. 100% of patients seek treatment via the public healthcare system), regardless of the modelled treatment-seeking rates.
- All reported case figures were treated as confirmed, regardless of whether the sources explicitly reported them as such.

Countries classified by the WHO as being in "Pre-Elimination Phase" (i.e. API < 0·005) received no additional treatment during the calculation of AFI and AVI.

2.2.5.12 Outlier removal

Outlier removal was performed by two processes. At national level, data were examined and removed if they were considered unreliable. Unreliability was inferred by large changes year to year within a country and comparisons to other published estimates. The table of national data exclusions is given in Table S6. A further outlier rule was applied to subnational data. Data were removed if they either had an AFI or AVI of > 600; or had an AFI or AVI of > 100 and a population at risk of less than 10% of its population. Indian subnational units were exempt from this rule. Additionally, this exclusion pertains only to areas modelled using the surveillance method (i.e., low burden). In practice this outlier exclusion, which was rarely used and only applied to low administrative levels (e.g., admin level 3), occurred when large case numbers were attributed to small administrative units with low populations. The most common interpretation for such instances was that a medical facility (at which cases were counted) was located within the admin unit, and this facility attracted cases from neighboring areas (thus inflating the apparent APIs). Because high-resolution data on treatment seeking and migration behavior did not exist, we were unable to estimate catchments areas beyond the administrative unit boundaries. Truncated probability distributions could have been used in the likelihood to mitigate this issue without excluding the data, but that approach would also have required an arbitrary threshold for truncation while also introducing computational issues in implementation. As a result, we were limited to this practical restriction.

Table S6. National level data exclusion years.

Country	Excluded year(s)
Afghanistan	1994
Bangladesh	< 2000
Belize	1990–1999

Bolivia	1990–1999
Botswana	< 1990
Brazil	< 1990
Bhutan	< 1998
Colombia	< 1990
Comoros	< 2004
Djibouti	< 1990
Algeria	< 2000
Eritrea	< 1990
French Guiana	< 1990
Eswatini	< 2000
Guyana	< 1990
Haiti	1993, 1999, 2002, 2003
Indonesia	< 2000
Iran	< 1990
Kyrgyzstan	< 1990
Cambodia	< 1990
South Korea	< 2000
Laos	< 1990
Sri Lanka	< 1990
Mexico	< 1990
Myanmar	< 1990
Namibia	< 2000
Nicaragua	< 1990
Oman	< 1991
Pakistan	< 2000
Peru	< 2000
Papua New Guinea	< 2000
Paraguay	< 1990
Rwanda	< 2000
Solomon Islands	< 2008
Sao Tome Principe	1982
Suriname	< 2000
Tajikistan	< 2000
Timor-Leste	1998
Vietnam	< 2000
Yemen	< 2001
South Africa	< 2000

2.2.5.13 Post-hoc masking

This step creates a raster cube that indicates where pixels should be zero. Any administrative unit, year pair with zero API is included in the mask (i.e. set to zero) unless:

- The zero is due to a population at risk value of zero.
- A child administrative unit (i.e. a smaller administrative unit fully contained by the large unit) has a non-zero API value.

Furthermore, if a unit is included in the mask and all later data in that unit is zero API or missing, the mask is extrapolated forward in time. This is particularly needed because many areas stop reporting malaria cases once they have reached elimination.
For example:
If Goa had a zero API in 2011 and 2014 and an API of 0·1 in 2015, then the algorithm would:

- Do nothing.

If Goa had a zero API in 2011 and 2014 and nothing for 2015, then the algorithm would:

- Do nothing until 2011.
- Add 2011 to the mask, since it is reported as zero cases.
- Add 2012-2015 to the mask, since the most recent previous value was a zero, and there are no future values.

If Goa had a zero API in 2011 and 2014 and a zero API for 2015, then the algorithm would:

- Do nothing until 2011.
- Add 2011 to the mask, since it is reported as zero cases.
- Add 2012-2014 to the mask, since the most recent previous value was a zero, and the next reported value in 2015 was zero.
- Add 2015 to the mask, since it is reported as zero cases.

Finally, all administrative unit year pairs that are included in the mask are combined with the environmental limits to create a raster cube mask.

2.2.6 Surveillance country time-series models

For estimating malaria incidence outside of Africa, we first fitted time-series models to national AFI and AVI data. These time-series estimates were then included as data in the subsequent disaggregation regression models.

2.2.6.1 National time-series

The basic model for a national time-series includes short term (ST) and long term (LT) moving average elements to capture short-range and long-range variation. In hierarchical Bayesian notation, the API for country *i* in year *j* is modelled as:

$$
\begin{aligned}
API_{i,j} &\sim \begin{cases} N(\mu_{i,j}, \sigma_{i,j}^2) \\ N(\mu_{i,j}, (0.001\mu_{i,j} + 0.001)^2) & \text{for fixed data.} \end{cases} \\
\log(API_{i,j}) &= \begin{cases} \text{offset}_i, & \text{for } j = 1, \\ \text{offset}_i + \sum_{l=1}^{p} \beta_l X_{l,i,j} + w_{i,j-1}^{(ST)} + w_{i,j-1}^{(LT)} & \text{for } j > 1. \end{cases} \\
\text{where } w_{i,j}^{(ST)} &= \frac{\sum_{k<j} \exp\left(-\frac{(j-k)^2}{0.5^2}\right) \epsilon_{i,k}^{(ST)}}{\sum_{k<j} \exp\left(-\frac{(j-k)^2}{0.5^2}\right)}, \\
w_{i,j}^{(LT)} &= \frac{\sum_{k<j} \exp\left(-\frac{(j-k)^2}{2.5^2}\right) \epsilon_{i,k}^{(LT)}}{\sum_{k<j} \exp\left(-\frac{(j-k)^2}{2.5^2}\right)}, \\
\epsilon_{i,j}^{(ST)} &\sim N(0, \tau_i^2 * \rho), \\
\epsilon_{i,j}^{(LT)} &\sim N(0, \tau_i^2 * (1-\rho)), \\
\text{logit}(\rho) &\sim \text{Uniform}(-\infty, \infty), \\
\log(\tau_i^2) &\sim N(\gamma_i, \zeta_i^2), \\
\gamma_i &\sim N(-4,1), \\
\log(\zeta_i^2) &\sim N(-4,1), \\
\text{offset}_i &\sim N(0,10), \\
\text{and } \beta_l &\sim N(0,1).
\end{aligned}
$$

Here, $\mu_{i,j}$ and $\sigma_{i,j}^2$ denote the API mean and standard deviation from the API data. The latter is calculated using the upper and lower bounds of the API estimates.

$\{X_{l,i,j}\}$ represents the lth covariate for country i in year j. The covariates considered in the surveillance model, all of which were acquired from GBD, are shown in Table S7.

Table S7. Covariates used in the national-level surveillance model.

Covariate	Covariate id
Antenatal Care (one visit) Coverage (proportion)	1
Antenatal Care (four visits) Coverage (proportion)	2
DTP3 Coverage (proportion) - Fraction of children born in a given country-year who have received three doses of DTP3	3
Hospital Beds (per 1000)	4
In-Facility Delivery (proportion)	5
LDI (I$ per capita) - Lag distributed income per capita (I$): gross domestic product per capita that has been smoothed over the preceding ten years	6
Measles Vaccine Coverage (proportion)	7
Skilled Birth Attendance (proportion)	8
GDP per capita base 2010	9
Urbanicity (proportion)	10
Education (years per capita)	11
Health Industry Workers (The proportion of the employed population ages 15-69 working in health and social work)	12
Log-transformed national-level estimates for total health expenditure per capita	13

The covariates are normalised before testing and only significant covariates with negative coefficients are used in the final model. The above model is used for countries with large amounts of data over the study period. For countries with many missing values, we borrow information from countries in the same region by setting the long-term moving average ($w^{(LT)}$) as a shared regional trend. GBD super regions, which are based on epidemiological similarity and geographic closeness, were used as regions. If there are conflicting trends within the GBD super regions (e.g. increasing versus decreasing), countries with similar trends were grouped and treated as separate regions. Note that the basic model presented above cannot predict zero API values. To account for zeros especially in low API settings, we introduce a Tobit factor. If $\log(API_{i,j}) < c$, where c is the smallest $\log(API)$ value corresponding to a non-zero API value for that country, we set the API estimate to zero. The models were defined with the R package Template Model Builder (TMB)[32] and optimised in R. Once a model has been fit, we generate 1000 realisations of the API time series using the posterior distributions of the estimated parameters. These enable us to create 95% CIs.

2.2.6.1.1 Special case: Malaysia *P. knowlesi*

While *Pf* and *Pv* constitute most of the malaria burden worldwide, there may be times other malaria species may play a significant role, especially in elimination settings. As such, in addition to AFI and AVI, we model a combined estimate of *P. knowlesi* and *P. falciparum* incidence in Malaysia. *P. knowlesi* case counts from 2013, supplied by WHO, are added to the *P. falciparum* case numbers. To obtain a combined API, we apply the same API formula above with the same adjustments used for *P. falciparum* alone (i.e., we assume the same treatment seeking rates and reporting completeness are comparable between species). This is particularly true in Malaysia where they have eliminated both *Pf* and *Pv*, but have ongoing malaria cases caused by *Pk*. We therefore model a combined estimate that includes *Pk* case numbers which further flows into our malaria mortality models.

2.2.6.2 Subnational time-series

The models for subnational time-series modelling in subnational units of Brazil, China, Indonesia, India, Philippines, Pakistan, Iran, Mexico, South Africa, and Ethiopia are based on the regional time-series model. Kenya and Nigeria use the cartographic approach and aggregate administrative level API via the pixels. Now, instead of countries in one region sharing a LT moving average, subnational units in one country share the LT moving average. The main difference between the national models and the subnational models is that for the latter, the modelled subnational (ADMIN1) counts need to add up to the national count where we have national API data. That is, for subnational unit i in year j, we require that:

$$
\begin{aligned}
Count_{national,j} &= \sum_i Count_{i,j} \\
\text{where } Count_{national,j} &= \frac{API_{national,j}}{1000} \times Population_{i,j}, \\
\text{and } Count_{i,j} &= \frac{API_{i,j}}{1000} \times Population_{i,j}.
\end{aligned}
$$

Model variants involve the Tobit factor and omitting unit-specific ST moving averages.
For units where we have sufficient years with both subnational and national data, we proportionally adjust the subnational API data so that the subnational counts add up to the national count so as to help the model convergence ("prop_adj" = "Yes").
The GBD covariates, used for the national time series (Table S7), are also available at the ADMIN1 level. Additional covariates used for the subnational model are shown in Table S8.

Table S8. Covariates used in the subnational-level surveillance model.

Covariate	Covariate id
Mean national modelled API.	14
Indicator for the time period after 2012.	15
Indicator for the time period after 2013.	16
Indicator for the time period before 2000.	17

As before, the covariates are normalised before testing and only significant covariates with negative coefficients are used in the final model. Note that we do not model subnational units without API data or those with only zero values. For these units, we set their API values to zero since we lack data to suggest otherwise ("zeros" = "Yes"). If the national trend aggregated from the subnational time series differs significantly from that suggested by the national model, we proportionally adjust the subnational model draws using the national model draws ("draws" = "Yes"). This approach can be seen as using the subnational models to model the changes in the subnational proportions instead. Alternatively, we considered using the means of the national API estimates from the national model as a covariate. Note that a sensible coefficient sign for this would be positive. The details for the final models for the national and subnational models can be found in Tables S9:12.

2.2.6.2.1 Special case: Brazil

The API data for Brazil ADMIN1 units have different features (steady decrease and sharp decrease) which are difficult to capture with one subnational model. Instead, we group the units according to these features and model these two groups separately.

2.2.6.2.2 Special case: India

The API data for Indian ADMIN1 units have different features (fluctuating, steady decrease, drops in different years) which are difficult to capture with one subnational model. Instead, we group the units according to these features and model these 'clusters' separately. Since the clusters also have different degree of API variability from year to year, we allow the bandwidth of the moving averages ("st_bw") to vary between clusters. Since the subnational API data have been adjusted proportionally against the national API data, we can aggregate the subnational API estimates (via counts) to get the final India national API estimates.
To get the India urban and rural time series we split the state level time series based on population densities. For each admin unit, the population densities of urban and rural portions are calculated from the gridded population raster and GBD shapefiles that define urban and rural boundaries. The population densities are then linked to incidence rate via an empirical relationship. These urban and rural incidence rates are subsequently scaled, taking population into account, such that they are consistent with incidence rate for the entire admin unit.

Table S9. National time-series model variant descriptions.

Model	Long-term trend	Short-term trend	Covariates	Tobit	Comments
standalone	Country-specific	Country-specific	No	No	Trends governed by moving averages of differing smoothness. (Original model since no significant covariates upon fitting.)
rt	Region-shared	Country-specific	Yes	No	LT trend acts as averaging between countries but favours those with more data; ST trend accounts for remaining variability in country.
simple_tobit_nc	Region-shared	Country-specific	No	Yes	Treat the log(API) as a latent process and set a cut-off based on the smallest positive value observed. Predictions under cut-off are set to zero
standalone_tobit	Country-specific	Country-specific	No	Yes	

Table S10. National *P. falciparum* time-series model variants by country. Covariate IDs refer to those listed in Tables S7 and S8. NA, not applicable.

Country	National region	Model	Covariate id
Bolivia	ALA	rt	6,7,11,13
Ecuador	ALA	rt	6,7,11,13
Paraguay	ALA	rt	6,7,11,13
Peru	ALA	rt	6,7,11,13
Brazil	AmLA	rt	6
Colombia	AmLA	rt	6
French Guiana	AmLA	rt	6
Guyana	AmLA	rt	6
Suriname	AmLA	rt	6
Belize	CCLA	rt	7,8
Costa Rica	CCLA	rt	7,8
Guatemala	CCLA	rt	7,8
Mexico	CCLA	rt	7,8
Nicaragua	CCLA	rt	7,8
Armenia	CEECA	simple_tobit_nc	NA
Azerbaijan	CEECA	simple_tobit_nc	NA
Georgia	CEECA	simple_tobit_nc	NA
Kyrgyzstan	CEECA	simple_tobit_nc	NA
Uzbekistan	CEECA	simple_tobit_nc	NA
China	EAsia	rt	6
North Korea	EAsia	rt	6
South Korea	EAsia	rt	6
Tajikistan	EAsia	rt	6
Djibouti	ESA 1	rt	6
Eritrea	ESA 1	rt	6
South Sudan	ESA 1	rt	6
Yemen	ESA 1	rt	6
Comoros	MCM	rt	1
Madagascar	MCM	rt	1
Mayotte	MCM	rt	1
Iran	NAME	rt	9, 10
Oman	NAME	rt	9, 10

Saudi Arabia	NAME	rt	9, 10
East Timor	Oceania	rt	5, 11
Vanuatu	Oceania	rt	5, 11
Bhutan	SA1	rt	12
Nepal	SA1	rt	12
India	SA2	rt	2
Pakistan	SA2	rt	2
Cambodia	SEAsia	rt	2, 13
Indonesia	SEAsia	rt	2, 13
Laos	SEAsia	rt	2, 13
Myanmar	SEAsia	rt	2, 13
Papua New Guinea	SEAsia	rt	2, 13
Solomon Islands	SEAsia	rt	2, 13
Vietnam	SEAsia	rt	2, 13
Botswana	SSA	rt	8
Namibia	SSA	rt	8
South Africa	SSA	rt	8
Swaziland	SSA	rt	8
Afghanistan	Standalone	standalone	NA
Algeria	Standalone	standalone	NA
Bangladesh	Standalone	standalone	NA
Cape Verde	Standalone	standalone	NA
Dominican Republic	Standalone	standalone	NA
El Salvador	Standalone	standalone	NA
Haiti	Standalone	standalone	NA
Honduras	Standalone	standalone	NA
Malaysia	Standalone	standalone	NA
Morocco	Standalone	standalone	NA
Philippines	Standalone	standalone	NA
Syria	Standalone	standalone	NA
Sri Lanka	Standalone	standalone	NA
Thailand	Standalone	standalone	NA
Turkmenistan	Standalone	standalone	NA
Venezuela	Standalone	standalone	NA
Argentina	Standalone Tobit	standalone_tobit	NA
Iraq	Standalone Tobit	standalone_tobit	NA
Panama	Standalone Tobit	standalone_tobit	NA
Turkey	Standalone Tobit	standalone_tobit	NA
Guinea	WSA 1	rt	6
Guinea-Bissau	WSA 1	rt	6
Liberia	WSA 1	rt	6
Sao Tome and Principe	WSA 1	rt	6
Sierra Leone	WSA 1	rt	6

Table S11. National *P. vivax* time-series model variants by country. NA, not applicable.

Country	National region	Model	Covariate id
Brazil	AmLA	rt	5,6,10,12
Colombia	AmLA	rt	5,6,10,12
Guyana	AmLA	rt	5,6,10,12
Peru	AmLA	rt	5,6,10,12
Suriname	AmLA	rt	5,6,10,12
Armenia	CEECA	rt	7, 12, 13
Azerbaijan	CEECA	rt	7, 12, 13
Georgia	CEECA	rt	7, 12, 13
Kyrgyzstan	CEECA	rt	7, 12, 13
Turkmenistan	CEECA	rt	7, 12, 13
Uzbekistan	CEECA	rt	7, 12, 13
China	EAsia	rt	1,9
South Korea	EAsia	rt	1,9

Tajikistan	EAsia	rt	1,9
Djibouti	ESA	rt	13
Eritrea	ESA	rt	13
Yemen	ESA	rt	13
Belize	LAC	rt	1,2,5,6,11,12,13
Bolivia	LAC	rt	1,2,5,6,11,12,13
French Guiana	LAC	rt	1,2,5,6,11,12,13
Guatemala	LAC	rt	1,2,5,6,11,12,13
Honduras	LAC	rt	1,2,5,6,11,12,13
Mexico	LAC	rt	1,2,5,6,11,12,13
Nicaragua	LAC	rt	1,2,5,6,11,12,13
Paraguay	LAC	rt	1,2,5,6,11,12,13
Comoros	MCM	nc	NA
Madagascar	MCM	nc	NA
Mayotte	MCM	nc	NA
Algeria	NAME1	rt	1
Morocco	NAME1	rt	1
Iran	NAME2	simple_tobit_nc	NA
Iraq	NAME2	simple_tobit_nc	NA
East Timor	Oceania	rt	1,5,6,9
Vanuatu	Oceania	rt	1,5,6,9
Bangladech	SA1	rt	1,3,4,5,6
Bhutan	SA1	rt	1,3,4,5,6
Nepal	SA1	rt	1,3,4,5,6
India	SA2	rt	2
Pakistan	SA2	rt	2
Malaysia	SEAsia1	rt	3,4
Sri Lanka	SEAsia1	rt	3,4
Thailand	SEAsia1	rt	3,4
Cambodia	SEAsia2	rt	1,6
Indonesia	SEAsia2	rt	1,6
Laos	SEAsia2	rt	1,6
Myanmar	SEAsia2	rt	1,6
Papua New Guinea	SEAsia2	rt	1,6
Solomon Islands	SEAsia2	rt	1,6
Vietnam	SEAsia2	rt	1,6
Botswana	SSA	rt	8
Namibia	SSA	rt	8
South Africa	SSA	rt	8
Swaziland	SSA	rt	8
Afghanistan	Standalone	standalone	NA
Costa Rica	Standalone	standalone	NA
Ecuador	Standalone	standalone	NA
El Salvador	Standalone	standalone	NA
Ethiopia	Standalone	standalone	NA
North Korea	Standalone	standalone	NA
Oman	Standalone	standalone	NA
Panama	Standalone	standalone	NA
Philippines	Standalone	standalone	NA
Turkey	Standalone	standalone	NA
Venezuela	Standalone	standalone	NA
Argentina	Standalone Tobit	standalone_tobit	NA
Dominican Republic	Standalone Tobit	standalone_tobit	NA

Haiti	Standalone Tobit	standalone_tobit	NA
Sao Tome and Principe	Standalone Tobit	standalone_tobit	NA
Saudi Arabia	Standalone Tobit	standalone_tobit	NA
South Africa	Standalone Tobit	standalone_tobit	NA
Syria	Standalone Tobit	standalone_tobit	NA

Table S12. Subnational time-series model variant descriptions.

**Model**	**Long-term trend**	**Short-term trend**	**Covariates**	**Tobit**	**Comments**
General non-tobit model (nt)	Country-shared	ADMIN1-specific	Yes	No	ADMIN1 units are modelled as a region like *rt* of the national models
nt_nrt_nc	No	ADMIN1-Specific	No	No	ADMIN1 units are modelled as individual
nt_nsubt	Country-shared	No	Yes	No	
nt_nc	Country-shared	ADMIN1-specific	No	No	
tobit	Country-shared	ADMIN1-specific	Yes	Yes	ADMIN1 units are given a latent log(API) process and cut-off (based on the smallest positive value) to be able to account for and predict zeros.
cluster_sep	No	ADMIN1-specific	Yes	No	This model is the same as nt_nrt_nc includes covariates and allows the bandwidth of the moving averages to be varied more easily.

Table S13. Subnational *P. falciparum* time-series model variants by country. NA, not applicable.

**Country**	**Subnational Units**	**Prop_adj**	**Zeros**	**Model**	**Covariate id**	**st_bw**	**Draws**
Brazil	Alagoas, Bahia, Ceara, Distrito Federal, Espirito Santo, Goias, Maranhao, Mato Grosso, Mato Grosso do Sul, Minas Gerais, Paraiba, Parana, Piaui, Rio Grande do Norte, Rio Grande do Sul, Rondonia, Santa Catarina, Sao Paulo, Tocantins	Yes	Yes	nt_nsubt	4,5,10,11,13	0·5	Yes
Brazil	Acre, Amapa, Amazonas, Para, Pernambuco, Rio de Janeiro, Roraima, Sergipe	Yes	Yes	nt_nsubt	1,4,12,13	0·5	Yes
China	All	Yes	Yes	tobit	6, 8	0·5	No
Ethiopia	All	No	No	nt	14	0·5	Yes
Indonesia	All	No	No	nt	1	0·5	Yes
India	Andhra Pradesh, Bihar, Chhattisgarh, Gujarat, Haryana, Jharkhand, Kerala, Manipur, Meghalaya, Mizoram,	No	No	cluster_sep	5	1·1	No

	Odisha, Telangana, Tripura						
India	Arunachal Pradesh, Assam, Madhya Pradesh, Maharashtra, Nagaland, Sikkim, West Bengal, The Six Minor Territories	No	No	cluster_sep	7, 15	1·2	No
India	Himachal Pradesh, Punjab, Uttarakhand, NCT of Delhi	No	No	cluster_sep	15	1·2	No
India	Jammu and Kashmir, Tamil Nadu, Uttar Pradesh, Rajasthan, Goa, Karnataka	No	No	cluster_sep	15	1·5	No
Iran	All	No	Yes	pf_nrt_nc	NA	0·5	Yes
Mexico	All	No	Yes	pf_nrt_nc	NA	0·5	Yes
Pakistan	All	Yes	No	pf_nc	NA	0·5	Yes
Philippines	All	No	Yes	pf	7, 10	0·5	Yes
South Africa	All	No	Yes	pf_tobit	1,2,3	0·5	Yes

Table S14. Subnational *P. vivax* time-series model variants by country. NA, not applicable.

Country	Subnational Units	Prop_adj	Zeros	Model	Covariate id	st_bw	Draws
Brazil	Alagoas, Bahia, Ceara, Distrito Federal, Espirito Santo, Goias, Maranhao, Mato Grosso, Mato Grosso do Sul, Minas Gerais, Paraiba, Parana, Piaui, Rio Grande do Norte, Rio Grande do Sul, Rondonia, Santa Catarina, Sao Paulo, Tocantins	Yes	No	nt_nsubt	4,5,12,13	0·5	Yes
Brazil	Acre, Amapa, Amazonas, Para, Pernambuco, Rio de Janeiro, Roraima, Sergipe	Yes	No	nt_nsubt	1,3,6	0·5	Yes
China	All	No	No	tobit	4,5,6	0·5	No
Ethiopia	All	No	No	nt_nc	NA	0·5	Yes
Indonesia	All	No	No	nt_nc	NA	0·5	Yes
India	Bihar, Himachal Pradesh, Jammu and Kashmir, Kerala, Maharashtra, Nagaland, The Six Minor Territories, Uttar Pradesh, Uttarakhand	No	No	cluster_sep	13	1·1	No
India	Arunachal Pradesh Karnataka, Madhya Pradesh, Tamil Nadu	No	No	cluster_sep	12	1·4	No
India	Assam, Chhattisgarh, Odisha, Meghalaya, Mizoram, Tripura	No	No	cluster_sep	5,15	1·4	No
India	Andhra Pradesh, NCT of Delhi, Goa, Telangana	No	No	cluster_sep	13,15	1	No
India	Gujarat, Haryana, Jharkhand, Manipur, Punjab, Rajasthan, Sikkim, West Bengal	No	No	cluster_sep	2,17	1·4	No
Iran	All	No	Yes	nt_nc	NA	0·5	Yes
Mexico	All	Yes	Yes	nt_nc	NA	0·5	Yes
Pakistan	All	Yes	Yes	nt_nrt_nc	NA	0·5	Yes
Philippines	All	No	Yes	nt_nc	NA	0·5	Yes
South Africa	All	No	No	tobit	1,11	0·5	Yes

2.2.6.3 Surveillance model validation

To evaluate the performance of the surveillance model we performed a k-fold cross validation with 10 folds. We randomly split the data into 10 equally sized partitions, rotating through the folds using 9 folds for training and one hold out set for testing each time. For each iteration we made predictions for both the training and test data. We obtained out-of-sample predictions for all observations by collating the out-of-sample predictions for each iteration.

The coverage is the probability of a confidence region including the observation and was 93% for the out-of-sample predictions for both parasites. This is close to the expected value of 95%. The measured incidence for *P. falciparum* had a mean of 0·020 but varied widely by country and year from 0·0 to 1·43. Even proportionally small errors on large incidence measurements can dominate the absolute error measures. Due to the wide range of observed values, we measure the fit quality in linear-predictor space (*i.e.*, taking the log of the values). For N observations, $\hat{y}_i$, and out-of-sample predictions, y_i, we evaluated the mean average error (MAE) and root mean square error (RMSE) given by

$$MAE = \frac{1}{N}\sum|\log(y_i) - \log(\hat{y}_i)|, \quad RMSE = \sqrt{\frac{1}{N}\sum(\log(y_i) - \log(\hat{y}_i))^2} .$$

We added a small displacement of 0·0001 to the observations when computing the log. The in-sample MAE and RMSE respectively were 0·04 and 0·17 for *P. falciparum*, and 0·03 and 0·15 for *P. vivax*. The out-of-sample MAE and RMSE respectively were 0·95 and 1·61 for *P. falciparum*, and 1·21 and 2·33 for *P. vivax*. These numbers indicate that the predictions for the out-of-sample observations are of comparable in scale to the observations. The out-of-sample predictive accuracy was worse than the in-sample, which could indicate overfitting; however, the correlations between observation and out-of-sample predictions were 0.29 and 0.56 for *P. falciparum* and *P. vivax,* indicating that out-of-sample predictions were generally well correlated with the corresponding hold-out data. The fact that the correlation for *P. falciparum* was comparatively lower is an artifact of heteroskedasticity. The weighted R-squared value for *P. falciparum* (i.e., the R-squared value for a linear regression using the inverse of the prediction variances as regression weights) was 0·5.

2.2.7 Surveillance country disaggregation regression

2.2.7.1 Machine learning with PR points

To incorporate information from PR surveys, we fitted several machine learning models to PR survey data and environmental covariates. Models were fitted using the 'caret' package.[33] We then predicted these models globally. The new predicted surfaces were then used as covariates in later models. We used all data in the MAP PR database as described above. The prevalence proportion was converted to incidence rate using the prevalence-incidence model.

2.2.7.1.1 Model validation

Five-fold cross validation was used to select hyperparameters and measure model accuracy. For each model, grid search was used to create candidate hyperparameter sets. That is, for each hyperparameter a number of values were chosen and then models were fitted with all possible combinations of these values. Root mean squared error was used as the metric of model accuracy.

2.2.7.1.2 Models and hyperparameters

We fitted a number of regression models to predict incidence from the set of covariates. The models fitted were elastic nets,[34] random forests,[35] k-nearest neighbor, robust linear models,[36] gradient boosted tree,[37] and neural networks.[36] As the predictions from these models are to be used as covariates by later models, models with uncorrelated predictions are the most useful. These models were selected as they cover several underlying model structures (tree-based methods, nearest neighbor methods, linear models, neural networks) which gives them the best chance at making uncorrelated predictions. Finally, we selected five models by fitting an elastic net model using prevalence as the response variable and out-of-sample predictions from the models as covariates. The alpha parameter (fraction of LASSO penalty vs ridge penalty) was set to 0·05. As the ridge penalty is strong, this will penalise correlated covariates, while including the LASSO penalty allows the model to force coefficients to exactly zero and therefore perform covariate selection.

2.2.7.2 Disaggregation regression

2.2.7.2.1 Data

The response data is malaria incidence rate (per person per year) for an associated spatial polygon. The data comes from two sources.
The first set of data comes from time-series models fitted previously. These time-series models are fitted to data at the national level and to the ADMIN1 level for a select few countries (Brazil, China, India, Indonesia, Iran, Mexico, Philippines, Pakistan, Ethiopia, Kenya, Nigeria, and South Africa). In the case of India, each state is split into two, a rural polygon and an urban polygon. These are the results published as part of the GBD2021 study.[38, 39] Therefore, the results here are required to exactly match these results. As the data are from a time-series model, they are complete and have associated uncertainty estimates. In the subsequent disaggregation modelling, these datapoints are considered equally whether they are at the national or subnational level and are referred to collectively as "ADMIN0".
The second set of data are any additional subnational incidence rate data (sub-ADMIN1 for Brazil, China, India, Indonesia, Iran, Mexico, Philippines, Pakistan, Ethiopia, Kenya, Nigeria, and South Africa). These data have upper and lower bounds based on M_{upper} and M_{lower} as described in section 2.2.5.1 ("API formulae"). The mean of the upper and lower bound is used as the point estimate of incidence. The polygons associated with these data range from ADMIN1 to ADMIN3 levels. They form a hierarchy with each polygon is considered a child of the larger polygon within which it is located.

2.2.7.2.2 Model definition

We define a multi-level, disaggregation regression model inspired by Sturrock et al.[40] This model uses polygon-level incidence rate data with pixel-level covariates and a spatial random field. Throughout, we index polygon-level variables with j and pixel level variables with i. We start by defining the linear predictor which contains an intercept (β_0), covariates ($\boldsymbol{\beta}\mathbf{X}$), a spatial random field ($\mathrm{GP}(s_i)$) and an independent and identically distributed (iid) random effect (u_j)

$$\eta_i = \beta_0 + \boldsymbol{\beta}\mathbf{X}_i + \mathrm{GP}(s_i) + u_j$$

Here, $\boldsymbol{\beta}$ is a vector of M regression slope parameters and $\mathbf{X}_i$ is a vector of M covariate values at pixel i. The iid random effects for subnational polygons are grouped by the ADMIN0 polygon each unit falls within. The one exception to this is India as dividing the states into rural and urban means polygons no longer fit into a clear hierarchy. Therefore, in India there is one group per state and each group will contain two ADMIN0 polygons.

The spatial random effect is a continuous random field. For tractability the random field is implemented as a Gaussian Markov Random Field approximation to the full continuous field using the SPDE approach.[41] We used the Matern covariance function (as required by this approximation) and created triangular meshes for each separately modelled region.
We then define the link-function between the linear predictor and I_i. To consistently link incidence and prevalence, we choose a link function that passes through prevalence. Furthermore, as this model is only being applied in lower burden areas, we constrain the model to be defined between an incidence of 0 and I_{max} = 0·612 as 0·612 is the maximum value given by the prevalence incidence relationship. This corresponds to a maximum prevalence P_{max} = 0·616. This constraint prevents many stability issues caused by the non-monotonic shape of Prev2Inc.
We have pixel-level prevalence P_i given by

$$P_i = P_{\max}\mathrm{logit}(\eta_i)^2$$

where the exponent simply scales the linear predictor to help small incidences to be distinguishable. From here we have

$$I_i = \mathrm{Prev2Inc}(P_i)$$

to transform to the incidence scale. We then define the relationship between polygon-level incidence, I_j, and pixel-level incidence, I_i

$$I_j = \frac{\sum_{i \in j} I_i \times \text{pop}_i}{\sum_{i \in j} \text{pop}_i}$$

with pop_i being the pixel-level population and the summations going over all pixels in polygon j.

2.2.7.2.3 Likelihood definition

We use a pseudo-likelihood that captures the uncertainty in incidence values. For each incidence value we have an upper bound and lower bound, I_j^U and I_j^L. For ADMIN0 data these bounds are given by the 95% uncertainty intervals from the time-series models. For subnational data the bounds are given by incidence rates calculated using M_{upper} and M_{lower} as described in section "AFI formulae". We define the likelihood for each incidence value as

$$\log(I_j) \sim \text{Norm}(\mu_j, \sigma_j^2)$$
$$\mu_j = \log\left(\frac{I_j^U + I_j^L}{2}\right)$$
$$\sigma_j^2 = \frac{\log(I_j^U) + \log(I_j^L)}{2 \times 1.96}$$

For data with $I_j^U = I_j^L$ we assign σ_j^2 as the smallest non-zero value of σ_j^2.

2.2.7.2.4 Priors

We set the priors on the fixed effects, β_0 and $\boldsymbol{\beta}$ as

$$\beta_0 \sim \text{Norm}(-4,2)$$

and

$$\beta_{m \in 1:M} \sim \text{Norm}(0,0.5).$$

The weakly informative, zero centered priors on the regression parameters help to regularise the model. This is particularly important due to the large number of covariates being used relative to the number of data points being included in each modelled region. To select this value, we considered that one covariate alone should not be able to explain the full range of observed malaria incidence rates. The 95% quantiles of this prior, coupled with approximately normal covariates scaled to have a standard deviation of one, would allow a single covariate to explain a little less than the full range of observed malaria incidence.

The spatial random field has a Gaussian process prior with hyperpriors

$$\log(\kappa) \sim \text{Norm}(-3,0.3)$$

and

$$\log(\tau) \sim \text{Norm}(0,1).$$

Finally, the iid random effect has prior

$$u_j \sim \text{Norm}(0,1).$$

Again, the zero-centered, weakly-informative prior helps to prevent overfitting.

2.2.7.2.5 Weighting by admin level

The polygon data has a hierarchical structure with ADMIN0 polygons and nested, subnational ADMIN1 and ADMIN2 polygons. The data within these levels is imbalanced and varies from country to country. One country

may have an ADMIN0 data value and thousands of subnational data values, while another may only have data for ADMIN0.
To address this imbalance, we weight the data by admin level. We weight ADMIN0 data by 5, ADMIN1 by 1, ADMIN2 by 0·01 and ADMIN3 by 0·005. In the South America region, due to the very large number of ADMIN2 datapoints, we instead weight ADMIN2 and ADMIN3 by 0·001. Given that only around 10 countries are included in each regional analysis, we could not weight ADMIN0 data by 1 and down weight all other data as this would give unreasonable weight to the priors. It was decided that ADMIN1 data are reliable enough to be weighted by 1, with ADMIN0 data adjusted accordingly to maintain balance. This ensured that the combined weight of ADMIN1 data for a country does not significantly exceed the weight of the ADMIN0 unit. Likewise, the down weighting values for ADMIN2 and ADMIN3 units are calculated to ensure that the combined weight of these data does not surpass the higher-level, more reliable data they fall under in the hierarchy.

2.2.7.2.6 Computational model fitting

To find the maximum a posteriori estimate for the model, we optimise a vector θ of all parameters and hyperparameters such that we find the values that minimise $-\log(p(I|\theta)p(\theta))$. As this is proportional to the true posterior, the parameter values that minimize this expression are also the mode of the posterior. The model was defined with the R package Template Model Builder (TMB)[32] and optimised in R.

2.2.7.2.7 Temporal Interpolation

To allow the malaria surface to change through time we fit two models per region (2005 and 2015). After fitting, both these models are predicted globally and malaria surfaces for other year are calculated by linearly interpolating between them. However, it is only the underlying surface that it interpolated linearly as the results from the time-series models are raked over these surfaces. For the covariates other than precipitation, night-time lights, elevation, accessibility and PET, the appropriate year data are used for both model fitting and prediction. We then linearly interpolate between the two models. For an incidence value at pixel *i* and year *t* we have

$$I_{i,t} = w_{2015} I_{i,2015} + (1 - w_{2015}) I_{i,2005}$$

where w_{2015} is the 2015 weight and is given by

$$w_{2015} = \begin{cases} 0, & \text{if } t \leq 2005 \\ 1, & \text{if } t \geq 2015 \\ \dfrac{t - 2005}{2015 - 2005}, & \text{if } 2005 < t < 2015 \end{cases}$$

2.2.7.2.8 Masking and population

After the models were predicted and interpolated, so that we have a full space-time cube of incidence, we use a mask (see section “Post-hoc masking”) to ensure that areas known to be malaria-free are predicted as such. This is particularly needed because the model cannot predict a true zero. For all region-year pair in the post-hoc mask table, we set all incidence and prevalence pixels in the appropriate polygons to zero. Pixels with zero population-at-risk are also set to incidence and prevalence of zero as these metrics are undefined with a denominator of zero.

2.2.7.2.9 Bootstrap uncertainty

After finding the maximum a posteriori estimate of the posterior we need a method to draw 100 independent samples from the posterior. However, the Laplace approximations calculated by Template Model Builder failed to find estimates for a multivariate normal approximation to the posterior. We also attempted to use Hamiltonion Markov Chains (HMC) to draw samples from the posterior, but the chains mixed very poorly, and given the slow evaluation of $-\log(p(I|\theta)p(\theta))$, the expected runtime to get 100 independent samples was prohibitively high.

Similarly, attempts at importance sampling and relative importance sample failed. Instead, we used bootstrapping to obtain 100 samples that characterise the uncertainty in the model. In designing the bootstrap resampling scheme, we account for both the fact that the ADMIN0 data raked over predicted maps come from a separate time-series model and the hierarchical nature of the polygon data.
For each bootstrap resample, the following scheme was used:

1. For each ADMIN0 unit, sample the posterior of the time-series models.
2. Select polygons with probability 0·05.
3. Remove all data that are descendants of selected polygons (i.e. admin units below the selected polygons).
4. Remove the selected polygons themselves unless the polygon is ADMIN0.
5. Fit the model to the remaining data and rake the surface back to the resampled ADMIN0 values.

Therefore, for each bootstrap, there is always complete ADMIN0 data. This is required as the model is raked to these values in each case. Given that the bootstrap is characterising the uncertainty in the spatial pattern of incidence within ADMIN0 units, this is reasonable. This sampling scheme also means that the amount of missing data per bootstrap iteration is highly variable: some ADMIN0 units have thousands of descendant polygons while some have none. Furthermore, as each polygon is selected independently, different bootstrap iterations will have different numbers of ADMIN0 polygons that have all their descendants removed. In fact, there is no guarantee that any ADMIN0 polygons are selected. We also note that we did not run 100 separate bootstraps of the machine learning prevalence models. Given their role as fixed covariates, like the modelled and gap-filled environmental covariates, we deemed this unnecessary.

2.2.7.2.10 Age splitting

Finally, incidence and PR estimates were age-split into three bins (0–5; 5–14; >15) using previously published models.[17]

3 Mortality

3.1 Data

Data for *P. falciparum* and *P. vivax* mortality models included vital registration, verbal autopsy, and surveillance data obtained from the GBD Cause of Death (CoD) Database.[39] The CoD database provides cause-specific mortality information at both site and administration unit levels. Variability in the type and abundance of CoD necessitated distinct approaches for estimating malaria mortality for (i) *P. falciparum* inside Africa, (ii*) P. falciparum* outside Africa, and (iii) *P. vivax* in countries without *P. falciparum.*

The CoD database provides us with 4750 unique location-years of data from endemic countries, 280 of which were from sSA. No data were available after 2020 meaning that the impact of the COVID-19 pandemic is not reflected in the input data for the mortality model (Figure S11).

Inside of sSA, the majority of data were from verbal autopsy, whilst outside of sSA data were primarily from vital registration. Whilst the quantity and quality of malaria routine surveillance data from Health Management Information Systems (HMIS) has improved greatly over the last decade,[42, 43] there are still major limitations in its usage for malaria mortality estimation, particularly in sSA. These are primarily: (i) limited representativeness of the data, (ii) difficulties in defining populations covered by surveillance (i.e., the denominator), (iii) incomplete testing for malaria, (iv) incomplete recording and reporting of data, (v) lack of data from private health facilities and (vi) a general under-reporting of malaria deaths in health facilities.[44-46] Potential reasons for this include complete autopsies not being performed; a failure to report deaths onto HMIS in an interpretable format; assigning the incorrect ICD-10 codes; and a reluctance to report malaria deaths. Due to these limitations, verbal autopsy was considered the best available data source for cause of death determination in sSA. Outside of sSA, where HMIS and vital registration systems have been established longer, we use a mixture of verbal autopsy and vital registration data as an input for out mortality models, with vital registration being the primary data type (Table S15, Figure S11)

Table S15. Number of data sources by data type overall and in sSA.

Data Type	Number of data sources (n)	
	All malaria endemic locations	Sub-Saharan Africa
Minimally invasive tissue sampling (MITS)	1	1
Verbal autopsy (VA)	98	64
Vital registration (VR) including sample VR	227	17

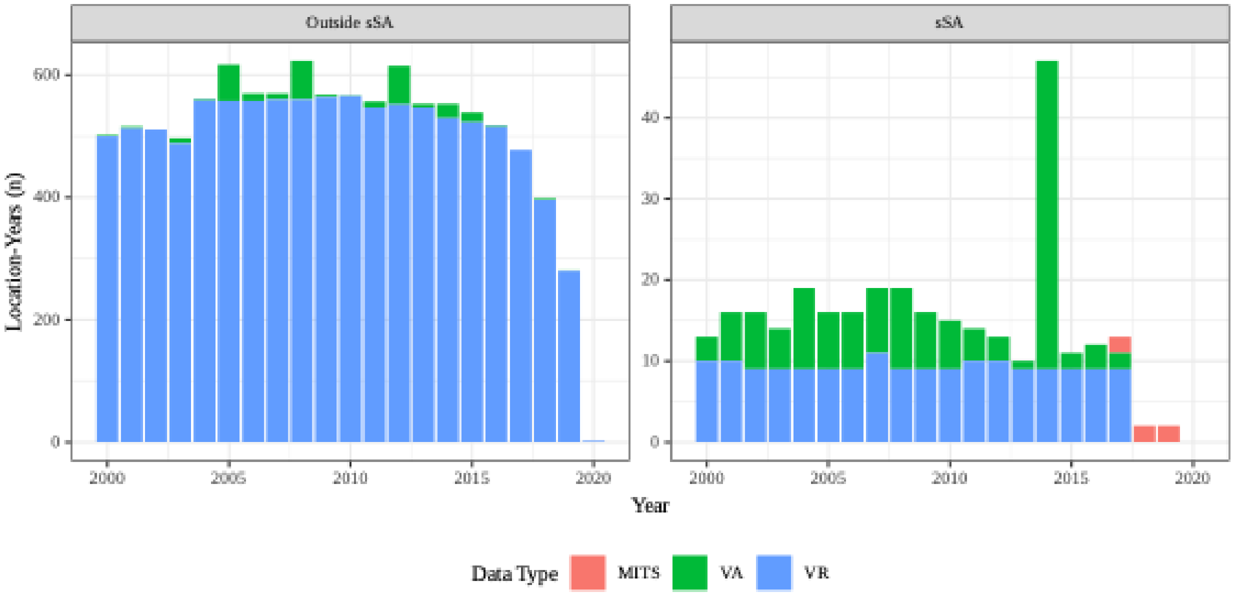

Figure S11. Number of locations of CoD data by year and data type for sSA and outside of sSA.

3.2 Methods

3.2.1 Mortality estimation in cartographic and surveillance countries

3.2.1.1 Untreated incidence

We estimated effective treatment of malaria by firstly estimating the proportional usage of artemisinin combination therapy (ACT) and non-ACT drugs for each country-year. This was combined with estimates of drug efficacy[24] and treatment-seeking to create a spatiotemporal cube of effective treatment with an antimalarial drug.[25] These were combined with the previously described estimates of *P. falciparum* incidence to derive estimates of the incidence of untreated malaria for each 5km x 5km grid cell and year.

3.2.1.2 Case fatality rate

For each site-year for which CoD malaria cause fraction data were available we (i) estimated a site, year, age-group (0-4 years, 5-14 years, 15+ years) and sex specific malaria mortality rate, as the product of malaria cause fraction and all-cause mortality rate (with the later drawn from national-level values); (ii) divided the malaria mortality rate by the site-year-age specific estimates of untreated malaria incidence rate (non-sex specific) to estimate a site-year-age-sex specific case fatality rate (CFR) amongst untreated malaria cases. These derived CFR values were then used in a mixed-effects regression model to estimate the yearly CFR for each 5km × 5km grid cell. The covariates used in the model were the log of country-year all-cause mortality rate, night-time lights, accessibility and fractional land-cover classes, and study-specific age-group and sex, with the location of each study site included as a national-level random effect. The model was fit in a Bayesian framework using the "R2BayesX" package in R. Further details can be found in previous publications.[47]
A second model was used in surveillance countries using a similar approach. The rationale for differing models two-fold: (i) vastly different rates of malaria mortality and data availability between high and low burden countries made

it difficult to fit a model that could accurately predict across all endemicity levels; and (ii) unified (i.e., global) models led to results for SSA that diverted substantially from earlier GBD cycles. Given these two factors, we fit a second model for surveillance countries. We calculated the CFR for untreated malaria by site-year for all ages and sexes combined, following the previously described methodology. These values were used in a mixed effects regression model to estimate the yearly CFR for each 5km x 5km grid cell. The covariates used in this model were log of country-year all-cause mortality rate, accessibility, proportion of the population as adults, proportion of the population as infants and sickle-cell anaemia prevalence, with a national-level random effect on the study location site. This model was fit in a Bayesian framework using the “INLA” package in R.

3.2.1.3 Deriving mortality estimates

To estimate the fatal burden of *P. falciparum* malaria, the CFR estimates derived for untreated cases were applied to our estimates of untreated incidence to produce the GBD death estimates. All estimates were created for a set of 1000 draws per admin-unit-year to support derivation of summary statistics including uncertainty intervals. Given the infrequency of deaths caused by *P. vivax*, and the absence of consensus on its mortality rate, death estimates were only derived for *P. falciparum*, except for country-years in which only *P. vivax* cases were reported. In such cases, a simple regression model of the site-year specific number of malaria deaths was fit, given the covariates of study year, sex and age-group, and the log of country-year all-cause mortality rate. The model was fit using the “BayesX” package in R.

3.2.2 Age splitting mortality results

Our mortality estimation approach yields all-age death results, but the GBD project requires age-split estimates. To generate age splits, we used a traditional cause of death ensemble model (CODEm), which is the standard model used for estimate deaths for most causes within GBD. The model included the following covariates: *P. falciparum* incidence rate and effective treatment with an antimalarial drug (AM). We derive age-specific to all-age ratios from the CODEm results and applied them to our all-age death estimates across all realisations. The aggregated summary statistics derived from these age-split outputs ultimately constitute the required GBD national or subnational death estimates. Despite this step in our processing pipeline, we only present all-age death estimates here and through the MAP data portal (https://data.malariaatlas.org/). Age split results are available through GBD, but only at GBD location-level.

3.2.3 Raking death estimates to match GBD results

Malaria mortality estimates both inside and outside of Africa were aligned to the malaria death results from the GBD2021 by raking (i.e. linearly scaling up or down) them over our modelled mortality surfaces. This process preserved the spatial heterogeneity from earlier models while taking advantage of the GBD approach for balancing all causes of death within a single model. In brief, the GBD estimates were generated at the administrative level by taking the mortality estimates from all causes of deaths in the study (including those for malaria that we produced), and then applying the CodCorrect model to ensure that the sum of deaths for all causes matched the modelled total from an all-cause mortality envelope. This approach reflected a key principle of the GBD to ensure internal consistency in mortality estimates across causes and within the all-cause envelope. One consequence is that the original modelled link between malaria case incidence and malaria mortality is decoupled to enable the latter to be adjusted where required by the CodCorrect process. This, in turn, caused some areas with low-malaria burden to have implied CFRs that are outside expected ranges (i.e., implausible mortality given the modelled incidence). This was considered preferable than the possible alternatives of (a) omitting the CodCorrect step or (b) making post-hoc adjustments to underlying case incidence estimates to preserve originally modelled CFRs.

4 Schematic diagrams

Prevalence and incidence modelling (cartographic)

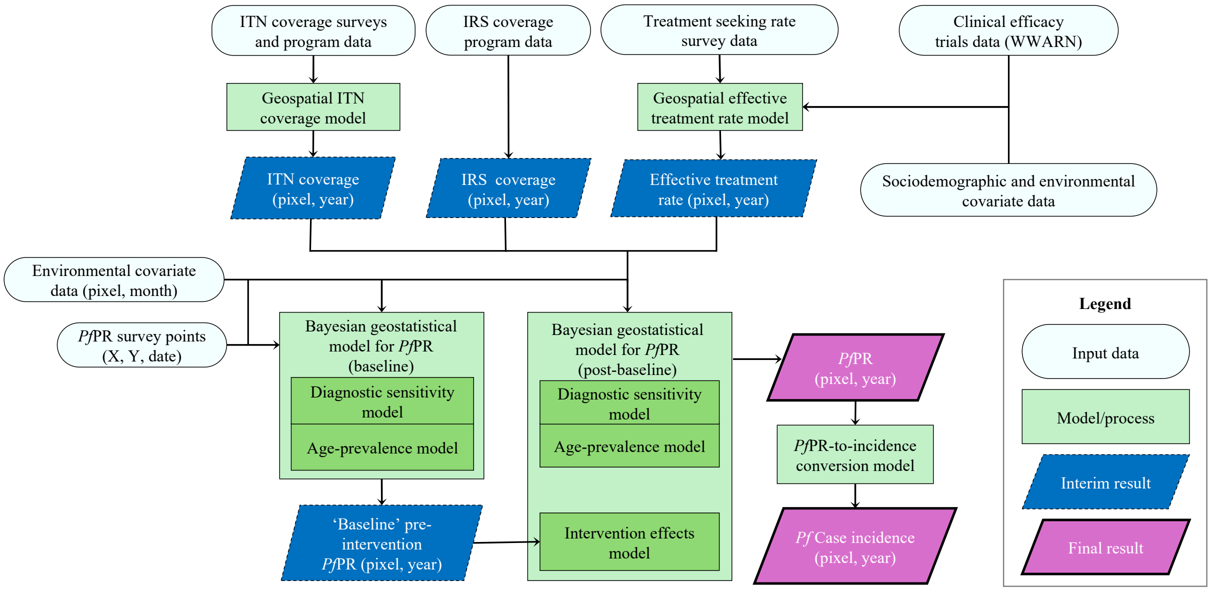

Figure S12. Flowchart of the prevalence and incidence modelling process for cartographic countries.

Prevalence and incidence modelling (surveillance)

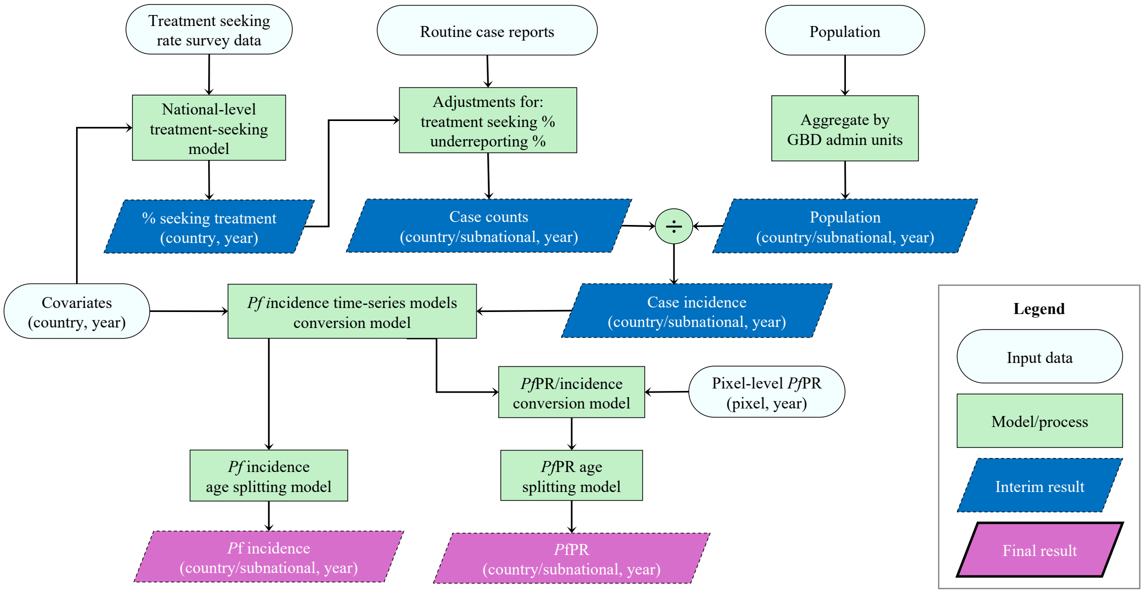

Figure S13. Flowchart of the prevalence and incidence modelling process for surveillance countries. Identical methodology was used for *P. falciparum* (shown in diagram) and *P. vivax*.

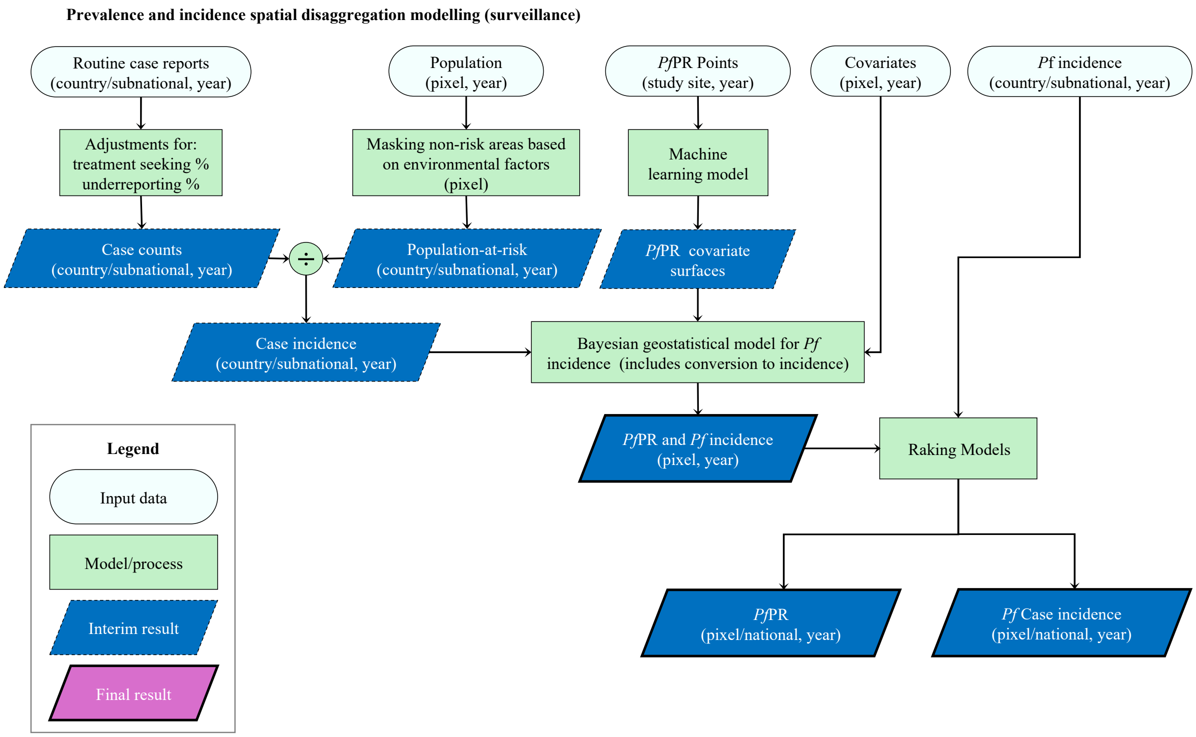

Figure S14. Flowchart of the spatial disaggregation modelling process for surveillance countries. Identical methodology was used for *P. falciparum* (shown in diagram) and *P. vivax*.

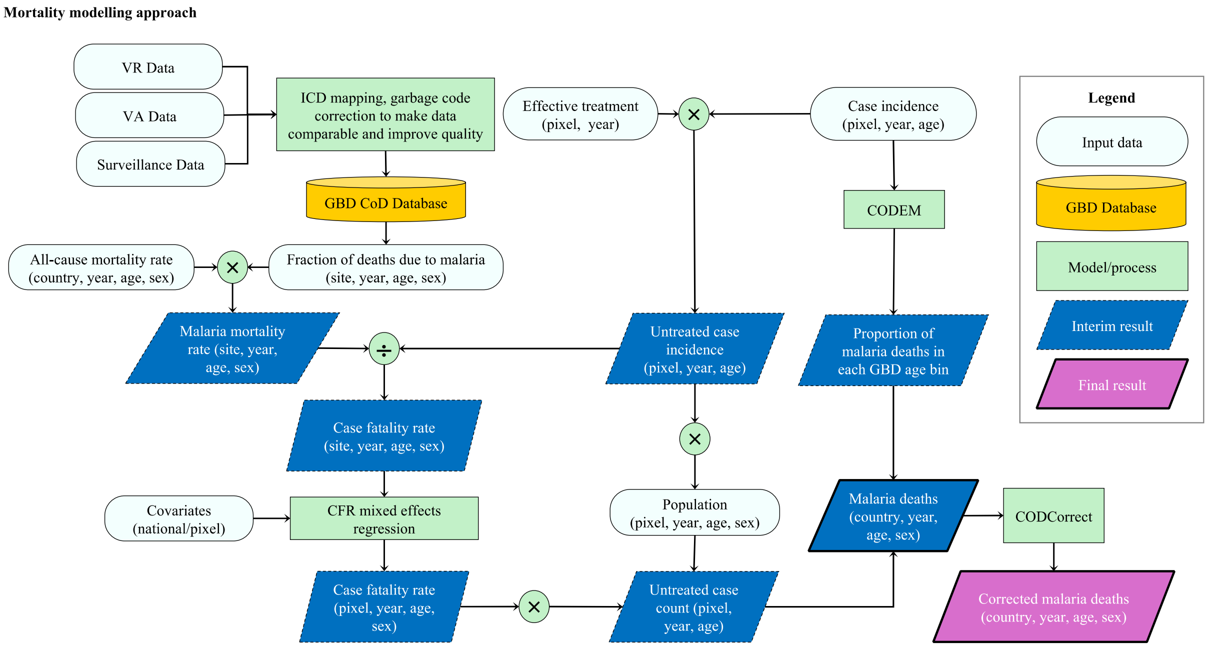

Figure S15. Flowchart of the mortality modelling process for cartographic countries.

5 GATHER compliance

5.1 Checklist

Table S16: GATHER compliance checklist.

Item No.	Checklist item	Reference
	**Objectives and funding**	
**1**	Define the indicator(s), populations (including age, sex, and geographic entities), and time period(s) for which estimates were made.	Main text (Abstract, Introduction – pages 1-3)
**2**	List the funding sources for the work.	Main text (funding statement)
	**Data Inputs**	
	From multiple sources that are synthesised as part of the study:	
**3**	Describe how the data were identified and how the data were accessed.	Main text (Methods), Supplementary Information (Sections 2.1 and 3.1)
**4**	Specify the inclusion and exclusion criteria. Identify all ad-hoc exclusions.	Supplementary Information (Section 2.2)
**5**	Provide information on all included data sources and their main characteristics. For each data source used, report reference information or contact name/institution, population represented, data collection method, year(s) of data collection, sex and age range, diagnostic criteria or measurement method, and sample size, as relevant.	Supplementary Information (Sections 2.1 and 3.1) and online data citation tools from https://ghdx.healthdata.org/gbd-2021/sources and https://github.com/malaria-atlas-project/malariaAtlas
**6**	Identify and describe any categories of input data that have potentially important biases (e.g., based on characteristics listed in item 5).	Main text (Discussion), Supplementary Information (2.1 and 3.1)
	Which contribute to the analysis but were not synthesised as part of the study:	
**7**	Describe and give sources for any other data inputs.	Supplementary Information (section1.1)
	For all data inputs:	
**8**	Provide all data inputs in a file format from which data can be efficiently extracted (e.g., a spreadsheet rather than a PDF), including all relevant meta-data listed in item 5. For any data inputs that cannot be shared because of ethical or legal reasons, such as third-party ownership, provide a contact name or the name of the institution that retains the right to the data.	https://ghdx.healthdata.org/gbd-2021/sources and https://github.com/malaria-atlas-project/malariaAtlas
	**Data analysis**	
**9**	Provide a conceptual overview of the data analysis method. A diagram may be helpful.	Main text (Methods)
**10**	Provide a detailed description of all steps of the analysis, including mathematical formulae. This description should cover, as relevant, data cleaning, data pre-processing, data adjustments and weighting of data sources, and mathematical or statistical model(s).	Supplementary Information (sections 2.2 and 3.2)
**11**	Describe how candidate models were evaluated and how the final model(s) were selected.	Main text (Methods) and Supplementary Information (sections 2.2 and 3.2)
**12**	Provide the results of an evaluation of model performance, if done, as well as the results of any relevant sensitivity analysis.	Supplementary Information (sections 2.2 and 3.2)
**13**	Describe methods for calculating uncertainty of the estimates. State which sources of uncertainty were, and were not, accounted for in the uncertainty analysis.	Supplementary Information (Sections 2.2 and 3.2)
**14**	State how analytic or statistical source code used to generate estimates can be accessed.	Main text (Results)

	**Results and Discussion**	
**15**	Provide published estimates in a file format from which data can be efficiently extracted.	Available from https://github.com/malaria-atlas-project/malariaAtlas and https://data.malariaatlas.org/maps
**16**	Report a quantitative measure of the uncertainty of the estimates (e.g. uncertainty intervals).	Main text (Results)
**17**	Interpret results in light of existing evidence. If updating a previous set of estimates, describe the reasons for changes in estimates.	Main text (Discussion)
**18**	Discuss limitations of the estimates. Include a discussion of any modelling assumptions or data limitations that affect interpretation of the estimates.	Main text (Discussion)

6 References

1. Friedl M, Sulla-Menashe D. MCD12Q1 MODIS/Terra+Aqua Land Cover Type Yearly L3 Global 500m SIN Grid V006 [Data set]. NASA EOSDIS Land Processes Distributed Active Archive Center 2019. Accessed 2024-07-08 from https://doi.org/10.5067/MODIS/MCD12Q1
2. Schaaf C, Wang Z. MCD43D62 MODIS/Terra+Aqua BRDF/Albedo Nadir BRDF-Adjusted Ref Band1 Daily L3 Global 30ArcSec CMG V006 [Data set]. NASA EOSDIS Land Processes Distributed Active Archive Center 2015. Accessed 2024-07-08 from https://doi.org/10.5067/MODIS/MCD43D62.006
3. Wan Z, Hook S, Hulley G. MOD11A2 MODIS/Terra Land Surface Temperature/Emissivity 8-Day L3 Global 1km SIN Grid V006 [Data set]. NASA EOSDIS Land Processes Distributed Active Archive Center 2015. Accessed 2024-07-08 from https://doi.org/10.5067/MODIS/MOD11A2.006
4. Hijmans RJ, Cameron SE, Parra JL, Jones PG, Jarvis A. Very high resolution interpolated climate surfaces for global land areas. *Int J Climatol* 2005; **25**: 1965–78.
5. Weiss DJ, Nelson A, Gibson H, et al. A global map of travel time to cities to assess inequalities in accessibility in 2015. *Nature* 2018; **553**: 333–36.
6. Elvidge CD, Zhizhin M, Ghosh T, Hsu F-C, Taneja J. Annual time series of global VIIRS nighttime lights derived from monthly averages: 2012 to 2019. *Remote Sens* 2021; **13**: 922.
7. NASA JPL. NASA Shuttle Radar Topography Mission Global 3 arc second sub-sampled V003[Data set]. NASA EOSDIS Land Processes Distributed Active Archive Center 2013. Accessed 2024-07-08 from https://doi.org/10.5067/MEaSUREs/SRTM/SRTMGL3S.003
8. Zomer RJ, Trabucco A, Bossio DA, Verchot LV. Climate change mitigation: A spatial analysis of global land suitability for clean development mechanism afforestation and reforestation. *Agric Ecosyst Environ* 2008; **126**: 67–80.
9. Weiss DJ, Atkinson PM, Bhatt S, Mappin B, Hay SI, Gething PW. An effective approach for gap-filling continental scale remotely sensed time-series. *ISPRS J Photogramm Remote Sens* 2014; **98**: 106–18.
10. Wang H, Abbas KM, Abbasifard M, et al. Global age-sex-specific fertility, mortality, healthy life expectancy (HALE), and population estimates in 204 countries and territories, 1950-2019: a comprehensive demographic analysis for the Global Burden of Disease Study 2019. *Lancet* 2020; **396**: 1160–203.
11. Tatem AJ. WorldPop, open data for spatial demography. *Sci Data* 2017; **4**: 170004. https://doi.org/10.1038/sdata.2017.4
12. Gething PW, Patil AP, Smith DL, et al. A new world malaria map: *Plasmodium falciparum* endemicity in 2010. *Malar J* 2011; **10**: 1–16.
13. Bhatt S, Weiss D, Cameron E, et al. The effect of malaria control on *Plasmodium falciparum* in Africa between 2000 and 2015. *Nature* 2015; **526**: 207–11.
14. Guerra CA, Hay SI, Lucioparedes LS, et al. Assembling a global database of malaria parasite prevalence for the Malaria Atlas Project. *Malar J* 2007; **6**: 1–13.
15. The DHS Program. The DHS Program. 2017. Accessed 2024-07-08 from http://www.dhsprogram.com/
16. UNICEF. Multiple indicator cluster survey (MICS). Accessed 2024-07-08 from https://mics.unicef.org/
17. Cameron E, Battle KE, Bhatt S, et al. Defining the relationship between infection prevalence and clinical incidence of *Plasmodium falciparum* malaria. *Nat Commun* 2015; **6** : 8170.
18. Welkhoff PA, Wenger EA. The EMOD Individual-Based Model. In: Arifin SMN, Madey GR, Collins FH, book authors. Spatial Agent-Based Simulation Modeling in Public Health. Hoboken, New Jersey (USA) : Wiley; 2016. p. 185-208.

19. Reiker T, Golumbeanu M, Shattock A, et al. Emulator-based Bayesian optimization for efficient multi-objective calibration of an individual-based model of malaria. *Nat Commun* 2021; **12**: 7212.
20. Griffin JT, Ferguson NM, Ghani AC. Estimates of the changing age-burden of *Plasmodium falciparum* malaria disease in sub-Saharan Africa. *Nat Commun* 2014; **5**: 3136.
21. Maire N, Smith T, Ross A, Owusu-Agyei S, Dietz K, Molineaux L. A model for natural immunity to asexual blood stages of *Plasmodium falciparum* malaria in endemic areas. *Am J Trop Med Hyg* 2006; **75**: 19-31.
22. Eckhoff P. *P. falciparum* infection durations and infectiousness are shaped by antigenic variation and innate and adaptive host immunity in a mathematical model. *PLoS One* 2012; **7**: e44950.
23. Battle KE, Guerra CA, Golding N, et al. Global database of matched *Plasmodium falciparum* and *P. vivax* incidence and prevalence records from 1985–2013. *Sci Data* 2015; **2**: 1–12.
24. Rathmes G, Rumisha SF, Lucas TC, et al. Global estimation of anti-malarial drug effectiveness for the treatment of uncomplicated *Plasmodium falciparum* malaria 1991–2019. *Malar J* 2020; **19**: 1–15.
25. Nguyen M, Dzianach PA, Castle PE, et al. Trends in treatment-seeking for fever in children under five years old in 151 countries from 1990 to 2020. *PLOS Glob Public Health* 2023; **3**: e0002134.
26. Dzianach PA, Rumisha SF, Lubinda J, et al. Evaluating COVID–19–Related Disruptions to Effective Malaria Case Management in 2020–2021 and Its Potential Effects on Malaria Burden in Sub–Saharan Africa. *Trop Med Infect Dis* 2023; **8**.
27. Cibulskis RE, Aregawi M, Williams R, Otten M, Dye C. Worldwide incidence of malaria in 2009: estimates, time trends, and a critique of methods. *PLoS Med* 2011; **8**: e1001142.
28. World Health Organization. World Malaria Report. Geneva, 2013.
29. World Health Organization. World Malaria Report. Geneva, 2015.
30. World Health Organization. World Malaria Report. Geneva, 2022.
31. World Health Organization. World Malaria Report. Geneva, 2023.
32. Kristensen K, Nielsen A, Berg CW, Skaug H, Bell BM. TMB: automatic differentiation and Laplace approximation. *J Stat Softw* 2016; **70**: 1–21.
33. Kuhn M, Wing J, Weston S, et al. caret: Classification and Regression Training. 2017 https://CRAN.Rproject.org/package=caret
34. Zou H, Hastie T. elasticnet: Elastic-Net for Sparse Estimation and Sparse PCA. 2012 https://CRAN.Rproject.org/package=elasticnet
35. Liaw A, Wiener M. Classification and regression by randomForest. *R news* 2002; **2**: 18–22.
36. Venables B, Ripley B. Modern Applied Statistics With S. Statistics and Computing. 4th ed. New York: *Springer-Verlag*; 2002.
37. Ridgeway GDE, Kriegler B, Schroedl S, et al. gbm: Generalized boosted regression models. *R package* version 2019. 37–40.
38. Ferrari AJ, Santomauro DF, Aali A, et al. Global incidence, prevalence, years lived with disability (YLDs), disability-adjusted life-years (DALYs), and healthy life expectancy (HALE) for 371 diseases and injuries in 204 countries and territories and 811 subnational locations, 1990–2021: a systematic analysis for the Global Burden of Disease Study 2021. *Lancet* 2024; **403**: 2133–61.
39. Naghavi M, Ong KL, Aali A, et al. Global burden of 288 causes of death and life expectancy decomposition in 204 countries and territories and 811 subnational locations, 1990–2021: a systematic analysis for the Global Burden of Disease Study 2021. *Lancet* 2024; **403**: 2100–32.
40. Sturrock HJ, Cohen JM, Keil P, et al. Fine-scale malaria risk mapping from routine aggregated case data. *Malar J* 2014; **13**: 1–9.
41. Lindgren F, Rue H, Lindström J. An Explicit Link between Gaussian Fields and Gaussian Markov Random Fields: The Stochastic Partial Differential Equation Approach. *J R Stat Soc Series B Stat Methodol* 2011; **73**: 423–98.
42. Muhoza P, Tine R, Faye A, et al. A data quality assessment of the first four years of malaria reporting in the Senegal DHIS2, 2014–2017. *BMC Health Serv Res* 2022; **22**.
43. Ouédraogo M, Kangoye DT, Samadoulougou S, Rouamba T, Donnen P, Kirakoya-Samadoulougou F. Malaria case fatality rate among children under five in Burkina Faso: an assessment of the spatiotemporal trends following the implementation of control programs. *Int J Environ Res Public Health* 2020; **17**: 1840.
44. Ashton RA, Bennett A, Yukich J, Bhattarai A, Keating J, Eisele TP. Methodological Considerations for Use of Routine Health Information System Data to Evaluate Malaria Program Impact in an Era of Declining Malaria Transmission. *Am J Trop Med Hyg* 2017; **97**: 46-57.
45. Wangdi K, Sarma H, Leaburi J, McBryde E, Clements ACA. Evaluation of the malaria reporting system supported by the District Health Information System 2 in Solomon Islands. *Malar J* 2020; **19**.

46. Alegana VA, Okiro EA, Snow RW. Routine data for malaria morbidity estimation in Africa: challenges and prospects. *BMC Med* 2020; **18**.

47. Gething PW, Casey DC, Weiss DJ, et al. Mapping *Plasmodium falciparum* mortality in Africa between 1990 and 2015. *N Engl J Med 2016*; **375**: 2435–45.
